# Estimating the Economic Value of Supported Online Parent-Led Cognitive Behavioral Therapy to Prevent Child Anxiety Problems: Economic Evaluation of the MYCATS Cluster Randomized Controlled Trial

**DOI:** 10.2196/87123

**Published:** 2026-10-08

**Authors:** Shuye Yu, Tessa Reardon, Obioha C Ukoumunne, Rebecca Njuguna, Helen Dodd, Gemma Halliday, Clare Hill, Bec Jasper, Benjamin Jones, Peter J. Lawrence, Fran Morgan, Anna Placzek, Ronald M Rapee, MYCATS Team, Emily Davey, Mara Violato, Cathy Creswell

**Affiliations:** 1 Nuffield Department of Population Health, University of Oxford, Old Road Campus, Oxford, England, OX3 7LF, United Kingdom, +44 (0)1865 289265; 2 Centre for Health Economics, University of York, York, England, United Kingdom; 3 Oxford Centre for Emerging Minds Research, Departments of Experimental Psychology and Psychiatry, University of Oxford, Oxford, England, United Kingdom; 4 Exeter Medical School, University of Exeter, Exeter, England, United Kingdom; 5 School of Psychology and Clinical Language Sciences, University of Reading, Reading, England, United Kingdom; 6 Parents and Carers Together, Suffolk, England, United Kingdom; 7 School of Psychology, University of Southampton, Southampton, England, United Kingdom; 8 Square Peg, Brighton and Hove, England, United Kingdom; 9 School of Psychological Sciences, Macquarie University, Sydney, Australia

**Keywords:** online intervention, digital application, parent-led CBT, cost-effectiveness analysis, economic evaluation, randomized controlled trial, child health, anxiety disorders, preventive health services, cognitive behavioral therapy

## Abstract

**Background:**

Childhood anxiety disorders are common and lead to substantial negative long-term outcomes in various life domains. Although digital interventions for preventing childhood anxiety problems have been increasingly used in the recent decade, there is limited evidence documenting their cost-effectiveness (CE).

**Objective:**

This study aimed to evaluate the CE of a parent-led online cognitive behavioral therapy with therapist support (OSI+TS) alongside usual school practice (USP), compared to USP alone for children aged 4‐7 years at risk of anxiety disorders identified through universal school screening.

**Methods:**

The economic evaluation was conducted alongside the MYCATS trial (N=865), a parallel group, superiority cluster randomized trial across 95 primary or infant schools in England. Schools were randomized (1:1) to OSI+TS or USP, stratified by school-level deprivation. The primary analyses, at 12 months after randomization, considered child quality-adjusted life years (QALYs) as the health economic outcome, using both the UK adult and the Australia adolescent value sets, and costs were from the National Health Service and Personal Social Services perspective. Incremental cost-effectiveness ratios (ICERs) were compared to the UK National Institute for Health and Care Excellence (NICE) recommended threshold of £20,000-£30,000 (£1=US $1.35 as of September 10, 2026) per QALY gained, as per predefined Health Economics Analysis Plan. Sensitivity analyses included a societal perspective and the application of the new NICE cost-effectiveness threshold.

**Results:**

Children receiving OSI+TS gained 0.021 (95% CI 0.010-0.033; *P*<.001) and 0.043 (95% CI 0.021-0.064; *P*<.001) QALYs, respectively, for United Kingdom and Australia value sets, with an additional cost of £244.16 compared to school usual practice only, resulting in ICERs of £11,462.47 and £5,700.98, respectively. At the £20,000 threshold, OSI+TS had an 88.2% or 99.3% chance of being cost-effective.

**Conclusions:**

OSI+TS is cost-effective compared to usual school provision, supporting its future implementation for children at risk of anxiety problems.

## Introduction

Over a quarter of the population experience anxiety problems at some point in their life [1]. The peak age of onset is as early as 5.5 years [2], and early anxiety problems are associated with poor outcomes across at least 15 domains of daily life, including other mental health problems (eg, depression, attention-deficit-hyperactivity disorder, and substance use), educational attainment, and unemployment [3]. Anxiety problems impose a broad burden on children, their families, and society, causing an annual societal cost up to £4040 (£1=US $1.35 as of September 10, 2026) per child with anxiety problems [3]. Prevention and early intervention are therefore necessary to mitigate the long-lasting negative consequences and costs of anxiety problems in childhood.

In recent years, the sharp rise in demand for child mental health services has made it difficult to provide timely support to struggling children. In England, for instance, yearly referrals to Child and Adolescent Mental Health Services (CAMHS) have increased by 53% since 2019, reaching more than 1.2 million in 2022 [4]. Consequently, many children are denied prompt support, and waiting times are increasingly long.

Digital health interventions represent scalable tools that have the potential to improve mental health outcomes for children and young people, while also widening access and reducing the gap between the provision and the demand for mental health services [5]. A number of digital interventions for child anxiety problems have recently been developed, but treatments are more prevalent than preventive interventions. A recent review by Wozney et al (2018) [6] assessed, within the Implementation Outcomes Framework by Proctor et al (2011) [7], implementation outcomes (such as acceptability, fidelity, appropriateness, and cost) of online interventions for children’s and young people’s anxiety and depression problems. Among the 23 digital programmes for anxiety problems in children and young people reviewed by Wozney et al, 2018 [6], only one of them was designed as preventive, and the authors found that positive outcomes in acceptability, adherence, and appropriateness were reported for more than 60% of the interventions. However, across all studies, the outcome cost was one of the least measured.

In addition to the limited number of available preventive digital interventions for childhood anxiety problems, economic evaluations of these technologies are even sparser. Our systematic search of the literature (section 1 in Multimedia Appendix 1) revealed that most studies of digital interventions for childhood anxiety problems report on effectiveness, with limited evidence on economic outcomes. The recent systematic review by Vartiainen et al [8] identified only 5 studies reporting on the cost-effectiveness (CE) of preventive interventions for child and adolescent anxiety disorders, four of which were cost-effective, but none involved digital approaches [8]. Given the important role played by economic evaluations in ensuring the efficient allocation of limited health care resources, it is crucial that such evidence is provided alongside evidence of the effectiveness and acceptability of digital interventions.

The Minimising Young Children’s Anxiety through Schools (MY-CATS) trial compared parent-led cognitive behavioral therapy (CBT) delivered online with therapist telephone support (OSI+TS) alongside usual school provision, to usual school provision only. In the trial, 865 children aged 4 to 7 years across 95 schools in England were identified at risk of anxiety disorders based on child anxiety symptoms, inhibition, and/or parent anxiety symptoms. While the overall frequency of anxiety problems 12 months after randomization was low and group differences were not significant in the primary clinical analysis (89/865, 6.8% intervention vs 99/865, 11.5% usual school practice [USP]), the intervention was superior to usual school practice across all secondary clinical outcomes that included anxiety and related symptoms, behavioral symptoms, and measures of risk for future anxiety problems [9].

This study builds on economic data collected alongside the MYCATS trial and addresses the gap in the economic evidence-base by evaluating whether OSI+TS, on top of USP, is cost-effective compared with USP in early prevention or treatment of child anxiety problems. Our work adds to the ongoing efforts to prevent anxiety problems and the adverse consequent effects on children, their families, and society.

## Methods

### Study Design and Participants

This is an economic evaluation conducted alongside the MYCATS study, a parallel group, superiority cluster randomized controlled trial, with schools (clusters) randomized to the intervention or USP arm in a 1:1 ratio stratified by school-level deprivation and balanced on cluster size. The economic evaluation reports on both a cost-utility analysis (CUA: primary analysis) and a CE analysis (CEA: secondary analysis) by measuring and valuing health outcomes and resource use from baseline to 12 months after randomization. A prespecified health economic analysis plan was developed prior to analysis (section 2 in Multimedia Appendix 1) [10]. All economic analyses were performed according to best practice for conducting and reporting economic evaluations [11,12]. The study population included all children and families participating in the trial, according to an intention-to-treat (ITT) approach. Inclusion criteria are reported in section 3 in Multimedia Appendix 1. Schools were recruited in 5 batches.

### Ethical Considerations

Written consent was obtained from parents of all children participating in the trial. Ethical approval for the study, including the economic evaluation, was obtained from the University of Oxford Medical Science Interdivisional Research Ethics Committee (reference R62531). The study was prospectively registered on International Standard Randomized Controlled Trial Number (ISRCTN; 82398107) and the protocol was published [13].

### Intervention

OSI+TS is an online adaptation of a brief therapist-supported, parent-delivered CBT program for child anxiety disorders [14,15]. It was shown to be clinically noninferior and likely to be cost-effective, compared to treatment as usual for children aged 5 to 12 years with anxiety disorders in UK CAMHS [16,17]. This trial focused on children aged 4‐7 years who screened positive for at least one risk (elevated anxiety symptoms, and/or inhibition, and/or parent anxiety) for anxiety disorders. The intervention provided parents of participating children with an age-adapted version of OSI+TS, containing 7 weekly modules, which empowered them to acquire CBT-based skills and strategies to prevent and/or provide early treatment for anxiety problems in their children. Content was presented in an accessible way, using simple text, videos, animations, interactive activities, and questionnaires. Alongside the online modules, parents received support from a therapist (typically a children’s well-being practitioner) through 8 telephone sessions (referred to as parent support sessions). The first 7 support sessions were held weekly, after the parent had worked through each weekly online module, and were expected to last for about 20 minutes. The eighth session was a final review telephone call, which was scheduled approximately 4 weeks after the last OSI module. Regardless of arm, the trial did not prevent children from receiving any other usual mental health support offered by their school or other providers.

### Outcomes

The primary health economic outcome was child quality-adjusted life years (QALYs) derived from the proxy version (parent-report on child) of the Child Health Utility 9-Dimension (CHU9D) instrument [18-20], measured at baseline, 12 weeks, and 12 months after randomization. The CHU9D is a validated, preference-based measure of child health-related quality of life (HRQoL), including 9 questions on a child’s daily functioning. Responses can be transformed into health-related quality of life scores, called utilities, using published preference weights (or value sets). Utility scores range from 0 to 1, where 0 represents a health state considered equivalent to being dead, and 1 represents full health. Higher utility scores indicate better health-related quality of life. Two sets of preference weights are available: one derived from a sample of the UK adult general population [19] and the other from Australian adolescents aged 11 to 17 years [21] (hereon referred to as the UK adult and Australia adolescent value sets, respectively). As no established guideline indicates which value set is more appropriate, we conducted all analyses using both. Parent’s QALYs were derived from the EQ-5D-5L instrument, an adult preference-based measure of HRQoL [22]. Parent’s utilities were obtained from the EQ-5D-5L instrument using a validated algorithm recommended by the UK National Institute of Health and Care Excellence (NICE) [11]. Child and parent QALYs were then calculated separately by combining utility values at the 3 assessment points, using the area-under-the-curve method. QALYs for the parent-child dyad were used as the outcome in some sensitivity analyses and were generated by combining child and parent QALYs additively. The secondary outcome was the primary clinical outcome, a binary variable indicating the absence (=0) or presence (=1) of an anxiety disorder at 12 months after randomization, assessed using the Anxiety Disorder Interview Schedule for Children-Parent Interview (ADIS-P) [23]. Negative mean differences in the ADIS-P indicate a benefit in favor of the intervention arm, while positive mean differences favor the control arm.

Resource use was collected at participant-level and included intervention, additional health and personal social service use, absenteeism from school for children and from work for parents. To allow calculation of the total cost of intervention, therapists completed bespoke intervention logs, where they recorded time spent delivering the intervention, and both therapists and supervisors filled in supervision logs. Both intervention and supervision logs included details of time spent for preparation and administrative tasks (eg, writing notes after a session).

Child and parent health and personal social service use were collected retrospectively from parents using a modified (with Patient and Public Involvement and Engagement) version of the Client Service Receipt Inventory (CSRI) at baseline (with reference to the prior 3 months), 12 weeks, and 12 months after randomization [24]. The CSRI included information about use of primary and secondary care services, and medications, which were included in the National Health Service (NHS) and Personal Social Services (PSS) perspective analysis, but also details on travel time and costs associated with attending services, other out-of-pocket costs, days off school for children and from work for parents, which were used in the societal perspective analyses.

Resource use was valued using appropriate unit costs (Tables S5.1-S5.6 and S6.1-S6.2 in Multimedia Appendix 1). Absence from school was costed both as an opportunity cost for the education system and a loss of human capital (section 4 in Multimedia Appendix 1).

### Data Analyses

The detailed, prespecified Health Economics Analysis Plan (HEAP) is included in section 2 in Multimedia Appendix 1. As per NICE guidelines [11], the primary analysis (base-case analysis) was a CUA of OSI+TS compared to USP undertaken from the NHS and PSS perspective, using an ITT approach. Child QALYs were derived using both the UK adult and the Australia adolescent value sets. Mean (SD) utilities for children and parents were reported by trial arm for each assessment point. Mean (SD) intervention and supervision-related time were reported for the intervention group. Other child and parent resources used were reported by trial arm, using mean, SD, range, and the percentage of respondents who reported at least one use per type of resource. They were not compared statistically to avoid problems of multiple testing and in accordance with the focus of the economic analysis being on cost and CE [25]. For each trial participant, resource use data were multiplied by their unit cost to generate the total mean cost in each trial arm. Costs were expressed in pound sterling (£) in 2021-2022 prices. Consistent with standard practice for economic evaluations with a 12-month time horizon, discounting for time preference was not applied because all costs and health outcomes were incurred within one year [11,26,27]. A secondary CE analysis was undertaken, with outcomes measured using the difference in the presence of an anxiety disorder at 12 months and incremental costs from the NHS and PSS perspective (secondary base-case analysis).

Individual missing items were imputed using mean imputation, conditional on trial arm and other relevant characteristics as appropriate. Multiple imputation was used for missing responses and cases, under the assumption of data missing at random [28]. In descriptive statistics, adjusted differences in HRQoL and cost between trial arms were calculated using an Ordinary Least Squares (OLS) model with standard errors clustered at the school level, based on 50 imputed datasets. All models were adjusted for baseline values and a set of covariates, including school batch, child gender, Index of Multiple Deprivation (IMD) decile, free school meals status (below or above national average percentage), year group (reception, year 1, or year 2), and number of children in the school recruited to the trial.

For the CUAs, the point estimates of incremental cost and QALY were obtained using a multilevel mixed-effects model accounting for school-level clustering based on 50 imputed datasets. To preserve the correlation between the two outcomes, the associated 95% CIs were constructed using a percentile approach based on the bootstrap distribution from 10,000 bootstraps (200 times on each of the 50 multiply imputed datasets). Incremental QALYs were combined with incremental costs to produce an incremental CE ratio (ICER). Uncertainty in the CE results was quantified based on the 10,000 bootstraps. The point estimates and the bootstrapped results were plotted on the CE-plane, and the probability that OSI+TS was cost-effective over a range of willingness-to-pay (WTP) values per QALY gained was reported using a CE acceptability curve (CEAC) [29]. In line with NICE guidelines at the time of the study, we used the £20,000-£30,000 (£1=US $1.35 as of September 10, 2026) threshold per QALY gained to determine the CE of the intervention. However, while this manuscript was under review, NICE updated their methods guidance to recommend a WTP threshold of £25,000‐35,000 per QALY gained [27]. Although the economic evaluation was conducted in accordance with our prespecified HEAP, we considered it appropriate to undertake an additional sensitivity analysis of the base-case using the revised threshold. Results were also reported in terms of net monetary benefit (NMB) and net health benefits (NHB) as recommended by NICE [11]. A similar approach was adopted in the CEAs, but the maximum threshold value that the NHS (health sector) or society are willing to pay for an extra child with no anxiety problems is not established, so results were presented over a range of potential values that a decision maker might be willing to pay to improve the outcome.

In addition to the base-case sensitivity analysis using the new WTP threshold, as explained above, we conducted 21 prespecified sensitivity analyses (SAs) to examine the robustness of the base-case analysis findings to different scenarios, including taking a societal perspective and using a per-protocol (PP) approach. In two further SAs, we included the three screening variables (child anxiety symptoms, behavioral inhibition, and parent or carer anxiety symptoms) as covariates to assess whether their slight imbalance prior to school-level randomisation affected the incremental analyses. SAs for the CUAs (n=20) were conducted using both the UK adult and the Australia adolescent value sets for child QALYs (Table S7-S8 in Multimedia Appendix 1). For the secondary CEA, we conducted three sensitivity analyses (Table S9 in Multimedia Appendix 1).

All analyses were implemented using STATA 18.0 (StataCorp) and R 4.3.1 software (R Core Team). Statistical significance was set at a 5% significance level.

## Results

Ninety-five schools were approached and agreed to participate in the study between February 8, 2021, and June 29, 2022. Participants were invited and screened from March 21, 2021, to July 21, 2022; and baseline assessments were administered from April 14, 2021, to July 28, 2022. Further details on participation rates are reported elsewhere [30], and the CONSORT (Consolidated Standards of Reporting Trials) flow diagram is reproduced in section 5 in Multimedia Appendix 1.

The full trial population consisted of 865 children with an average age of 6.2 (SD 0.9) years at randomization. Of these children, 432 (50%) were female and 751 (87%) were White (Table 1). The parents who completed the questionnaires were primarily mothers (803; 94%) and had a mean age of 36.9 (SD 5.5) years (Table S1.1 in Multimedia Appendix 1). School characteristics for the participating children are reported in Table S1.2 in Multimedia Appendix 1. Completeness of health economic outcomes data by trial arm and for each time point, and complete cases descriptive statistics on health economic outcomes are reported in Tables S2 and S3 in Multimedia Appendix 1, respectively.

**Table 1.** Selected baseline characteristics of participating children^a^.

Characteristics	OSI^b^+TS (n=434)	USP^c^ (n=431)	Total (N=865)
Batch, n (%)			
Cohort 1	51 (12)	58 (13)	109 (13)
Cohort 2	126 (29)	125 (29)	251 (29)
Cohort 3	118 (27)	118 (27)	236 (27)
Cohort 4	99 (23)	118 (27)	217 (25)
Cohort 5	40 (9)	12 (3)	52 (6)
Child year group (years)^d^, n (%)			
Reception, 4‐5	124 (29)	130 (30)	254 (29)
Year 1, 5‐6	152 (35)	145 (34)	297 (34)
Year 2, 6‐7	158 (37)	156 (36)	314 (36)
Child age (years), mean (SD)^e^	6.3 (0.9)	6.2 (0.9)	6.2 (0.9)
Child gender, n (%)			
Female	219 (50)	213 (49)	432 (50)
Male	215 (50)	218 (51)	433 (50)
Other	0 (0)	0 (0)	0 (0)
Child ethnicity, n/N(%)^f^			
Asian^g^	17 (4)	11/430 (3)	28/864 (3)
Black^h^	4 (<1)	5/430 (1)	9/864 (1)
Mixed^i^	35 (8)	34/430 (8)	69/864 (8)
White^j^	373 (86)	378/430 (88)	751/864 (87)
Other^k^	4 (<1)	0/430 (0)	4/864 (<1)
Prefer not to say	1 (<1)	2/430 (<1)	3/864 (<1)
Index of Multiple Deprivation decile, n (%)^l^			
1st	42 (10)	42 (10)	84 (10)
2nd	40 (9)	21 (5)	61 (7)
3rd	62 (14)	47 (11)	109 (13)
4th	39 (9)	29 (7)	68 (8)
5th	39 (9)	71 (16)	110 (13)
6th	51 (12)	43 (10)	94 (11)
7th	36 (8)	49 (11)	85 (10)
8th	50 (12)	31 (7)	81 (9)
9th	44 (10)	60 (14)	104 (12)
10th	31 (7)	38 (9)	69 (8)
Screening outcome, n (%)			
One risk	247 (57)	209 (48)	456 (53)
Two risks	149 (34)	165 (38)	314 (36)
Three risks	38 (9)	57 (13)	95 (11)
Screened positive for child anxiety	297 (68)	337 (78)	634 (73)
Screened positive for behavioral inhibition	126 (29)	132 (31)	258 (30)
Screened positive for parent anxiety	236 (54)	241 (56)	477 (55)

a Data were available for all randomized participants unless otherwise stated. This table is adapted from Table 2 of Reardon et al, 2026 [30], which examines the clinical results of the same trial (MYCATS). Full individual characteristics for children and their parents are reported in Table S1.1 in Multimedia Appendix 1.

b OSI+TS: online support and intervention for child anxiety with therapist support.

c USP: usual school practice.

d Year group at screening.

e Child age at randomization.

f Data were not available for all randomized participants.

g Includes Asian or Asian British Indian, Pakistani, any other Asian background.

h Includes Black or Black British African, Caribbean, any other Black background.

i Includes White and Black Caribbean, White and Black African, White and Asian, any other mixed background.

j Includes British, Irish, any other White background.

k Includes Chinese.

l Based on family postcode: 1=most deprived; 10=least deprived.

**Table 2.** Mean health-related quality of life and QALYs^a^ of children and parents.

Utilities and QALYs	OSI + TS (n=434)^b^, mean (SD)		USP (n=431)^c^ , mean (SD)	
Child CHU9D^d^ - UK value set:				
Baseline	0.856 (0.091)		0.841 (0.102)	
12 weeks	0.883 (0.096)		0.856 (0.101)	
12 months	0.886 (0.098)		0.870 (0.102)	
Total child QALYs	0.990 (0.108)		0.963 (0.112)	
Child CHU9D - AUS value set:				
Baseline	0.715 (0.198)		0.687 (0.214)	
12 weeks	0.776 (0.199)		0.716 (0.215)	
12 months	0.778 (0.205)		0.743 (0.215)	
Total child QALYs	0.865 (0.198)		0.809 (0.210)	
Parent EQ-5D-5L				
Baseline	0.781 (0.214)		0.788 (0.205)	
12 weeks	0.800 (0.222)		0.796 (0.225)	
12 months	0.794 (0.230)		0.818 (0.196)	
Total parent QALYs	0.893 (0.239)		0.902 (0.229)	

a QALY: quality-adjusted life year.

b OSI+TS: online support and intervention for child anxiety with therapist support.

c USP: usual school practice.

d CHU9D: Child Health Utility 9 Dimensions instrument.

Mean child CHU9D utilities, using both UK adult and Australia adolescent value sets, and mean parent EQ-5D-5L utilities at the three assessment points are reported in Table 2. At 12 weeks after randomization, child utilities were higher (ie, indicating better HRQoL) in the OSI+TS arm and the mean difference (Table 3) with the USP arm was statistically significant (adjusted mean difference, UK adult value set: 0.019, 95% CI 0.006-0.032; *P*=.004; Australia adolescent value set: 0.048; 95% CI 0.021-0.076; *P*=.001). Higher utilities were maintained in the intervention group at the 12 months follow-up, although the difference with the USP group narrowed and was no longer statistically significant. The mean difference in child QALYs between trial arms (Table 3) was statistically significant in favor of the OSI+TS children (adjusted mean difference: UK value set: 0.021, 95% CI 0.010-0.033; *P*<.001; Australia value set: 0.043, 95% CI 0.021-0.064; *P*<.001). In contrast, no statistically significant differences in parents’ EQ-5D-5L utilities and QALYs between trial arms were found throughout the trial.

**Table 3.** Mean difference in the health-related quality of life and QALYs^a^ of children and parents^b^.

Utilities and QALYs	Unadjusted difference, mean (95% CI)		Unadjusted difference, *P* value	Adjusted difference^c^, mean (95% CI)		Adjusted difference^c^, *P* value
Child CHU9D^d^ - UK value set						
Baseline	0.016 (-0.000 to 0.032)		.06	—^e^		—
12 weeks	0.027 (0.010 to 0.043)		.002	0.019 (0.006 to 0.032)		0.004
12 months	0.016 (0.001 to 0.031)		.04	0.010 (-0.003 to 0.023)		0.13
Total child QALYs	0.027 (0.009 to 0.045)		.003	0.021 (0.010 to 0.033)		<.001
Child CHU9D - AUS value set						
Baseline	0.028 (-0.006 to 0.062)		.11	—		—
12 weeks	0.061 (0.027 to 0.095)		.001	0.048 (0.021 to 0.076)		0.001
12 months	0.035 (0.003 to 0.066)		.03	0.024 (-0.003 to 0.051)		0.08
Total child QALYs	0.055 (0.023 to 0.088)		.001	0.043 (0.021 to 0.064)		<.001
Parent EQ-5D-5L						
Baseline	-0.008 (-0.041 to 0.026)		.66	—		—
12 weeks	0.003 (-0.035 to 0.041)		.86	0.011 (-0.011 to 0.034)		0.32
12 months	-0.025 (-0.060 to 0.010)		.16	-0.017 (-0.038 to 0.004)		0.11
Total parent QALYs	-0.009 (-0.048 to 0.030)		.65	0.002 (-0.016 to 0.021)		0.81

a QALY: quality-adjusted life year.

b The CI of the unadjusted and adjusted differences are constructed based on the standard errors of an OLS regression clustered at the school level.

c In the column “Adjusted difference,” we adjusted for the baseline value of each measure and for the following variables: school batch, children’s gender, number of children in the school recruited in the trial, year group, index of multiple deprivation decile, free school meal status.

d CHU9D: Child Health Utility 9 Dimensions instrument.

e Not applicable.

Time spent on intervention delivery amounted to a mean of 306 (SD 127) minutes, which included 152 (SD 70) minutes on parent support sessions, and 78 (SD 75) minutes on supervision (Table S4 in Multimedia Appendix 1). Descriptive statistics on individual resource use items from the CSRI are reported in Tables S5.1-S5.6 in Multimedia Appendix 1. In relation to costs (Tables 4 and 5), from the NHS and PSS perspective, children in the OSI+TS arm incurred £244.16 (95% CI £76.26-£412.07; *P*=0.005; £1=US $1.35 as of September 10, 2026) more than those in the USP arm. This was lower than the overall cost of OSI+TS intervention delivery and supervision that exclusively applied to the intervention group, and which cost £282.53 (SD 135.30) on average. Cost differences for other items were not statistically significant for either children or parents (Table 5). From a societal perspective, total societal mean difference in cost was £234.71 (95% CI £-165.37-£634.79; *P*=0.24) higher for the OSI+TS group compared to the USP group.

**Table 4.** Mean NHS and PSS^a^ and societal cost (in £) of Children and Parents.

Cost	OSI+TS^b^ (n=434), mean (SD)	USP^c^ (n=431), mean (SD)
Child Overall NHS and PSS cost^a^	865.84 (1455.68)	570.46 (1054.06)
OSI+TS intervention and supervision	282.53 (135.30)	—^d^
OSI+TS intervention cost	212.26 (90.06)	—
Supervision cost	70.27 (70.08)	—
Primary care cost	260.34 (648.28)	333.71 (847.54)
Secondary care cost	308.54 (976.92)	219.74 (522.83)
Medication cost	14.44 (53.89)	17.00 (53.66)
Child out-of-pocket	15.41 (61.87)	14.10 (59.82)
Travel cost for child health and PSS services	34.24 (81.68)	44.19 (139.97)
Child missed school	224.02 (1044.12)	262.69 (895.76)
School opportunity cost	24.84 (115.76)	29.12 (99.31)
Loss of future earnings	199.18 (928.36)	233.57 (796.45)
Parent NHS and PSS cost	897.36 (1996.86)	863.70 (1808.79)
Primary care cost	289.04 (595.70)	363.01 (837.33)
Secondary care cost	562.64 (1851.41)	465.89 (1507.77)
Medication cost	45.68 (97.51)	34.79 (75.90)
Parent out-of-pocket	34.75 (223.88)	22.16 (59.72)
Travel cost for parent health and PSS services	62.75 (196.13)	88.64 (305.49)
Parent opportunity cost of OSI+TS intervention	34.57 (16.30)	—
Parent missed work	74.59 (452.87)	69.14 (483.64)
Total societal cost		
Excluding missed school future earning loss	2044.35 (2899.34)	1701.51 (2555.07)
Including missed school future earning loss	2243.53 (3217.61)	1935.08 (2934.96)

a NHS and PSS: National Health Service and Personal Social Services.

b OSI+TS: online support and intervention for child anxiety with therapist support.

c USP: usual school practice.

d Not applicable.

**Table 5.** Mean difference in NHS and PSS and societal cost (in £) of children and parents^a^.

Cost	Unadjusted difference, mean (95% CI)		Unadjusted difference, *P* value	Adjusted difference^b^, mean (95% CI)		Adjusted difference^b^, *P* value
Child overall NHS and PSS^c^ cost	295.39 (95.37 to 495.40)		0.004	244.16 (76.26 to 412.07)		0.005
OSI+TS^d^ intervention and supervision	—^e^		—	—		—
OSI+TS intervention cost	—		—	—		—
Supervision cost	—		—	—		—
Primary care cost	−73.37 (−184.94 to 38.20)		0.19	−90.40 (−201.77 to 20.97)		0.11
Secondary care cost	88.80 (−46.48 to 224.07)		0.20	59.93 (−43.25 to 163.11)		0.25
Medication cost	−2.56 (−9.68 to 4.55)		0.48	−0.71 (−7.12 to 5.69)		0.83
Child out-of-pocket	1.31 (−7.62 to 10.24)		0.77	1.04 (−7.84 to 9.92)		0.82
Travel cost for child health and PSS services	−9.95 (−26.59 to 6.68)		0.24	−10.78 (−25.65 to 4.10)		0.15
Child missed school	−38.68 (−184.88 to 107.52)		0.60	−84.30 (-212.18 to 43.58)		0.19
School opportunity cost	-4.29 (-20.50 to 11.92)		0.60	−9.35 (−23.52 to 4.83)		0.19
Loss of future earnings	−34.39 (−164.38 to 95.60)		0.60	−74.95(−188.66 to 38.75)		0.19
Parent NHS and PSS cost	33.67 (−245.80 to 313.13)		0.81	29.79 (−223.52 to 283.10)		0.82
Primary care cost	−73.97 (−174.18 to 26.24)		0.15	-78.71 (−168.95 to 11.53)		0.09
Secondary care cost	96.75 (−152.42 to 345.92)		0.44	82.56 (−144.70 to 309.82)		0.47
Medication cost	10.89 (−1.67 to 23.45)		0.09	8.85 (−1.60 to 19.29)		0.095
Parent out-of-pocket	12.59 (−8.83 to 34.01)		0.25	13.19 (−5.99 to 32.37)		0.18
Travel cost for parent health and PSS services	−25.89 (−66.96 to 15.17)		0.21	−28.98 (−67.20 to 9.23)		0.14
Parent opportunity cost of OSI+TS intervention	—		—	—		—
Parent missed work	5.45 (−60.73 to 71.63)		0.87	−14.09 (−75.54 to 47.36)		0.65
Total societal cost						
Excluding missed school future earning loss	342.84 (−63.63 to 749.31)		0.097	313.90 (−53.57 to 681.38)		0.09
Including missed school future earning loss	308.45 (−152.22 to 769.12)		0.19	234.71 (−165.37 to 634.79)		0.25

a The confidence intervals of the unadjusted and adjusted differences are constructed based on the standard errors of an Ordinary Least Squares (OLS) regression clustered at the school level.

b In the column “Adjusted difference,” we adjusted for school batch, children’s gender, number of children in the school recruited in the trial, year group, index of multiple deprivation decile, free school meal status, and the baseline value of each measure.

c NHS and PSS: National Health Service and Personal Social Services.

d OSI+TS: online support and intervention for child anxiety with therapist support.

e Not applicable.

When accounting for sampling uncertainty, the CEAC for the CUA base-case analysis (Table 6 and Figure 1) shows that, in view of the joint distribution of incremental mean costs and effects (Figures 1 A and B), the probability that OSI+TS is cost-effective compared to USP only (Figures 1 A and B) ranges from 88.2% to 96.9% (UK adult value set for CHU9D) and 99.3% to 99.8% (Australia adolescent value set for CHU9D), respectively, at willingness to pay thresholds of £20,000 to £30,000 per extra QALY gained, respectively. The probabilities range from 94.2% to 98.1% (UK adult value set for CHU9D) and 99.7% to 99.9% (Australia adolescent value set for CHU9D), respectively, when the newly recommended threshold of £25,000 to £35,000 per QALY gained is considered (Table 7).

**Table 6.** Cost-effectiveness of OSI+TS^a^ compared to USP^b^ (CUA^c^ and CEA^d^ base case analyses).

Analyses^e^	Incremental^f^ cost (in £) mean (95% CI)	Incremental health outcomes mean (95% CI)	ICER^g^ (in £)	Probability cost-effective	
				at £20,000 WTP^h^	at £30,000 WTP
CUA-Base case 1^i^	244.16 (91.45 to 417.66)	0.021 (0.009 to 0.034)	11,462.47	0.882	0.969
CUA-Base case 2^j^	244.16 (91.45 to 417.66)	0.043 (0.020 to 0.063)	5700.98	0.993	0.998
CEA-Base case^k^	244.16 (91.45 to 417.66)	-0.032 (-0.065 to 0.021)	-7,736.13	—^l^	—

a OSI+TS: online support and intervention with therapist support.

b USP: usual school practice.

c CUA: cost-utility analysis.

d CEA: cost-effectiveness analysis.

e All these three analyses present incremental cost from the National Health Service (NHS) and Personal Social Services (PSS) perspective.

f Each incremental analysis is based on 200 bootstraps of 50 multiply imputed datasets. 95% CIs are constructed from the distribution of bootstrapped estimates using a percentile approach.

g ICER: Incremental Cost-Effectiveness Ratio.

h WTP: willingness-to-pay.

i CUA base case 1 presents incremental Quality-Adjusted Life Years (QALYs) derived from the Child Health Utility 9-Dimension (CHU9D) using the UK adult value set.

j CUA base case 2 presents incremental Quality-Adjusted Life Years (QALYs) derived from the Child Health Utility 9-Dimension (CHU9D) using the Australia adolescent value set.

k CEA base case presents the difference in the Anxiety Disorders Interview Schedule-Parent version (ADIS-P).

l Not applicable.

**Figure 1.**
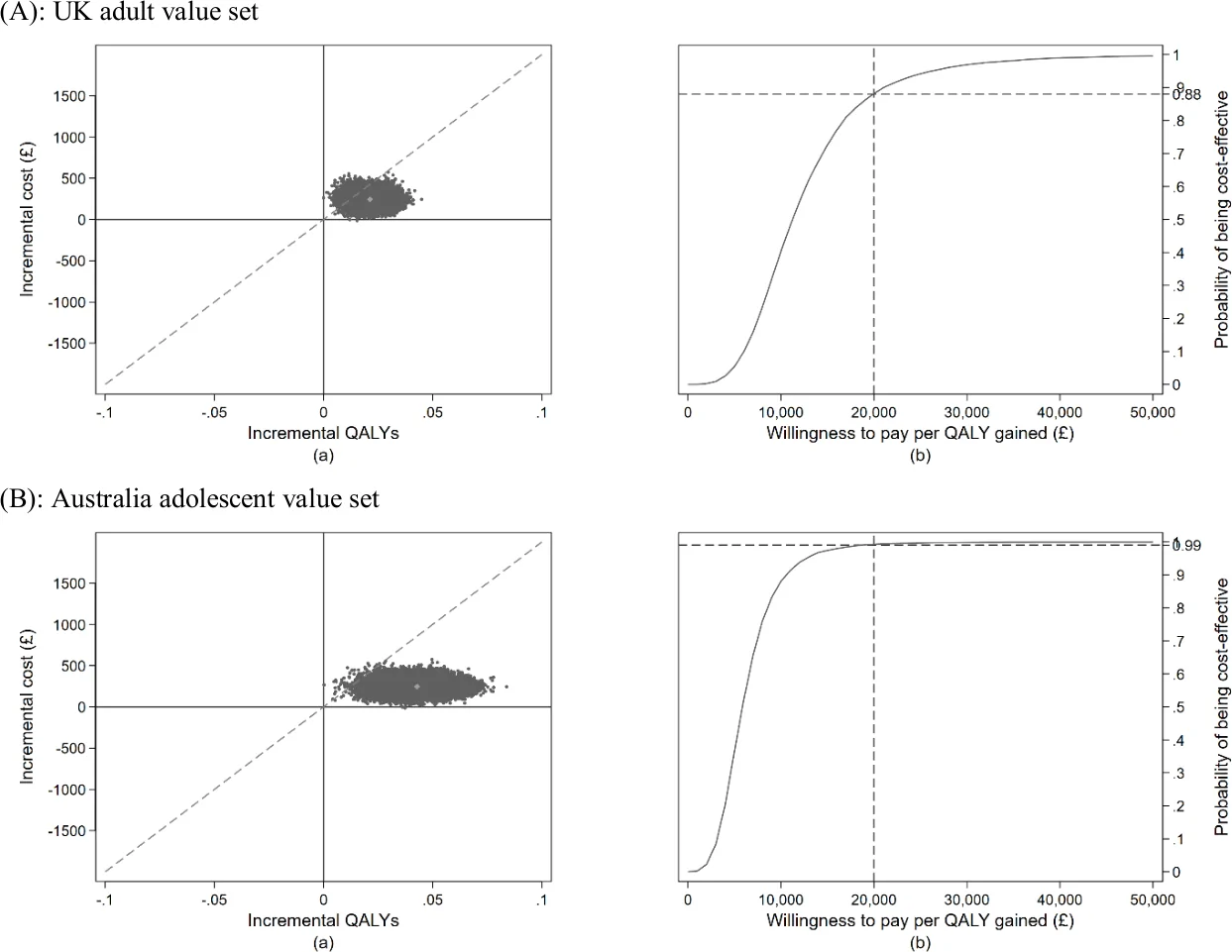
Cost-effectiveness planes (a) and Cost-effectiveness Acceptability Curves (b) for the cost-utility analysis Base Case analyses. This figure reports the cost-effectiveness planes and the cost-effectiveness acceptability curves of the base case analysis, which focuses on Child quality-adjusted life year (QALYs) and National Health Service and Personal Social Services costs. Child QALYs are derived using the UK adult value set in panel A and the Australian adolescent value set in panel B. Each cost-effectiveness plane (a) displays 10,000 incremental cost-QALY combinations, generated through 200 bootstrap iterations of 50 multiply imputed datasets.

**Table 7.** Cost-effectiveness of OSI+TS^a^ compared to USP^b^ (CUA^c^ base case analyses) at new cost-effectiveness thresholds

Analyses^d^	Incremental^e^ cost (in £) mean (95% CI)	Incremental health outcomes mean (95% CI)	ICER^f^ (in £)	Probability cost-effective	
				at £25,000 WTP^g^	at £35,000 WTP
CUA- Base case 1^h^	244.16 (91.45 to 417.66)	0.021 (0.009-0.034)	11,462.47	0.942	0.981
CUA- Base case 2^i^	244.16 (91.45 to 417.66)	0.043 (0.020-0.063)	5700.98	0.997	0.999

a OSI+TS: online support and intervention with therapist support.

b USP: usual school practice.

c CUA: cost-utility analysis.

d All these three analyses present incremental costs from the National Health Service (NHS) and Personal Social Services (PSS) perspective.

e Each incremental analysis is based on 200 bootstraps of 50 multiply imputed datasets. 95% CIs are constructed from the distribution of bootstrapped estimates using a percentile approach.

f ICER: Incremental Cost-Effectiveness Ratio.

g WTP: willingness-to-pay.

h CUA base case 1 presents incremental Quality-Adjusted Life Years (QALYs) derived from the Child Health Utility 9-Dimension (CHU9D) using the UK adult value set.

i CUA base case 2 presents incremental Quality-Adjusted Life Years (QALYs) derived from the Child Health Utility 9-Dimension (CHU9D) using the Australia adolescent value set.

Sensitivity analyses (Table S7 in Multimedia Appendix 1), including several versions of the societal perspective (SAs 1‐14), a per-protocol approach (SA 15‐16), a complete-case approach (SAs 17‐18), and a version adjusted for the three screening variables (SAs 19‐20), supported this finding with the probability of OSI+TS being a cost-effective alternative to USP only, ranging from 59.2% to 96.0% (UK adult value set for CHU9D) and 87.3% to 99.9% (Australia adolescent value set for CHU9D), respectively, at a WTP threshold of £20,000 per additional QALY. The corresponding probabilities at a £30,000 threshold ranged from 71.6% to 99.6% and 93.8% to 99.9%, respectively, for the two value sets underlying the CHU9D valuation. Results are also presented in terms of NMB and NHB (Table S8 in Multimedia Appendix 1). OSI+TS also remained cost-effective with the probability of 71% (UK adult value set for CHU9D) or 97.6 % (Australia adolescent value set for CHU9D) when a potential IT cost of up to £100 per-user for the OSI platform use was additionally added (Figure S1 in Multimedia Appendix 1).

Secondary analysis results from the CEA base-case analysis are presented in Table 6, and the corresponding CEAC in Figure S2 in Multimedia Appendix 1. The CEAC suggests that if the NHS is willing to pay more than approximately £11,000 to make one additional child free from anxiety disorders, the probability that OSI+TS is cost-effective, compared to USP, is greater than 50%. Nevertheless, the WTP threshold with respect to this clinical outcome is not established. Table S9 in Multimedia Appendix 1 presents sensitivity analyses for the CEA when we included societal cost or focused on per-protocol population.

## Discussion

### Principal Findings

To our knowledge, this is the first study to undertake an economic evaluation of a preventive digital intervention for anxiety problems in children, contributing to reduce the existing gap in the economic evidence-base. When both health-related quality of life and costs were jointly considered, the overall findings suggested that OSI+TS offered better value for money than USP. Therefore, our study indicated that OSI+TS is very likely to be cost-effective compared to USP for children aged 4‐7 years at risk of anxiety disorders. In the base-case CUA analysis (NHS and PSS perspective), the estimated ICER was £11,462.47 (£1=US $1.35 as of September 10, 2026) per QALY gained based on the UK adult value set, and £5,700.98 per QALY gained based on the Australia adolescent value set, both considerably below the £20,000‐30,000 WTP threshold recommended by the UK NICE [11], and even more so when the new £25,000 to 35,000 WTP threshold is considered [27]. Our results, therefore, provide evidence to advance potential implementation of OSI+TS. Twenty sensitivity analyses demonstrated that results were robust to accounting for societal costs and outcomes (ie, child-parent dyad QALYs), inclusion of cost of screening and a potential IT cost up to £100 per user, and to restricting analyses to the per-protocol population and complete cases. However, in two sensitivity analyses adopting a societal perspective and using the UK adult value set, the probability that OSI+TS is cost-effective fell to 59.2% at a WTP threshold of £20,000 per QALY, indicating considerable decision uncertainty. Although this represents the most conservative estimate of CE, given the recent increases in the NICE WTP threshold, these analyses suggest greater uncertainty than the NHS and PSS base-case analyses. This may have implications for decision-makers beyond NHS reimbursement bodies, such as families and education services, who may bear part of the intervention-related costs. The secondary CEA analysis indicates that decision makers should be willing to pay at least £11,000 to avoid one additional case of anxiety disorders to make the probability of OSI+TS being cost-effective compared to USP greater than 50%. However, the maximum threshold value that society is willing to pay for an additional child with no anxiety disorders has not been established, so we reported our results for a range of potential WTP thresholds that decision makers and society may consider. It is worth noting that the CEA and CUA evaluated different aspects of intervention benefit. The CEA assessed the presence of an anxiety disorder at 12 months, the CUA measured QALYs derived from the CHU9D, which captures broader HRQoL. It is, therefore, possible for an intervention to improve HRQoL without necessarily producing a statistically significant reduction in the proportion of children meeting diagnostic criteria for an anxiety disorder at a follow-up assessment.

The observed QALY gains were largely driven by improvements in HRQoL at 12 weeks, which were captured by the area-under-the-curve estimation of QALYs but had partially attenuated by the 12-month follow-up, when the primary clinical outcome was assessed. Furthermore, even if the primary clinical outcome was not statistically significant, all secondary clinical outcomes favored OSI+TS and showed statistically significant improvements [30]. Taken together, these findings suggest that the intervention produced broader improvements in children’s functioning and well-being, consistent with the HRQoL gains captured by the CHU9D. Nevertheless, given the attenuation of treatment effects over time and the methodological challenges associated with measuring preference-based HRQoL in younger children, the magnitude of these QALY gains should be interpreted with caution. Reimbursement agencies in the United Kingdom and many other countries around the world take their decisions based on CE analyses expressed in terms of cost per QALY gained. Therefore, it is mainly results from our cost-utility analyses, which provide compelling evidence of CE, that have the potential to shape practice and policy by informing policymakers’ decisions, conditional on some limitation that we discuss below.

We found that the cost of intervention is modest, ie mean of £282.53 (SD 135.30) per child, as the OSI online guided parent-self-help strategy required a modest amount of therapist time. Delivery of parent support sessions required a mean of 152 (SD 70) minutes, which is around 30 minutes less than the time we estimated, that is, a mean of 181.98 (SD 81), for delivering a similar version of OSI to 5 to 12 years old children with anxiety disorders in the context of UK CAMHS [17]. Furthermore, therapist delivery time in this study was half the time that therapists spent delivering treatment as usual, that is, a mean of 307.05 (SD 172.77) minutes in the UK CAMHS-based trial [17]. The therapists in this trial were well-trained and became highly experienced in the delivery of OSI+TS, as opposed to therapists in the United Kingdom CAMHS trial who each worked with a small number of families. The increased familiarity with OSI+TS is likely to have produced time efficiencies in delivery, as might intervening at an earlier stage in problem development. In line with recent recommendations [31], our findings suggest that this combination of a digital approach and prevention or early intervention can help services to better deal with an ever-increasing demand for child mental health support. All of these time savings translate into associated cost savings at times when healthcare budgets are under increasing financial pressure.

Our CE results are consistent with four other economic evaluations of preventive interventions for anxiety problems in children and young people [8]. However, our study is the first to evaluate a digital intervention for the prevention of childhood anxiety problems. The online delivery also brings potential to address barriers to access to child mental health care, including stigma and inability to travel to in-person sessions [6].

Strengths include the inclusion of sensitivity analyses taking a societal perspective, in recognition of the impact that child mental health problems have on the child’s family and beyond. We also estimated, and included in our sensitivity analyses, the human capital cost of school absenteeism (Section 4 in Multimedia Appendix 1), which has not been extensively considered in economic evaluations. Although researchers have highlighted the importance of accounting for children’s displaced time, properly evaluating its monetary value remains a challenge [32]. Our analyses adopted a lifetime-earning approach and determined the daily cost to be £275 in the English setting [33]. This substantial amount suggests that the cost of school absenteeism is nontrivial, and future research should also take account of it. In our trial, the cost of missed school was marginally higher, but not significantly so, in the USP arm, and children were young and mainly at risk of anxiety problems. However, if nascent problems are not promptly addressed, school absences may accumulate over time, and their adverse impact on educational achievements and future earnings may escalate [3]. Additionally, this study supports the application of targeted prevention approaches as an alternative to universal school-based mental health interventions [34], given recent suggestions that universal approaches could result in potential harms for some children and young people [35]. Our paper shows that providing targeted preventive intervention for children (identified as at risk by universal screening in schools) can improve their health-related quality of life and is a cost-effective strategy.

A number of important limitations need to be considered. First, schools and participants were enrolled in this study based on their consent, leading to potential nonrandom sample selection. Schools or families that were less focused on children’s mental well-being may have opted out of the study. Second, child anxiety problems are strongly related to school absence [36]. Hence, school-based screening risks excluding children not attending school. Third, the trial period was relatively short, ie, one year; the long-term effect of OSI+TS remains unknown. Therefore, future research should address this important evidence gap by extrapolating the results over the medium and longer term.

Fourth, some methodological considerations need to be acknowledged. A first methodological consideration concerns the use of the CHU9D in younger children. Current NICE guidance does not recommend any specific preference-based pediatric measure of health-related quality of life but recommends using a generic preference-based measure with good psychometric performance for the relevant age group [11,27]. Although the CHU9D was originally developed and validated for children aged 7 to 17 years, new emerging evidence supports its application in preschool and early school-aged children. In particular, studies in children aged 2 to 5 years have recently provided evidence of feasibility [37,38], construct validity, known-group validity, and responsiveness of the proxy CHU9D. Previously, another study showed feasibility and construct validity of the CHU9D in children aged 6 to 7 years [39]. Together, these findings support the use of the CHU9D descriptive system across the 4 to 7 years age range represented in this study. However, further validation work is warranted, and we therefore acknowledge this as a potential limitation in interpreting our findings.

A linked methodological issue concerns the valuation of CHU9D health states. Neither the UK adult nor the Australia adolescent value set was developed specifically for children younger than 7 years, and no consensus currently exists regarding the preferred value set for this age group. Nevertheless, recent evidence suggests that adult preferences for CHU9D health states are very similar when respondents are asked to value the health of a 2 to 4 years old child and a 10 years old child [40], supporting the application of a consistent valuation framework across childhood. In the absence of an age-specific value set and specific NICE preferences [27], we reported results using both the UK adult and the Australia adolescent value sets within the base-case analysis. This approach provides transparency regarding the impact of a value set choice and acknowledges the ongoing debate about preference weights for younger children. Both base-case analyses indicated that OSI+TS is cost-effective; however, the incremental cost per QALY gained differed depending on the value set applied, with ICERs of £11,462.47 and £5,700.98 using the UK adult and Australia adolescent value sets, respectively. For UK decision-making (but similar considerations may apply in other jurisdictions), decision-makers who prioritize the use of a value set derived from the local general population may prefer the UK adult value set, while those who wish to reflect preferences elicited from younger populations may favor the Australia adolescent value set. Ultimately, the choice of value set will depend on the perspective considered most appropriate for the decision context and the relevant national or local policy requirements.

Notwithstanding these limitations, our study provides much-needed economic evidence–and, to our knowledge, the first in terms of preventative digital interventions–that a preventive online intervention for children at risk of anxiety disorders is likely to be cost-effective compared to USP. Coupled with existing evidence of easier access, convenience, and acceptability of digital health interventions [6], the highly cost-effective OSI+TS appears to be a reliable technology to help address the alarming rise in child anxiety problems in the United Kingdom and beyond. Future research should explore the longer-term impacts. Meanwhile, efforts should be taken to integrate interventions such as this into broader strategies to enhance the mental health of at-risk children.

### Conclusion

This study evaluated the CE of a preventive digital intervention for childhood anxiety problems (OSI+TS) compared to USP alongside a cluster randomized trial conducted in 95 primary or infant schools in England for children aged 4‐7 at risk of anxiety problems. OSI+TS led to QALY gains of 0.021 (UK value set) and 0.043 (Australia value set), meaning that the child’s health-related quality of life improved compared to USP, with an additional cost of £244.16 from the NHS and PSS perspective. When improvements in health-related quality of life and differences in costs were considered together, the overall findings indicated that OSI+TS represented better value for money than USP. More specifically, this resulted in ICERs of £11,462.47 and £5,700.98 for the UK and Australia value sets, respectively, which are both below the £20,000 to £30,000 threshold recommended by NICE as per our prespecified Health Economics Analysis Plan. The probability of OSI being cost-effective at the threshold of £20,000 was 88% and 99% for these two value sets. When we considered the revised NICE thresholds, those probabilities increased to 94.2% and 99.7% for the two value sets at the threshold of £25,000. All sensitivity analyses, including those from a societal perspective, reinforced these findings, establishing OSI+TS as the first cost-effective digital early intervention for child anxiety problems, and supporting its future implementation to help reduce rising prevalence and long-term negative consequences.

## Supplementary material

10.2196/87123 Multimedia Appendix 1 Search strategies, Health Economics Analysis Plan, inclusion and exclusion criteria, cost of school absence, CONSORT flow diagram, CHEERS 2022 checklist, participant and school baseline characteristics, health economics data completeness, health outcomes, intervention resource use, child and parent service use, unit costs, and detailed cost-effectiveness analyses, including sensitivity analyses, net benefits, potential IT costs, and cost-effectiveness acceptability curves.

## Abbreviations

ADIS-P: Anxiety Disorders Interview Schedule for Children-Parent Interview
CAMHS: Child and Adolescent Mental Health Services
CBT: cognitive behavior therapy
CE: cost-effectiveness
CEA: cost-effectiveness analysis
CEAC: cost-effectiveness acceptability curve
CHU9D: Child Health Utility 9-Dimension
CONSORT: Consolidated Standards of Reporting Trials
CSRI: Client Service Receipt Inventory
CUA: cost-utility analysis
HEAP: Health Economics Analysis Plan
HRQoL: health-related quality of life
ICER: incremental cost-effectiveness ratio
IMD: Index of Multiple Deprivation
ISRCTN: International Standard Randomized Controlled Trial Number
ITT: intention-to-treat
MY-CATS: Minimising Young Children’s Anxiety through Schools
NHB: net health benefits
NHS: National Health Service
NICE: National Institute of Health and Care Excellence
NMB: net monetary benefit
OLS: Ordinary Least Squares
OSI+TS: online support and intervention for child anxiety with therapist support
PP: per-protocol
PSS: Personal Social Services
QALY: quality-adjusted life year
SA: sensitivity analyses
USP: usual school practice
WTP: willingness-to-pay
