# Supplementary material for: Estimating the Economic Value of Supported Online Parent-Led Cognitive Behavioral Therapy to Prevent Child Anxiety Problems: Economic Evaluation of the MYCATS Cluster Randomized Controlled Trial
Source: J Med Internet Res. 2026 Oct 8;28:e87123. doi: 10.2196/87123 (PMC13649241; doi:10.2196/87123)
Supplement: Supplementary file 1 [file jmir-v28-e87123-s001.doc]

**Section 1. Search Strategy**

| **OVID-Medline 1806 to present (24/06/24)** | | | |
| --- | --- | --- | --- |
| **Search Number** | **Search Terms** | | **Results** |
| 1 | digital treat*.ti,ab,kw. | | 175 |
| 2 | mobile applications/ | | 12,758 |
| 3 | exp internet/ | | 101,279 |
| 4 | exp computers, Handheld/ | | 14,134 |
| 5 | Therapy, Computer-Assisted/ | | 6,993 |
| 6 | (app or apps).ti,ab,kw. | | 48,305 |
| 7 | ((online or web or internet or digital*) adj3 (based or application* or intervention* or program* or therap*)).ti,ab,kw. | | 96,180 |
| 8 | (phone* or telephone* or smartphone* or cellphone* or smartwatch*).ti,ab,kw. | | 148,378 |
| 9 | (mobile health or mhealth or m-health or ehealth or e-health or emental or e-mental).ti,ab,kw. | | 26,010 |
| 10 | ((mobile health or mhealth or m-health or ehealth or e-health or emental or e-mental) adj3 (based or application* or intervention* or program* or therap*)).ti,ab,kw. | | 8,009 |
| 11 | (mobile* adj3 (based or application* or intervention* or device* or technolog*)).ti,ab,kw. | | 25,006 |
| 12 | medical informatics applications/ | | 2,552 |
| 13 | (online or web or internet or digital*).ti,ab,kw. | | 691,087 |
| 14 | 1 or 2 or 3 or 4 or 5 or 6 or 7 or 8 or 9 or 10 or 11 or 12 or 13 | | 908,368 |
| 15 | screening*.ti,ab,kw. | | 719,705 |
| 16 | preven*.ti,ab,kw. | | 1,852,941 |
| 17 | 15 or 16 | | 2,490,523 |
| 18 | child/ or child, preschool/ | | 2,211,274 |
| 19 | (child* or schoolchild or preschool or pre-school or kid? or preadolescen* or paediatric* or pediatric* or boy or boys or girl or girls or preteen* or pre-teen* or toddler? or minor?).ti,ab,kw. | | 2,341,610 |
| 20 | 18 or 19 | | 3,229,018 |
| 21 | exp anxiety disorders/ | | 94,259 |
| 22 | *Mental Disorders/ or Mental Health/ | | 203,743 |
| 23 | (anxiety or anxious or worri* or worry or panic or phobia? or emotion* symptom? or emotion* problem? or emotion* disorder? or emotion* difficult*).ti,ab,kw. | | 336,707 |
| 24 | ((mental* or psychiatr*) and (health or ill* or disorder?)).ti. | | 132,959 |
| 25 | ((internali?ing or internali?ed) adj3 (symptom? or problem? or disorder?)).ti,ab,kw. | | 9,314 |
| 26 | 21 or 22 or 23 or 24 or 25 | | 603,540 |
| 27 | economics/ | | 27,534 |
| 28 | exp "costs and cost analysis"/ | | 271,265 |
| 29 | exp Economics, Hospital/ | | 25,877 |
| 30 | exp Economics, Medical/ | | 14,443 |
| 31 | Economics, Nursing/ | | 4,013 |
| 32 | Economics, Pharmaceutical/ | | 3,139 |
| 33 | Budgets/ | | 11,824 |
| 34 | exp Models, Economic/ | | 16,374 |
| 35 | Markov Chains/ | | 16,225 |
| 36 | Monte Carlo Method/ | | 32,984 |
| 37 | Decision Trees/ | | 12,253 |
| 38 | econom$.tw. | | 436,591 |
| 39 | cba.tw. | | 11,394 |
| 40 | cea.tw. | | 27,953 |
| 41 | cua.tw. | | 1,508 |
| 42 | markov$.tw. | | 33,407 |
| 43 | (monte adj carlo).tw. | | 61,732 |
| 44 | (decision adj3 (tree$ or analys$)).tw. | | 32,517 |
| 45 | (cost or costs or costing$ or costly or costed).tw. | | 793,199 |
| 46 | (price$ or pricing$).tw. | | 56,668 |
| 47 | budget$.tw. | | 37,626 |
| 48 | expenditure$.tw. | | 71,912 |
| 49 | (value adj3 (money or monetary)).tw. | | 3,374 |
| 50 | (pharmacoeconomic$ or (pharmaco adj economic$)).tw. | | 4,635 |
| 51 | "Quality of Life"/ | | 289,935 |
| 52 | quality of life.tw. | | 404,404 |
| 53 | "Value of Life"/ | | 5,827 |
| 54 | Quality-Adjusted Life Years/ | | 16,521 |
| 55 | quality adjusted life.tw. | | 18,522 |
| 56 | (qaly$ or qald$ or qale$ or qtime$).tw. | | 15,491 |
| 57 | disability adjusted life.tw. | | 6,144 |
| 58 | daly$.tw. | | 5,513 |
| 59 | Health Status Indicators/ | | 24,127 |
| 60 | (sf36 or sf 36 or short form 36 or shortform 36 or sf thirtysix or sf thirty six or shortform thirtysix or shortform thirty six or short form thirtysix or short form thirty six).tw. | | 31,874 |
| 61 | (sf6 or sf 6 or short form 6 or shortform 6 or sf six or sfsix or shortform six or short form six).tw. | | 2,764 |
| 62 | (sf12 or sf 12 or short form 12 or shortform 12 or sf twelve or sftwelve or shortform twelve or short form twelve).tw. | | 8,079 |
| 63 | (sf12 or sf 12 or short form 12 or shortform 12 or sf twelve or sftwelve or shortform twelve or short form twelve).tw. | | 8,079 |
| 64 | (sf20 or sf 20 or short form 20 or shortform 20 or sf twenty or sftwenty or shortform twenty or short form twenty).tw. | | 467 |
| 65 | (euroqol or euro qol or eq5d or eq 5d).tw. | | 18,281 |
| 66 | (qol or hql or hqol or hrqol).tw. | | 78,911 |
| 67 | (hye or hyes).tw. | | 77 |
| 68 | health$ year$ equivalent$.tw. | | 40 |
| 69 | utilit$.tw. | | 287,218 |
| 70 | (hui or hui1 or hui2 or hui3).tw. | | 2,098 |
| 71 | disutili$.tw. | | 684 |
| 72 | rosser.tw. | | 111 |
| 73 | quality of wellbeing.tw. | | 52 |
| 74 | quality of well-being.tw. | | 507 |
| 75 | qwb.tw. | | 219 |
| 76 | willingness to pay.tw. | | 9,382 |
| 77 | standard gamble$.tw. | | 918 |
| 78 | time trade off.tw. | | 1,444 |
| 79 | time tradeoff.tw. | | 269 |
| 80 | tto.tw. | | 1,478 |
| 81 | chu9d.ti,ab,kw | | 86 |
| 82 | 27 or 28 or 29 or 30 or 31 or 32 or 33 or 34 or 35 or 36 or 37 or 38 or 39 or 40 or 41 or 42 or 43 or 44 or 45 or 46 or 47 or 48 or 49 or 50 or 51 or 52 or 53 or 54 or 55 or 56 or 57 or 58 or 59 or 60 or 61 or 62 or 63 or 64 or 65 or 66 or 67 or 68 or 69 or 70 or 71 or 72 or 73 or 74 or 75 or 76 or 77 or 78 or 79 or 80 or 81 | | 2,210,051 |
| 83 | 14 and 17 and 20 and 26 and 82 | | 323 |
|  |  | |  |
| **OVID-PsycINFO 1806 to present (24/06/24)** | | | |
| **Search Number** | **Search Terms** | | **Results** |
| 1 | mobile applications/ | | 3,065 |
| 2 | exp internet/ | | 34,339 |
| 3 | Computer-Assisted Therapy.ti,ab,hw. | | 1,299 |
| 4 | (app or apps).ti,ab,hw. | | 12,585 |
| 5 | ((online or web or internet or digital*) adj3 (based or application* or intervention* or program* or therap*)).ti,ab,hw. | | 41,682 |
| 6 | (phone* or telephone* or smartphone* or cellphone* or smartwatch*).ti,ab,hw. | | 84,885 |
| 7 | ((phone* or telephone* or smartphone* or cellphone* or smartwatch*) adj3 (based or application* or intervention* or program* or therap*)).ti,ab,hw. | | 6,995 |
| 8 | (mobile health or mhealth or m-health or ehealth or e-health or emental or e-mental).ti,ab,hw. | | 5,508 |
| 9 | ((mobile health or mhealth or m-health or ehealth or e-health or emental or e-mental) adj3 (based or application* or intervention* or program* or therap*)).ti,ab,hw. | | 2,027 |
| 10 | (mobile* adj3 (based or application* or intervention* or device* or technolog*)).ti,ab,hw. | | 12,840 |
| 11 | medical informatics.ti,ab,hw. | | 125 |
| 12 | (online or web or internet or digital*).ti,ab,hw. | | 247,015 |
| 13 | 1 or 2 or 3 or 4 or 5 or 6 or 7 or 8 or 9 or 10 or 11 or 12 | | 333,100 |
| 17 | preventive intervention.ti,ab,hw. | | 2,159 |
| 18 | exp Prevention/ or exp Preventive Health Services/ | | 78,881 |
| 19 | preventive treatment.ti,ab,hw. | | 684 |
| 20 | exp Screening/ or exp Anxiety Screening/ | | 20,029 |
| 21 | 17 or 18 or 19 or 20 | | 99,741 |
| 22 | child/ or child, preschool/ | | 0 |
| 23 | (child* or schoolchild or preschool or pre-school or kid? or preadolescen* or paediatric* or pediatric* or boy or boys or girl or girls or preteen or pre-teen* or toddler or minor).ti,ab,hw. | | 932,150 |
| 24 | 22 or 23 | | 932,150 |
| 25 | anxiety disorders/ or panic disorder/ or exp phobic disorders/ or *Mental Disorders/ or Mental Health/ | | 195,256 |
| 26 | (anxiety or anxious or worri* or worry or panic or phobia? or emotion* symptom? or emotion* problem? or emotion* disorder? or emotion* difficult*).ti,ab,hw. | | 297,590 |
| 27 | ((mental* or psychiatr*) and (health or ill* or disorder?)).ti. | | 116,190 |
| 28 | ((internali?ing or internali?ed) adj3 (symptom? or problem? or disorder?)).ti,ab,hw. | | 12,761 |
| 29 | 25 or 26 or 27 or 28 | | 483,597 |
| 30 | Economics/ | | 28,883 |
| 31 | exp "Costs and Cost Analysis"/ | | 51,313 |
| 32 | Budgets/ | | 1,500 |
| 33 | Markov Chains/ | | 1,994 |
| 34 | decision trees.ti,ab,hw. | | 728 |
| 35 | econom$.tw. | | 167,297 |
| 36 | cost-benefit analysis.tw. | | 1,244 |
| 37 | cost-utility analysis.tw. | | 427 |
| 38 | cost-effectiveness analysis.tw. | | 1,425 |
| 39 | markov$.tw. | | 5,491 |
| 40 | (monte adj carlo).tw. | | 5,724 |
| 41 | (decision adj3 (tree$ or analys$)).tw. | | 5,490 |
| 42 | (cost or costs or costing$ or costly or costed).tw. | | 125,821 |
| 43 | (price$ or pricing$).tw. | | 22,558 |
| 44 | budget$.tw. | | 10,636 |
| 45 | expenditure$.tw. | | 13,518 |
| 46 | (value adj3 (money or monetary)).tw. | | 1,326 |
| 47 | (pharmacoeconomic$ or (pharmaco adj economic$)).tw. | | 393 |
| 48 | "Quality of Life"/ | | 50,037 |
| 49 | quality adjusted life.tw. | | 1,850 |
| 50 | (qaly$ or qald$ or qale$ or qtime$).tw. | | 1,586 |
| 51 | disability adjusted life.tw. | | 590 |
| 52 | daly$.tw. | | 1,005 |
| 53 | (sf36 or sf 36 or short form 36 or shortform 36 or sf thirtysix or sf thirty six or shortform thirtysix or shortform thirty six or short form thirtysix or short form thirty six).tw. | | 6,192 |
| 54 | (sf6 or sf 6 or short form 6 or shortform 6 or sf six or sfsix or shortform six or short form six).tw. | | 92 |
| 55 | (sf12 or sf 12 or short form 12 or shortform 12 or sf twelve or sftwelve or shortform twelve or short form twelve).tw. | | 1,825 |
| 56 | (sf20 or sf 20 or short form 20 or shortform 20 or sf twenty or sftwenty or shortform twenty or short form twenty).tw. | | 69 |
| 57 | (euroqol or euro qol or eq5d or eq 5d).tw. | | 3,223 |
| 58 | (qol or hql or hqol or hrqol).tw. | | 19,508 |
| 59 | (hye or hyes).tw. | | 74 |
| 60 | health$ year$ equivalent$.tw. | | 5 |
| 61 | utilit$.tw. | | 75,445 |
| 62 | (hui or hui1 or hui2 or hui3).tw. | | 877 |
| 63 | disutili$.tw. | | 271 |
| 64 | rosser.tw. | | 94 |
| 65 | quality of wellbeing.tw. | | 33 |
| 66 | qwb.tw. | | 101 |
| 67 | willingness to pay.tw. | | 2,771 |
| 68 | standard gamble$.tw. | | 241 |
| 69 | time trade off.tw. | | 378 |
| 70 | time tradeoff.tw. | | 93 |
| 71 | tto.tw. | | 262 |
| 72 | 30 or 31 or 32 or 33 or 34 or 35 or 36 or 37 or 38 or 39 or 40 or 41 or 42 or 43 or 44 or 45 or 46 or 47 or 48 or 49 or 50 or 51 or 52 or 53 or 54 or 55 or 56 or 57 or 58 or 59 or 60 or 61 or 62 or 63 or 64 or 65 or 66 or 67 or 68 or 69 or 70 or 71 | | 457,150 |
| 73 | 16 and 21 and 24 and 29 and 72 | | 42 |
|  |  | |  |
| **EconLit-Proquest (up to 24/06/24)** | | | |
| **Search Number** | **Search Terms** | **Combined Terms** | **Results** |
| 1 | "digital interventions" |  |  |
| 2 | "digital health interventions" |  |  |
| 3 | internet-based* |  |  |
| 4 | "digital treatment" |  |  |
| 5 | "mobile applications" |  |  |
| 6 | "Computer-asssited therapy" |  |  |
| 7 | app |  |  |
| 8 | apps |  |  |
| 9 | ("online based" or "web based" or "digital application") |  |  |
| 10 | (phone* or telephone* or smartphone* or cellphone* or smartwatch*) |  |  |
| 11 | (**"**mobile health*" or mhealth* or m-health* or ehealth* or e-health* or emental or e-mental) |  |  |
| 12 | medical informatics applications |  |  |
| 13 | (online or web or internet or digital*) |  |  |
| 14 | 1 or 2 or 3 or 4 or 5 or 6 or 7 or 8 or 9 or 10 or 11 or 12 or 13 | ("digital interventions" or "digital health interventions" or internet-based* or "digital treatment" or "mobile applications" or "Computer-assited therapy" or app or apps or "online based" or "web based" or "digital application" or phone* or telephone* or smartphone* or cellphone* or smartwatch* or "mobile health*" or mhealth* or m-health* or ehealth* or e-health* or emental or e-mental or medical informatics applications or online or web or internet or digital*) | 45403 |
| 15 | (preventive interven* or preventive treat* or screening or preven*) | (preventive interven* or preventive treat* or screening or preven*) | 25586 |
| 16 | (child* or schoolchild or preschool or pre-school or kid? or preadolescen* or paediatric* or pediatric* or boy or boys or girl or girls or preteen* or pre-teen* or toddler? or minor?) | (child* or schoolchild or preschool or pre-school or kid? or preadolescen* or paediatric* or pediatric* or boy or boys or girl or girls or preteen* or pre-teen* or toddler? or minor?) | 54541 |
| 17 | noft(anxiety or anxious or worri* or worry or panic or phobia*) |  |  |
| 18 | TI((mental* or psychiatr*) and (health or ill* or disorder*)) |  |  |
| 19 | noft((internalising or internalised or internalizing or internalized) NEAR/3 (symptom* or problem* or disorder)) |  |  |
| 20 | 17 or 18 or 19 | noft(anxiety or anxious or worri* or worry or panic or phobia*) OR TI((mental* or psychiatr*) and (health or ill* or disorder*)) OR noft((internalising or internalised or internalizing or internalized) NEAR/3 (symptom* or problem* or disorder)) | 5814 |
| 21 | 14 AND 15 AND 16 AND 20 | (("digital interventions" or "digital health interventions" or internet-based* or "digital treatment" or "mobile applications" or "Computer-assited therapy" or app or apps or "online based" or "web based" or "digital application" or phone* or telephone* or smartphone* or cellphone* or smartwatch* or "mobile health*" or mhealth* or m-health* or ehealth* or e-health* or emental or e-mental or medical informatics applications or online or web or internet or digital*) ) AND (preventive interven* or preventive treat* or screening or preven*) AND (child* or schoolchild or preschool or pre-school or kid? or preadolescen* or paediatric* or pediatric* or boy or boys or girl or girls or preteen or pre-teen* or toddler or minor) AND (noft(anxiety or anxious or worri* or worry or panic or phobia*) OR TI((mental* or psychiatr*) and (health or ill* or disorder*)) OR noft((internalising or internalised or internalizing or internalized) NEAR/3 (symptom* or problem* or disorder))) | 3 |

**Section 2. Health Economic Analysis Plan**

**Minimising Young Children’s Anxiety through Schools (MY-CATS):**

**A Cluster Randomised Controlled Trial**

**Health Economics Analysis Plan**

**Health Economics Analysis Plan (HEAP) – Minimising Young Children’s Anxiety through Schools (MY-CATS): A Cluster Randomised Controlled Trial**

Essential items

|  |  | **Description** | **Study-specific description** |
| --- | --- | --- | --- |
| **Section 1: Administrative information** | | | |
| 1.1 | Title | Title that matches protocol and which includes the phrase ‘Health Economics Analysis Plan' | Health economics analysis plan for Minimising Young Children’s Anxiety through Schools (MY-CATS): A Cluster Randomised Controlled Trial |
| 1.2 | Trial registration number | Trial registration number and name of registry that uniquely identifies the clinical trial on a publicly-accessible registry (and other relevant trial study numbers) | ISRCTN 82398107. Prospectively registered on 14.1.2021. https://www.isrctn.com/ISRCTN82398107 |
| 1.3 | Source of funding | Name of funders for trial and economic evaluation and funder(s)’ reference number(s) | Kavli Trust – Programme on Health Research |
| 1.4 | Purpose of HEAP | Brief statement of the purpose of the HEAP | The purpose of this HEAP is to describe the analysis and reporting procedure intended for the economic analyses to be undertaken. The analysis plan is designed to ensure that there is no conflict with the protocol and associated statistical analysis plan and it should be read in conjunction with them. |
| 1.5 | Trial protocol version | Trial protocol version number associated with this HEAP | This document has been written based on information contained in the trial protocol version 2.3, dated 6 December 2022. |
| 1.6 | Trial Statistical Analysis Plan (SAP) version | SAP version number associated with this HEAP | SAP Version: 1.0, Date: 15/12/2021 |
| 1.7 | Trial HEAP version | Sequential number and date of this version | HEAP Version: 1.1, Date: 27/07/2023 |
| 1.8 | HEAP revisions | Date, justification for revision and summary of changes to the HEAP. Specify the individual making any revisions/changes to the HEAP. | The following revisions were made by Mara Violato:  *Sections 1.5* *and 2.6*– updated details (i.e. version and date) of trial protocol.  *Sections 1.7 –* updated details (i.e. version and date) of HEAP.  *Section 2.7* - Trial start and end dates updated.  *Section 4.1* – “Stata 17” was replaced with “Stata 18”, because a new version of the Stata software is now available.  *Section 4.7* – the reason why two valuation sets are used for the CHU-9D instrument in the cost-utility analysis was missing – it has now been added.  *Section 5.14* – the use of two valuation sets for the CHU-9D instrument was double-reported in the HEAP version 1.0. As it had already been accounted for in section 4.7 as part of the primary analyses, it has now been dropped from the sensitivity analyses section 5.14. |
| 1.9 | Roles and responsibilities | Names, affiliations and roles of individuals who have significantly contributed to the HEAP | This HEAP was prepared by Assoc Prof Mara Violato (senior health economist), Health Economics Research Centre, Nuffield Department of Population Health, University of Oxford. The trial junior (to be appointed) and senior (Mara Violato) health economists are responsible for conducting and reporting the economic evaluation in accordance with the HEAP. |
| 1.10a | Signature(s) of person(s) writing HEAP | Signature(s) of the person(s) writing the HEAP (and date) |   Date: 27/07/2023 |
| 1.10b | Signature of senior health economist | Signature of senior health economist who is guarantor of the economic evaluation (and date) |   Date: 27/07/2023 |
| 1.10c | Signature of Chief Investigator | Signature of the Chief Investigator for the trial (and date) |   Date: 27/07/2023 |
| **Section 2: Trial introduction & background** | | | |
| 2.1 | Trial background and rationale | Synopsis of trial background and rationale including a brief description of research question and brief justification for undertaking the trial | Half of all lifetime cases of anxiety begin by age 11 (1), and the estimated prevalence among children and adolescents worldwide is 6.5% (2). Child anxiety disorders have a negative impact on children, their families, and the society at large (3, 4). It is therefore important to intervene before anxiety disorders develop.  My-CATS is a parallel group, superiority cluster randomised controlled trial (RCT), which sets out to test the effectiveness and cost-effectiveness of the provision of a new online parent-led intervention (OSI - Online Support and Intervention for child anxiety) with therapist support in addition to usual school practice (from hereon ‘OSI’ means ‘OSI + usual school practice’) compared with usual school practice only for children aged 4-7. OSI is targeted at children at risk of developing anxiety disorders, as identified in the school context through systematic screening. |
| 2.2 | Aim(s) of the trial | Clearly and briefly state the main aim(s) of the trial | Briefly, the MY-CATS trial aims to determine whether providing OSI to at-risk children identified through systematic screening in schools brings benefits over and above usual school practice. The study also sets out to identify the characteristics of children who do and do not benefit from the intervention and the mechanisms of change from the intervention. |
| 2.3 | Objectives and/or research hypotheses of the trial | Describe specific trial objectives (primary and secondary) or trial hypotheses | Primary objective: to compare diagnostic outcomes 12-months post-randomisation for children who screen positive on one or more risk factors in schools allocated to intervention versus usual school practice. The trial hypothesis is that fewer children will have an anxiety disorder in the intervention arm compared to the usual school practice arm at 12-months.  Secondary objectives are to: (i) compare anxiety symptoms, related interference, externalising symptoms, and additional intervention targets at 12 weeks and 12 months post-randomisation amongst children who screen positive on one or more risk factors in schools allocated to the intervention arm, compared to those in schools allocated to usual school practice; (ii) examine potential moderators and mediators of the primary outcome; (iii) evaluate experiences of systematic screening and the supported parent-led online intervention and (iv) estimate the cost-effectiveness of the intervention compared to usual school practice, 12 months post-randomisation. |
| 2.4 | Trial population | Describe the trial inclusion and exclusion criteria | Inclusion criteria for screening:   - Child (aged 4-7) is in a participating class (Reception, Year 1, Year 2). - One parent/carer provides written consent and completes screening questionnaires for the child.   Inclusion criteria for the trial:   - Child screens positive on the basis of at least one of the following screening questionnaires: child anxiety symptoms (score ≥34 on the Preschool Anxiety Scale; PAS), behavioural inhibition (score ≥30 on the Approach subscale of the Short Temperament Scale for Children; STSC-A), parent/carer anxiety symptoms (score ≥8 on the Generalised Anxiety Disorder-7 Scale). - One parent/carer provides written consent and agrees to complete all assessments. - A maximum of one child per family/household will be eligible to take part in the trial. Where more than one child in a family/household screens positive on the basis of at least one screening questionnaire, the child with the highest score on the PAS will be eligible. If two or more children in the family/household have equal PAS scores, the child with the highest STSC-A score will be eligible.   Exclusion criteria for the trial:   - Parent/carer does not have sufficient use of English to provide consent, complete measures, and/or take part in the intervention. - Parent/carer does not have frequent access to the internet, either at home or elsewhere. |
| 2.5 | Intervention(s) and comparator(s) | Describe the intervention(s) and comparator(s) | Intervention: Parents/carers of children in schools allocated to the intervention arm will be offered a brief, parent-led online intervention (OSI: Online Support and Intervention for child anxiety) (5) in addition to usual school practice. OSI is an online version of an evidence-based parent-guided treatment for child anxiety disorders (6, 7), and the content has been adapted for parents/carers of children (aged 4-7) at risk of developing anxiety disorders. OSI includes seven online modules for parents, supported by weekly short telephone sessions (approximately 20 minutes) with a Children’s Wellbeing Practitioner (CWP), and a follow-up review telephone session 4 weeks later.  Comparator: Usual school practice – there is not a standard school practice, so the latter is likely to vary by school. Families in both arms will be free to continue to seek and access social, emotional, and mental health support and intervention for their child, and schools in both arms will continue to provide any usual social, emotional and mental health support offered to children and families in their school. After the 12-month assessment, parents/carers in the usual school practice arm will be offered written versions of the content of the OSI website. |
| 2.6 | Trial design | Briefly describe the trial design including type of trial such as cluster, crossover, etc. Can also include details of power calculation, sample size (including any separate calculations for economic endpoints), randomisation and blinding. | This is a parallel group, superiority cluster randomised controlled trial, with schools (clusters) randomised to the intervention or usual school practice arm in a 1:1 ratio stratified according to level of deprivation within the school and balanced on cluster size. The study will aim to recruit and randomise 86 primary/infant schools in England, and on the basis of recruiting 86 schools, approximately 876 trial participants will be recruited. This target sample size has been revised with respect to trial protocol version 2.3, dated 6 December 2022, based on evidence on recruitment and screening rates obtained from recruitment of school batches one and two. |
| 2.7 | Trial start and end dates | Trial recruitment start and end dates and the follow-up period | School recruitment started in February 2021 and ended in June2022. Child screening in the recruited schools was conducted in 5 batches, from March 2021 to July 2022. The follow-up period will run for 12 months until September 2023, when the follow-up for participants belonging to the fifth batch of schools will be completed. |
| **Section 3: Economic approach/overview** | | | |
| 3.1 | Aim(s) of economic evaluation | Describe the aim(s) of the economic evaluation | The aim of the economic evaluation is to address the question “What is the cost-effectiveness of OSI for children identified at risk for anxiety disorders compared to ‘Usual School Practice’?” |
| 3.2 | Objective(s) of economic evaluation | Describe the objectives (primary and secondary) of the economic evaluation | The primary objective of the health economic evaluation is to estimate the cost-effectiveness of OSI for children identified at risk for anxiety disorders compared to ‘Usual School Practice’, 12 months post-randomisation, in a within-trial economic evaluation. |
| 3.3 | Overview of economic analysis | Briefly outline and justify the type of economic evaluation to be undertaken, identifying the primary economic analysis and outlining the analysis plan and the methods that will be used | The within-trial economic analysis will be performed using individual participant (pupil) level data from the MY-CATS trial. The analytical approaches will take the form of a cost-utility analysis (CUA) and a cost-effectiveness analysis (CEA). Based on trial evidence, incremental cost-effectiveness ratios will be calculated by taking a ratio of the difference in the mean costs (numerator) and mean utility /effect (denominator) in the CUA and CEA, respectively. |
| 3.4 | Jurisdiction(s) | Specify the jurisdiction(s) in which the analysis will be conducted including details of the country(s) and health system(s) | The trial is conducted in the UK, which has a national health service (NHS), providing publicly funded healthcare, primarily free of charge at the point of use. The type of support that OSI offers may be then provided to children not only through the NHS but also through Local Authorities. |
| 3.5 | Perspective(s) | State the perspective(s) from which the economic analysis is being conducted, such as societal perspective and/or healthcare payer perspective | The primary economic analyses (CUA and CEA) will be from the NHS and personal social services (PSS) perspective in the base-case analyses. A sensitivity analysis for both will include a societal perspective. |
| 3.6 | Time horizon(s) | State the time horizon(s) over which costs and consequences are being evaluated | The economic analyses will compare the costs and consequences of each trial arm at 12 months post-randomisation. |
| **Section 4: Economic data collection & management** | | | |
| 4.1 | Statistical software | Specify the statistical software that will be used to carry out the health economic analysis | Stata version 18.0 or higher (StataCorp LP; College Station, TX) will be used for conducting the economic analysis. |
| 4.2 | Identification of resources | Justify and describe items of resource use that will be measured as part of the trial | The following items of health care resource use and broader resources that may differ between trial arms will be measured during the study period: primary and secondary health care and social care resource use for both the child and the parent/carer; medication for both the child and the parent/carer; travel time/cost associated with accessing those resources; time off school for the child; time off work and associated productivity losses for the parent/carer; opportunity cost for the parent/carer associated with them using OSI (i.e. time spent online on OSI and time spent on support calls from therapists). In addition, therapist’s (Children Wellbeing Practitioner - CWP) time spent in training/supervision, administrative tasks, and delivery of the intervention, and supervisor’s time spent training/supervising the CWPs will be measured to assess the amount of resources and cost of the intervention. Time spent by the teachers/school staff in screening-related activities will also be captured (opportunity cost of teachers’ /school staff’s time), as well as resources used in ‘usual school practice’. |
| 4.3 | Measurement of resource-use data | Describe the resource-use data collection method(s) (including external routine datasets) and the time points at which they will be used. | Child and parent/carer resource use data will be collected online and measured using a modified version of the Client Services Receipt Inventory (CSRI) (8), which will be completed by the parent/carer at baseline, 12-weeks and 12-months post-randomisation. At baseline and 12-week assessments, parents will also be provided with a diary to keep a record of time off school/work and use of services throughout the study duration so to facilitate completing subsequent CSRIs.  To identify and measure resources used in the intervention and screening, we will use “ad hoc” designed therapist (CWP), supervisor and school staff logs.  Measuring resources used in ‘usual school practice’ is challenged by the fact that there exists no standard school practice and/or standard measures available across schools. Therefore, the clinical team have developed some questions to assess ‘usual practice’ in schools. Schools nominate a member of staff to answer these questions, either online or via phone call at baseline and 12 months. Questions are open-ended and based on those, a coding frame is then developed to summarise responses and provide descriptive information on what ‘usual practice’ looks like in each school. We do expect the type and extent of support provided in schools may vary. In addition to this, we also try to capture ‘usual school practice’ through the ‘ad hoc’ modified CSRI, above mentioned. The CSRI in fact includes a specific section on community and school resources used that the parent/carer is required to complete at each assessment point. |
| 4.4 | Valuation of resource-use data | For each resource item measured, describe how the unit cost will be derived and from which specific price year. Outline how adjustments will be made for sources from different price years and which inflation index will be used. | All resource use will be valued in monetary terms using appropriate UK unit costs derived from local and national sources and/or participant’s valuations estimated at the time of the study (2021-2023). Costs will be expressed in pounds sterling at 2022/2023 prices. Adjustments will be made for inflation, when necessary, using the NHS cost inflation index (NHSCII) for health professionals / health care services and the ONS Retail Price Index for other resources (9). Unit costs for primary and social care and other community services will be obtained from the PSSRU publications (9). Unit NHS reference costs will be employed to value hospital resource use, e.g. A&E visits, outpatient and inpatient attendances (10). Medication costs will be taken from the British National Formulary (BNF) (11) and the Prescription Cost Analysis (PCA) for England (12). Time off school for children will be costed as a minimum as ‘opportunity cost’ for the educational sector (6, 13) using values from relevant governmental sources (e.g. department of education school spent per pupil), and acknowledging the limitations of the approach. If new published literature emerges, which reports on valuations of the cost of school absence for the child‘s future prospects, those valuations will be used to capture more comprehensively the cost of school absence for the child. Time off work for parent/ carer will be costed using the Annual Survey of Hours and Earnings (ASHE) (14), and teacher’s/school staff’s time will be costed using data from the National Association of Schoolmasters Union of Women Teachers (15) and/or other relevant organisations. |
| 4.5 | Identification of outcome(s) | Specify and justify the outcome(s) that will be measured | The primary economic outcome measures will be child’s Quality-Adjusted Life Years (QALYs) derived from utility scores, obtained using the CHU-9D (parent-report on child) quality of life instrument (16-18) in the CUA; and child’s absence/presence of an anxiety disorder at 12-months post-randomisation established on the basis of the ADIS-P, in the CEA.  Parent/carer Quality-Adjusted Life Years (QALYs) derived from utility scores, obtained using the EQ-5D-5L quality of life instrument (19, 20) will be calculated for both trial arms. Parent–child dyad QALYs will be obtained by additively combining individual parent and child QALYs (21) and used as the outcome in a cost-utility sensitivity analysis from the societal perspective. Potential limitation of this approach will be discussed (22). |
| 4.6 | Measurement of outcome(s) | Describe the outcome data collection method(s) and the time points at which they will be used | Outcome data will be collected online at baseline, and at 12 weeks and 12 months post randomisation, and via diagnostic interviews with parents/carers at 12 months. |
| 4.7 | Valuation of outcome(s) | For each outcome measured, describe how it will be valued and the source of these valuations | Utility scores for the child will be derived from responses to the CHU-9D parent-report on child, using both the preference weights obtained from a sample of the UK adult general population (17) and preferences weights obtained from Australian adolescents aged 11 to 17 years (23), as no established guideline exists as to which value set is more appropriate.  Parent utility scores will be derived from responses to the EQ-5D-5L. UK utility values will be derived using the approach recommended by NICE, which is to use a validated mapping function from the existing EQ-5D-3L (19, 24, 25). Utility score will be used to generate child and parent QALYS over the duration of the trial (from baseline to 12 months follow-up). |
| **Section 5: Economic data analysis** | | | |
| 5.1 | Analysis population | Outline the analysis population that will be used in the economic base-case analysis (such as intention to treat, per protocol) | The base-case analysis of both child outcomes (QALYs and absence/presence of an anxiety disorder at 12-months post-randomisation) will be conducted according to the Intention-To-Treat (ITT) principle, with participants analysed according to the trial arm their school was randomised to. |
| 5.2 | Timing of analyses | Describe the timing of all planned analyses (e.g. interim and final analyses) | The final analysis (within-trial analysis) will be conducted once all patients have been followed for 12 months post-randomisation. |
| 5.3 | Discount rates for costs and benefits | Detail the source of, and justification for, discount rates used for costs and benefits | Given the short time-frame of the trial and follow-up, discounting will not be applied to costs or effects. |
| 5.4 | Cost-effectiveness threshold(s) | Detail the cost-effectiveness threshold(s) to be used in analysis/interpretation | In the CUA, a cost-effectiveness threshold of £20,000 -£30,000 per QALY will be use, as per NICE guidelines (19). For the CEA, the maximum threshold value that society is willing to pay for an extra child free from anxiety disorders is unknown. |
| 5.5 | Statistical decision rule(s) | Describe how inference will be drawn (e.g. significance level, confidence intervals or mean net benefit) | Mean differences in costs and effects (QALYs and presence/absence of anxiety disorders) will be estimated with associated 95% confidence intervals. |
| 5.6 | Analysis of resource use | Describe how differences in the use of resources/services between randomised groups will be compared | Mean differences in the use of services between randomised groups will be described and compared statistically, stratified by type of resource use. |
| 5.7 | Analysis of costs | Describe analyses of the cost data, specifying any covariates for statistical adjustment, assumptions, and alternative methods | Unadjusted and adjusted (for baseline costs) differences in overall mean costs between the arms will be analysed initially using Ordinary Least Squares (OLS) regression. The distribution of residuals from the regression model will then be examined and a decision will be made as to whether OLS is appropriate or another type of regression model should be considered (e.g. Generalised Linear Models (GLM). Analyses will be adjusted for clustering. Other covariates for adjustment will also be considered in discussion with the statisticians in order to align the statistical and economic analyses as much as possible. These may include: school-level free school meal status (the stratification factor) and cluster size as a continuous variable to reflect its role in the randomisation process; gender; year group; and the decile of the index of multiple deprivation rank (as a continuous predictor) as a measure of socioeconomic status. |
| 5.8 | Analysis of outcomes | For each outcome used in the economic analysis, describe how the outcome will be analysed, specifying any covariates for statistical adjustment, assumptions, and alternative methods | Unadjusted and adjusted (for baseline utility in the CUA, and baseline presence/absence of anxiety in the CEA) mean differences in outcomes will be analyse using an appropriate regression model (e.g. OLS, LPM, GLM). Analyses will be adjusted for clustering. Other covariates for adjustment will also be considered in discussion with the statisticians in order to align the statistical and economic analyses as much as possible. These may include: school-level free school meal status (the stratification factor) and cluster size as a continuous variable to reflect its role in the randomisation process; gender; year group; and the decile of the index of multiple deprivation rank (as a continuous predictor) as a measure of socioeconomic status. |
| 5.9 | Data cleaning for analysis | Outline how data will be cleaned before analysis | Descriptive statistics will be used to identify potential mistakes (e.g. typos at the data entry level). Suspected mistakes will be reported to the trial manager who will check the data against the source documents/master data. Corrections identified will be documented in the Stata code. |
| 5.10 | Missing data | Specify the procedure for dealing with missing data | Trial data will be examined for any missing data. Mean imputation methods will be used for missing resource use deemed highly deterministic (e.g. telephone/online therapist contact), and multiple imputation for other resources (e.g. use of medications), under the assumption of missing at random (26). The specification of the imputation model will be considered in discussion with the statisticians in order to align the statistical and economic analyses as much as possible. |
| 5.11 | Analysis of cost-effectiveness | Describe the methods that will be used to summarise cost-effectiveness. | Cost and QALY data will be combined to calculate an incremental cost-effectiveness ratio (ICER) from both the NHS & PSS perspective and a societal perspective. Seemingly Unrelated Regression (SUR) will be used, if appropriate, to account for the correlation between the costs and the effects. Analyses will be adjusted for clustering. |
| 5.12 | Sampling uncertainty | Describe how uncertainty around the costs and effectiveness estimates and summary cost-effectiveness measures will be explored | Uncertainty in the cost-effectiveness results will be analysed by use of cost-effectiveness acceptability curves (27) over a range of potential threshold values that the health system might be willing to pay for an additional QALY gained, in the CUA. Cost-effectiveness acceptability curves will be used also in the CEA, although the maximum threshold value that society is willing to pay for an extra child free from anxiety disorders is unknown. |
| 5.13 | Subgroup analyses or analysis of heterogeneity | Describe any analyses of subgroups or heterogeneity in cost-effectiveness and the analysis methods used | N/A |
| 5.14 | Sensitivity analyses | Describe any sensitivity analyses and their form | Several sensitivity analyses will be undertaken to explore uncertainties surrounding key parameters in the economic evaluation. These will include: taking a societal perspective for both the CUA and the CEA where the outcomes refer to the child only; NHS and societal perspectives in the CUA, where the outcomes are parent–child dyad QALYs; conducting base-cases analyses on complete cases only. Other sensitivity analyses may be appropriate/required once the data have been made available. |
| **Section 6: Modelling** | | | |
| 6.1 | Extrapolation or decision analytic modelling | Outline whether decision analytic modelling or any other extrapolation will be used to estimate cost-effectiveness results beyond the period of the trial or to introduce an additional comparator or other evidence. | N/A |
| 6.2 | Model type | Describe the modelling approach that will be used and duration of extrapolation | N/A |
| 6.3 | Model structure | Detail the model structure (where possible, include diagram of model states and transitions between them) | N/A |
| 6.4 | Treatment effect beyond the end of the trial | Describe the duration and size of treatment effect in the period beyond the end of the trial | N/A |
| 6.5 | Other key assumptions | List the key structural assumptions of the model | N/A |
| 6.6 | Methods for identifying and estimating parameters | For each model parameter, describe the methods and data sources that will be used to estimate the parameter (e.g. from the RCT, systematic review, meta-analysis, other published data or expert opinion) | N/A |
| 6.7 | Model uncertainty | Describe the methods that will be used to assess parameter uncertainty in the results. Describe sensitivity analyses for the impact of other types of uncertainty on results. | N/A |
| 6.8 | Model validation | Describe the methods and data that will be used to check the face, internal and external validity of the model | N/A |
| 6.9 | Subgroup analyses/heterogeneity | Describe subgroup or heterogeneity analyses that will be executed and reported within the extrapolation or decision analytic modelling | N/A |
| **Section 7: Reporting/publishing** | | | |
| 7.1 | Reporting standards | Describe any guidelines that will be followed when publishing results | CHEERS guidelines (28) will be followed when reporting the health economic evaluation. |
| 7.2 | Deviations from the HEAP | Describe the procedure for reporting any deviations from the HEAP | Any deviation from HEAP will be described and justified in the final published report. |
| **Section 8: Appendices** | | | |
| 8.1 | Health economic collection tools | Include template examples of the resource-use data collection sheets and resource-use questionnaires | Data will be collected online, but the paper version of each questionnaire used, including preference-based measured (CHU-9D and EQ-5D) will be included in the Appendices. |

Optional items

|  |  | **Description** | **Example** |
| --- | --- | --- | --- |
| **Section 1: Administrative information** | | | |
| O1.1 | Table of contents | List of HEAP contents with page numbers | N/A |
| O1.2 | Abbreviations/glossary of terms/definitions | List of abbreviations and/or acronyms used within the HEAP alongside their meanings/definitions | CEA: cost-utility analysis.  CHU-9D: Child Health Utility 9 Dimension instrument  CSRI: Client Service Receipt Inventory  CUA: cost-effectiveness analysis  EQ-5D-5L: EuroQol 5 Dimension 5 Level instrument  NHS: National Health Service  PSS: personal and social services  QALY: quality-adjusted life year |
| **Section 4: Economic data collection & management** | | | |
| O4.1 | Monitoring collection of health economic data | Outline how the health economic data collected will be monitored | The health economics questionnaires will be administered online using REDCap (Research Electronic Data Capture) databases, therapist/supervisor/school staff logs will be collected using excel files, and OSI usage data will be collected within the OSI online platform, and exported as excel files. The trial health economist(s) will work closely with the trial team throughout the data collection period. Data collection forms will be assessed throughout the trial period to monitor quality of the data and amend any forms or procedures if necessary. |
| O4.2 | Database management | Outline how the economic data will be stored and managed and by whom | Economic data will be securely stored on the trial database and managed by the trial database manager, Tessa Reardon. |
| O4.3 | Data entry | Outline how data will be entered/handled and outline any checking systems in place | All the health economics questionnaire data will be captured online. The database will use controls to limit data entry to plausible values.  Individual therapist/supervisor/school staff logs will be completed using excel files. The study team will manually check logs for potential errors and merge data from individual logs into a single database.  Diagnostic outcome data will be entered manually. Data entered manually (e.g. diagnostic interview outcome data), will be checked by a second researcher.  OSI usage data exports will be regularly checked by the team to identify potential errors. |
| O4.4 | Data archiving | State whether datasets, interim datasets and final analysis will be archived, and if so, how | A copy of health economic analysis files, derived datasets, interim datasets and final analysis will be locked and archived. Archived datasets will be held by the University of Oxford and will conform to the University data security policy and data compliance and Data Protection Act policies. The study team will develop plans to make a version of the de-identified dataset (together with detailed procedure documents, data dictionaries and analysis files) that is suitable for sharing available via a suitable repository, and the original final de-identified datasets will be retained on the University server. |
| **Section 6: Modelling** | | | |
| O6.1 | Value of information analysis | Describe whether value of information analysis is planned and the type and methods that will be used to calculate value of information | N/A |
| **Section 8: Appendices** | | | |
| O8.1 | Cross-referencing to other trial documents | Reference to other relevant trial documents that are adhered to and followed when writing the HEAP and any other references used when writing the HEAP | N/A |
| O8.2 | Illustrations | Illustrations such as annotated questionnaires detailing the database fieldnames, flow charts outlining the flow of data for the economic evaluation, or template tables | N/A |

**References**

**Section 3. Inclusion and Exclusion Criteria**

Inclusion criteria for screening

- Children should be aged 4-7 years and enrolled in a participating class (i.e. Reception, Year 1, or Year 2).
- The parent or caregiver of children has provided written consent and completed screening questionnaires for the child.

Inclusion criteria for the trial

- Children are screened positive based on at least one of the following screening questionnaires: child anxiety symptoms (score ≥ 34 on the Preschool Anxiety Scale; PAS), behavioural inhibition (score ≥ 30 on the Approach subscale of the Short Temperament Scale for Children; STSC-A), parent/carer anxiety symptoms (score ≥ 8 on the Generalised Anxiety Disorder-7 Scale).
- The parent or caregiver of children has provided written consent.
- Only one child per family or household may participate in the trial. If more than one child screens positive based on any screening questionnaire, the child with the highest PAS score is eligible. If two or more children have equal PAS scores, the child with the highest STSC-A score will be selected.

Exclusion Criteria for the trial

- The parent or caregiver does not have adequate proficiency in English to provide consent, complete measures, or participate in the intervention.
- The parent or caregiver does not have frequent internet access at home or elsewhere.

**Section 4. Cost of School Absence**

We considered the cost implications of school absences from two points of view when evaluating the societal cost of childhood anxiety problems. First, when assessing the societal cost, previous literature has primarily focused on the unrealised pre-paid educational expenditure. Besides that, school absence can also be a cost in terms human capital loss, which has not been widely considered in previous economic evaluations.

We incorporated both aspects in our calculations. The pre-paid educational expenditure was obtained by calculating the UK national per-pupil funding per school day in the UK.^1^ We obtained the unit cost by dividing the 2021/22 UK national per pupil (£6,510 at 2021/22 prices) by the standard 190 school days in the UK. To estimate the daily human capital loss associated with anxiety-related school absenteeism, we leveraged a model from Psacharopoulos et al. (2021).^2^

In this model, the human capital loss from one year of school absence, L, is represented by:

L = PV (Y × a × r),

where PV (·) is the present value function, *Y* is the average annual earning, *a* is the fraction of a school year that someone missed, and *r* is the return of one year of schooling. We calibrated this model in the context of our study by searching for England's specific values in 2021/22. We used the UK median gross annual earnings, £27,774 (2022 price),^3^ available from the official statistics. To calculate the daily missed school cost loss, we set *a* to 1/190 to reflect 190 school days. In line with Psacharopoulos et al. (2021), we used an 8% return rate for education and assumed that the average British worker earns income over a 45-year career, with future earnings discounted at a rate of 3.5%. This resulted in an estimated human capital loss of £274.76 for each missed school day.

1.	Department for Education. School funding statistics, Financial year 2023-24 [Internet]. School funding statistics, Financial year 2023-24. 2024 [cited 2024 Aug 20]. Available from: https://explore-education-statistics.service.gov.uk/find-statistics/school-funding-statistics

2.	Psacharopoulos G, Collis V, Patrinos HA, Vegas E. The COVID-19 Cost of School Closures in Earnings and Income across the World. Comparative Education Review. 2021 May;65(2):271–87.

3.	Office for National Statistics. Earnings and working hours [Internet]. Earnings and working hours. [cited 2024 Aug 20]. Available from: https://www.ons.gov.uk/employmentandlabourmarket/peopleinwork/earningsandworkinghours

**Section 5. CONSORT Flow Diagram**

K=number of schools. N=number of children. FU=follow-up. Where participants did not complete a follow-up, they were invited to complete subsequent follow-ups unless they withdrew from the trial. 13 intervention arm participants and 4 usual school practice participants withdrew before completing the 6-week follow-up; 0 intervention arm participants and 2 usual school practice participants withdrew after the 6-week follow-up and before completing the 12-week follow-up.

***Source***: Reardon T, Ukoumunne OC, Dodd H, Halliday G, Hill C, Jasper B, Jones B, Lawrence PJ, Morgan F, Placzek A, Rapee RM, Violato M, Yu S, Team M, Creswell C. Parent-led CBT delivered via online and telephone support alongside usual school practice versus usual school practice only for young children identified as at risk for anxiety disorders through screening in schools: a cluster randomised controlled trial. Journal of Child Psychology and Psychiatry 2026;67(8):1280–1296. doi: 10.1111/jcpp.70119

**Section 6. CHEERS 2022 Checklist**

| **Topic** | **No.** | **Item** | **Location where item is reported** |
| --- | --- | --- | --- |
| **Title** |  |  |  |
|  | 1 | Identify the study as an economic evaluation and specify the interventions being compared. | Title, page 1 |
| **Abstract** |  |  |  |
|  | 2 | Provide a structured summary that highlights context, key methods, results, and alternative analyses. | Abstract, pages 2-3 |
| **Introduction** |  |  |  |
| **Background and objectives** | 3 | Give the context for the study, the study question, and its practical relevance for decision making in policy or practice. | Pages 4-6 |
| **Methods** |  |  |  |
| **Health economic analysis plan** | 4 | Indicate whether a health economic analysis plan was developed and where available. | Page 6 & 12 & Supplementary Material S2 |
| **Study population** | 5 | Describe characteristics of the study population (such as age range, demographics, socioeconomic, or clinical characteristics). | Page 6 |
| **Setting and location** | 6 | Provide relevant contextual information that may influence findings. | Page 6 |
| **Comparators** | 7 | Describe the interventions or strategies being compared and why chosen. | Pages 7-8 |
| **Perspective** | 8 | State the perspective(s) adopted by the study and why chosen. | Pages 9 & 10 |
| **Time horizon** | 9 | State the time horizon for the study and why appropriate. | Pages 6 & 10 |
| **Discount rate** | 10 | Report the discount rate(s) and reason chosen. | Page 10 |
| **Selection of outcomes** | 11 | Describe what outcomes were used as the measure(s) of benefit(s) and harm(s). | Pages 8-9 |
| **Measurement of outcomes** | 12 | Describe how outcomes used to capture benefit(s) and harm(s) were measured. | Pages 8-10 |
| **Valuation of outcomes** | 13 | Describe the population and methods used to measure and value outcomes. | Pages 8-10 |
| **Measurement and valuation of resources and costs** | 14 | Describe how costs were valued. | Pages 9-10 |
| **Currency, price date, and conversion** | 15 | Report the dates of the estimated resource quantities and unit costs, plus the currency and year of conversion. | Page 10 |
| **Rationale and description of model** | 16 | If modelling is used, describe in detail and why used. Report if the model is publicly available and where it can be accessed. | N/A |
| **Analytics and assumptions** | 17 | Describe any methods for analysing or statistically transforming data, any extrapolation methods, and approaches for validating any model used. | Pages 10-12 |
| **Characterising heterogeneity** | 18 | Describe any methods used for estimating how the results of the study vary for subgroups. | N/A |
| **Characterising distributional effects** | 19 | Describe how impacts are distributed across different individuals or adjustments made to reflect priority populations. | N/A |
| **Characterising uncertainty** | 20 | Describe methods to characterise any sources of uncertainty in the analysis. | Pages 11-12 |
| **Approach to engagement with patients and others affected by the study** | 21 | Describe any approaches to engage patients or service recipients, the general public, communities, or stakeholders (such as clinicians or payers) in the design of the study. | Notes to Supplementary Table S2, Page 45 |
| **Results** |  |  |  |
| **Study parameters** | 22 | Report all analytic inputs (such as values, ranges, references) including uncertainty or distributional assumptions. | N/A |
| **Summary of main results** | 23 | Report the mean values for the main categories of costs and outcomes of interest and summarise them in the most appropriate overall measure. | Pages 12-21 |
| **Effect of uncertainty** | 24 | Describe how uncertainty about analytic judgments, inputs, or projections affect findings. Report the effect of choice of discount rate and time horizon, if applicable. | Pages 18-21 |
| **Effect of engagement with patients and others affected by the study** | 25 | Report on any difference patient/service recipient, general public, community, or stakeholder involvement made to the approach or findings of the study | Notes to Supplementary Table S2, Page 45 |
| **Discussion** |  |  |  |
| **Study findings, limitations, generalisability, and current knowledge** | 26 | Report key findings, limitations, ethical or equity considerations not captured, and how these could affect patients, policy, or practice. | Pages 21-27 |
| **Other relevant information** |  |  |  |
| **Source of funding** | 27 | Describe how the study was funded and any role of the funder in the identification, design, conduct, and reporting of the analysis | Page 28 |
| **Conflicts of interest** | 28 | Report authors conflicts of interest according to journal or International Committee of Medical Journal Editors requirements. | Pages 28-29 |

*From:* Husereau D, Drummond M, Augustovski F, et al. Consolidated Health Economic Evaluation Reporting Standards 2022 (CHEERS 2022) Explanation and Elaboration: A Report of the ISPOR CHEERS II Good Practices

**Table S1.1. Baseline Characteristics of Participants**

|  | **OSI+TS (N=434)** | **USP (N=431)** | **Total (N=865)** |
| --- | --- | --- | --- |
| Batch |  |  |  |
| Cohort 1 | 51 (12%) | 58 (13%) | 109 (13%) |
| Cohort 2 | 126 (29%) | 125 (29%) | 251 (29%) |
| Cohort 3 | 118 (27%) | 118 (27%) | 236 (27%) |
| Cohort 4 | 99 (23%) | 118 (27%) | 217 (25%) |
| Cohort 5 | 40 (9%) | 12 (3%) | 52 (6%) |
| Child year group^*^ | | | |
| Reception, 4-5 years | 124 (29%) | 130 (30%) | 254 (29%) |
| Year 1, 5-6 years | 152 (35%) | 145 (34%) | 297 (34%) |
| Year 2, 6-7 years | 158 (37%) | 156 (36%) | 314 (36%) |
| Child age, years^†^ | 6∙3 (0∙9) | 6∙2 (0∙9) | 6∙2 (0∙9) |
| Child gender | | | |
| Female | 219 (50%) | 213 (49%) | 432 (50%) |
| Male | 215 (50%) | 218 (51%) | 433 (50%) |
| Other | 0 | 0 | 0 |
| Child ethnicity^**^ | | | |
| Asian^††^ | 17/434 (4%) | 11/430 (3%) | 28/864 (3%) |
| Black^⸸^ | 4/434 (<1%) | 5/430 (1%) | 9/864 (1%) |
| Mixed^¥^ | 35/434 (8%) | 34/430 (8%) | 69/864 (8%) |
| White^‡^ | 373/434 (86%) | 378/430 (88%) | 751/864 (87%) |
| Other^#^ | 4/434 (<1%) | 0/430 (0%) | 4/864 (<1%) |
| Prefer not to say | 1/434(<1%) | 2/430 (<1%) | 3/864 (<1%) |
| Parent age, years^‡‡^ | 36∙7 (5∙6) | 37∙1 (5∙4) | 36∙9 (5∙5) |
| Parent gender | | | |
| Female | 409 (94%) | 401 (93%) | 810 (94%) |
| Male | 25 (6%) | 29 (7%) | 54 (6%) |
| Other | 0 | 1 (<1%) | 1 (<1%) |
| Parent relationship to child | | | |
| Mother | 407 (94%) | 396 (92%) | 803 (93%) |
| Father | 25 (6%) | 29 (7%) | 54 (6%) |
| Other | 2 (<1%) | 6 (1%) | 8 (1%) |
| Parent ethnicity^**^ | | | |
| Asian^††^ | 19/430 (4%) | 14/428 (3%) | 33/858 (4%) |
| Black^⸸^ | 6/430 (1%) | 6/428 (1%) | 12/858 (1%) |
| Mixed^¥^ | 14/430 (3%) | 11/428 (3%) | 25/858 (3%) |
| White^‡^ | 383/430 (89%) | 392/428 (92%) | 775/858 (90%) |
| Other^#^ | 6/430 (1%) | 3/428 (1%) | 9/858 (1%) |
| Prefer not to say | 2/430 (<1%) | 2/428 (<1%) | 4/858 (<1%) |
| Parent is partnered | 361 (83%) | 353 (82%) | 714 (83%) |
| Parent cohabits with partner^⸸⸸^ | 346/361 (96%) | 343/353 (97%) | 689/714 (96%) |
| Type of housing^**^ | | | |
| Privately rented | 71/431 (16%) | 81/427 (19%) | 152/858 (18%) |
| Council or housing association rented | 72/431 (17%) | 62/427 (15%) | 134/858 (16%) |
| Mortgage/owned | 283/431 (66%) | 277/427 (65%) | 560/858 (65%) |
| Other | 2/431 (<1%) | 6/427 (1%) | 8/858 (1%) |
| Prefer not to say | 3/431 (1%) | 1/427 (<1%) | 4/858 (1%) |
| Parent education | | | |
| School completion | 22 (5%) | 30 (7%) | 52 (6%) |
| Further education | 148 (34%) | 145 (34%) | 293 (34%) |
| Higher education | 139 (32%) | 134 (31%) | 273 (32%) |
| Postgraduate education | 113 (26%) | 113 (26%) | 226 (26%) |
| None of the above | 5 (1%) | 4 (1%) | 9 (1%) |
| Prefer not to say | 7 (2%) | 5 (1%) | 12 (1%) |
| Parent employment^**^ |  |  |  |
| Unemployed | 35/432 (8%) | 27/431 (6%) | 62/863 (7%) |
| Part-time | 166/432 (38%) | 173/431 (40%) | 339/863 (39%) |
| Full time | 145/432 (34%) | 144/431 (33%) | 289/863 (33%) |
| Student | 12/432 (3%) | 7 /431 (2%) | 19/863 (2%) |
| Retired | 0/432 (0%) | 1/431 (<1%) | 1/863 (<1%) |
| Homemaker | 54/432 (13%) | 51/431 (12%) | 105/863 (12%) |
| Other | 16/432 (4%) | 22/431 (5%) | 38/863 (4%) |
| Prefer not to say | 4/432 (1%) | 6/431 (1%) | 10//863 (1%) |

**Table S1.1. Baseline Characteristics of Participants (Continued)**

|  | **OSI+TS (N=434)** | **USP (N=431)** | **Total (N=865)** |
| --- | --- | --- | --- |
| Household annual income^**^ | | | |
| < £16,000 | 62/419 (15%) | 59/425 (14%) | 121/844 (14%) |
| £16,001-£30,000 | 50/419 (12%) | 56/425 (13%) | 106/844 (13%) |
| £30,001-£40,000 | 38/419 (9%) | 46/425 (11%) | 84/844 (10%) |
| £40,001-£50,000 | 44/419 (11%) | 44/425 (10%) | 88/844 (10%) |
| £50,001-£60,000 | 47/419 (11%) | 49/425 (12%) | 96/844 (11%) |
| £60,001-£70,000 | 42/419 (10%) | 29/425 (7%) | 71/844 (8%) |
| £70,001-£80,000 | 29/419 (7%) | 28/425 (7%) | 57/844 (7%) |
| £80,001-£90,000 | 20/419 (5%) | 29/425 (7%) | 49/844 (6%) |
| £90,001-120,000 | 22/419 (5%) | 26/425 (6%) | 48/844 (6%) |
| > £120,000 | 21/419 (5%) | 19/425 (4%) | 40/844 (5%) |
| Prefer not to say | 44/419 (11%) | 40 /425 (9%) | 84/844 (10%) |
| Index of Multiple Deprivation decile^¥¥^ | | | |
| 1^st^ | 42 (10%) | 42 (10%) | 84 (10%) |
| 2^nd^ | 40 (9%) | 21 (5%) | 61 (7%) |
| 3^rd^ | 62 (14%) | 47 (11%) | 109 (13%) |
| 4^th^ | 39 (9%) | 29 (7%) | 68 (8%) |
| 5^th^ | 39 (9%) | 71 (16%) | 110 (13%) |
| 6^th^ | 51 (12%) | 43 (10%) | 94 (11%) |
| 7^th^ | 36 (8%) | 49 (11%) | 85 (10%) |
| 8^th^ | 50 (12%) | 31 (7%) | 81 (9%) |
| 9^th^ | 44 (10%) | 60 (14%) | 104 (12%) |
| 10^th^ | 31 (7%) | 38 (9%) | 69 (8%) |
| Screening outcome | | | |
| One risk | 247 (57%) | 209 (48%) | 456 (53%) |
| Two risks | 149 (34%) | 165 (38%) | 314 (36%) |
| Three risks | 38 (9%) | 57 (13%) | 95 (11%) |
| Screened positive for child anxiety | 297 (68%) | 337 (78%) | 634 (73%) |
| Screened positive for behavioural inhibition | 126 (29%) | 132 (31%) | 258 (30%) |
| Screened positive for parent anxiety | 236 (54%) | 241 (56%) | 477 (55%) |
| *Notes:* Data are n (%) or mean (SD) or n/N(%), unless otherwise stated. Data were available for all randomised participants unless otherwise stated. OSI+TS= Online Support and Intervention for Child Anxiety with therapist support. USP=usual school practice. This table is adapted from Table 2 of the pre-print paper (https://papers.ssrn.com/sol3/papers.cfm?abstract_id=5027298), which examines the clinical results of the same trial (MYCATS).*Year group at screening  †Child age at randomisation  **Data were not available for all randomised participants.  ††Includes Asian or Asian British Indian, Pakistani, any other Asian background  ⸸Includes Black or Black British African, Caribbean, any other Black background  ¥Includes White and Black Caribbean, White and Black African, White and Asian, any other mixed background  ‡Includes British, Irish, any other White background  #Includes Chinese  ‡‡Data were available for 381 in the Intervention arm and 385 in the Usual school practice arm  ##Data were available for 430 in the Intervention arm and 428 in the Usual school practice arm  ⸸⸸Only includes those who are partnered  ¥¥ Based on family postcode: 1 = least deprived; 10=most deprived | | | |

**Table S1.2. Baseline Characteristics of Schools**

|  | **OSI+TS (N=434)** | **USP (N=431)** | **Total (N=865)** |
| --- | --- | --- | --- |
| Cohort |  |  |  |
| Cohort 1 | 6 (13%) | 7 (15%) | 13 (14%) |
| Cohort 2 | 12 (25%) | 10 (21%) | 22 (23%) |
| Cohort 3 | 12 (25%) | 13 (28%) | 25 (26%) |
| Cohort 4 | 13 (27%) | 14 (30%) | 27 (28%) |
| Cohort 5 | 5 (10%) | 3 (6%) | 8 (8%) |
| Region in England |  |  |  |
| North-West / North-East | 11 (23%) | 9 (19%) | 20 (21%) |
| West-Midlands / east-Midlands | 16 (33%) | 14 (30%) | 30 (32%) |
| Eastern | 6 (12∙5%) | 5 (11%) | 11 (12%) |
| South-West | 6 (12∙5%) | 5 (11%) | 11 (12%) |
| Greater London and South East | 7 (15%) | 11 (23%) | 18 (19%) |
| Yorkshire and Humber | 2 (4%) | 3 (6%) | 5 (5%) |
| Size |  |  |  |
| Number of pupils on school roll | 421 (154) | 462 (147) | 441 (151) |
| Number of eligible classes, median (range) | 6 (6 to 12) | 6 (6 to 15) | 6 (6 to 15) |
| Deprivation |  |  |  |
| Above national median of pupils eligible for free school meals^*^ | 23 (48%) | 22 (47%) | 45 (47%) |
| Percentage of pupils eligible for free school meals | 17∙4 (11∙8) | 17∙9 (10∙3) | 17∙6 (11∙0) |
| Language |  |  |  |
| Above national median of pupils with English as an additional language^†^ | 13 (27∙1) | 16 (34∙0) | 29 (31∙0) |
| Percentage of pupils with English as an additional pupils language | 17∙7 (20∙3) | 22∙1 (23∙4) | 19∙9 (21∙9) |
| Special Educational Needs |  |  |  |
| Percentage of pupils on Special Educational Needs Support | 13∙7 (7∙8) | 14∙2 (6∙1) | 13∙9 (7∙0) |
| Percentage of pupils with an Education Health Care Plan | 1∙2 (0∙9) | 1∙3 (0∙9) | 1∙2 (0∙9) |
| *Notes:* Data are n (%) or mean (SD), unless otherwise stated. OSI+TS= Online Support and Intervention for Child Anxiety with therapist support. USP=usual school practice. This table is adapted from Table 1 of the pre-print paper (https://papers.ssrn.com/sol3/papers.cfm?abstract_id=5027298), which examines the clinical results of the same trial (MYCATS).  *National median for primary schools in England in 2019 was 15∙8%  †National median for primary schools in England in 2019 was 21∙2% | | | |

**Table S2. Health Economics Data Completeness (%)**

|  | **OSI+TS (N=434)** | **USP (N=431)** | **Total (N=865)** |
| --- | --- | --- | --- |
| Health Outcomes |  |  |  |
| Child CHU9D - baseline | 100.00 | 100.00 | 100.00 |
| Child CHU9D - 12 week | 80.18 | 83.53 | 81.85 |
| Child CHU9D - 12 month | 71.66 | 73.32 | 72.49 |
| Parent EQ5D - baseline | 100.00 | 100.00 | 100.00 |
| Parent EQ5D - 12 week | 79.72 | 83.53 | 81.62 |
| Parent EQ5D - 12 month | 70.74 | 72.62 | 71.68 |
| Child Anxiety Disorders - 12 month* | 71.43 | 72.39 | 71.91 |
|  |  |  |  |
| Service and Medication Use |  |  |  |
| Child - baseline | 100.00 | 100.00 | 100.00 |
| Child - 12 week | 79.72 | 83.29 | 81.50 |
| Child - 12 month | 70.74 | 72.62 | 71.68 |
| Parent - baseline | 100.00 | 100.00 | 100.00 |
| Parent - 12 week | 79.72 | 83.29 | 81.50 |
| Parent - 12 month | 70.74 | 72.62 | 71.68 |
|  |  |  |  |
| Absenteeism |  |  |  |
| School absence - baseline | 100.00 | 100.00 | 100.00 |
| School absence - 12 week | 79.72 | 83.29 | 81.50 |
| School absence - 12 month | 70.74 | 72.62 | 71.68 |
| Work absence - baseline | 100.00 | 100.00 | 100.00 |
| Work absence - 12 week | 79.72 | 83.29 | 81.50 |
| Work absence - 12 month | 70.74 | 72.62 | 71.68 |
|  |  |  |  |
| Intervention and Supervision Logs |  |  |  |
| Treatment logs | 93.78 | NA | NA |
| Supervision logs | 87.56 | NA | NA |
| *Notes:* Percentages calculated with respect to 434 children in the OSI+TS arm and 431 children in the control arm. OSI+TS= Online Support and Intervention for Child Anxiety with therapist support. USP=usual school practice. Patient and Public Involvement and Engagement in adapting the CSRI questionnaire helped to simplify questions and may have contributed to reduce missing data.  *Child anxiety disorders was derived from the ADIS-P outcome. | | | |

**Table S3. Mean and Mean Difference in Health Outcomes for Child and Parent – Complete Case Analysis**

|  | **OSI+TS** | | | **USP** | | | **Unadjusted difference** | | | **Adjusted difference** | | |
| --- | --- | --- | --- | --- | --- | --- | --- | --- | --- | --- | --- | --- |
|  | **N** | **Mean** | **SD** | **N** | **Mean** | **SD** | **Mean** | **95% CI** | **P-value** | **Mean** | **95% CI** | **P-value** |
| **Child CHU9D - UK value set:** |  |  |  |  |  |  |  |  |  |  |  |  |
| Baseline | 434 | 0.856 | 0.091 | 431 | 0.841 | 0.102 | 0.016 | (-0.000,0.032) | 0.057 |  |  |  |
| 12 weeks | 348 | 0.886 | 0.096 | 360 | 0.857 | 0.102 | 0.029 | (0.012,0.046) | 0.001 | 0.022 | (0.009,0.035) | <0.0001 |
| 12 months | 311 | 0.886 | 0.099 | 316 | 0.873 | 0.103 | 0.013 | (-0.002,0.029) | 0.095 | 0.009 | (-0.004,0.021) | 0.167 |
| Total Child QALYs | 294 | 1.001 | 0.113 | 304 | 0.973 | 0.117 | 0.028 | (0.006,0.049) | 0.014 | 0.028 | (0.015,0.041) | <0.0001 |
| **Child CHU9D - AUS value set:** |  |  |  |  |  |  |  |  |  |  |  |  |
| Baseline | 434 | 0.715 | 0.198 | 431 | 0.687 | 0.214 | 0.028 | (-0.006,0.062) | 0.106 |  |  |  |
| 12 weeks | 348 | 0.783 | 0.199 | 360 | 0.718 | 0.218 | 0.065 | (0.029,0.101) | 0.001 | 0.053 | (0.025,0.081) | <0.0001 |
| 12 months | 311 | 0.778 | 0.208 | 316 | 0.749 | 0.217 | 0.030 | (-0.002,0.062) | 0.070 | 0.021 | (-0.004,0.047) | 0.103 |
| Total Child QALYs | 294 | 0.879 | 0.201 | 304 | 0.824 | 0.212 | 0.055 | (0.018,0.093) | 0.004 | 0.050 | (0.027,0.074) | <0.0001 |
| **Parent EQ-5D-5L** |  |  |  |  |  |  |  |  |  |  |  |  |
| Baseline | 434 | 0.781 | 0.214 | 431 | 0.788 | 0.205 | -0.008 | (-0.041,0.026) | 0.656 |  |  |  |
| 12 weeks | 346 | 0.811 | 0.214 | 360 | 0.798 | 0.227 | 0.013 | (-0.025,0.051) | 0.507 | 0.019 | (-0.001,0.039) | 0.063 |
| 12 months | 307 | 0.807 | 0.233 | 313 | 0.827 | 0.186 | -0.019 | (-0.055,0.016) | 0.281 | -0.007 | (-0.025,0.010) | 0.401 |
| Total Parent QALYs | 290 | 0.913 | 0.244 | 302 | 0.919 | 0.225 | -0.006 | (-0.050,0.038) | 0.776 | 0.014 | (-0.004,0.032) | 0.128 |
| **Child anxiety disorder status outcome - ADIS-P** |  |  |  |  |  |  |  |  |  |  |  |  |
| 12 months | 310 | 0.068 | 0.252 | 312 | 0.115 | 0.320 | -0.048 | (-0.102,0.007) | 0.084 | -0.038 | (-0.080,0.003) | 0.070 |
| *Notes:* In the column "Adjusted difference", we adjusted for the baseline value of each measure and for the following variables: school batch, children's gender, number of children in the school recruited in the trial, year group, index of multiple deprivation decile, free school meal status. The confidence intervals of the unadjusted and adjusted differences were constructed based on the standard errors clustered at the school level. CHU9D= Child Health Utility 9 Dimensions instrument. EQ-5D-5L = EuroQoL-5 Dimension 5-level instrument. QALY=quality-adjusted life year. ADIS-P= Anxiety Disorder Interview Schedule for Children - Parent Interview. OSI+TS= Online Support and Intervention for Child Anxiety with therapist support. USP=usual school practice. | | | | | | | | | | | | |

**Table S4: Summary of Therapist and Supervisor time (minutes) Spent on Intervention and Supervision for OSI+TS Group – Complete Case analyses**

|  | **N** | **Mean** | **SD** | **Median** | **IQR** |
| --- | --- | --- | --- | --- | --- |
| Intervention-related time | 407 | 306.06 | 127.09 | 330 | (247,388) |
| treatment time (delivery) | 407 | 152.33 | 69.68 | 165 | (124,200) |
| preparation | 407 | 56.63 | 24.63 | 61 | (40,75) |
| post-session activities | 407 | 81.34 | 39.21 | 82 | (54,112) |
| additional contact | 407 | 15.76 | 23.42 | 10 | (0,20) |
| Supervision-related time | 380 | 78.00 | 75.11 | 62 | (35,103) |
| case time (supervisor) | 380 | 30.64 | 26.89 | 25 | (15,41) |
| case time (therapist) | 380 | 30.64 | 26.89 | 25 | (15,41) |
| preparation time (supervisor) | 380 | 4.73 | 8.61 | 2 | (1,5) |
| preparation time (therapist) | 380 | 10.15 | 12.81 | 7 | (4,13) |
| post-session activities (supervisor) | 380 | 0.88 | 6.67 | 0 | (0,0) |
| post-session activities (therapist) | 380 | 0.95 | 4.78 | 0 | (0,0) |
| *Note:* This table summarises therapists’ and supervisors’ time spent on each participant in the intervention arm. This summary is based on the participants who are recorded at least once in the treatment and supervision logs. OSI+TS= Online Support and Intervention for Child Anxiety with therapist support. | | | | | |

**Table S5.1: Children’s Service Use at Baseline – Complete Case Analysis**

|  | **Group** | | | | | | | | | |
| --- | --- | --- | --- | --- | --- | --- | --- | --- | --- | --- |
|  | **OSI+TS (N=434)** | | | | | **USP (N=431)** | | | | |
|  | **Mean** | **SD** | **Min** | **Max** | **% of Use** | **Mean** | **SD** | **Min** | **Max** | **% of Use** |
| **Inpatient service** |  |  |  |  |  |  |  |  |  |  |
| Short stay | 0.01 | 0.10 | 0 | 1 | 0.92% | 0.00 | 0.00 | 0 | 0 | 0.00% |
| Long stay | 0.01 | 0.21 | 0 | 4 | 0.46% | 0.01 | 0.14 | 0 | 3 | 0.23% |
| **Outpatient service** |  |  |  |  |  |  |  |  |  |  |
| Paediatric-general | 0.06 | 0.37 | 0 | 4 | 3.46% | 0.08 | 0.46 | 0 | 5 | 3.94% |
| Paediatric-ophthalmology | 0.06 | 0.38 | 0 | 4 | 3.69% | 0.03 | 0.20 | 0 | 2 | 2.09% |
| Paediatric-audiology | 0.03 | 0.32 | 0 | 6 | 1.38% | 0.01 | 0.12 | 0 | 2 | 0.70% |
| Orthoptics | 0.01 | 0.08 | 0 | 1 | 0.69% | 0.01 | 0.12 | 0 | 2 | 0.70% |
| Paediatric-orthopaedics | 0.02 | 0.19 | 0 | 3 | 1.15% | 0.00 | 0.05 | 0 | 1 | 0.23% |
| Paediatric-dentistry | 0.01 | 0.11 | 0 | 2 | 0.46% | 0.01 | 0.20 | 0 | 4 | 0.46% |
| Paediatric-urology | 0.01 | 0.15 | 0 | 3 | 0.46% | 0.00 | 0.05 | 0 | 1 | 0.23% |
| Paediatric-gastroenterology | 0.00 | 0.00 | 0 | 0 | 0.00% | 0.02 | 0.26 | 0 | 5 | 0.70% |
| Phlebotomy | 0.01 | 0.11 | 0 | 2 | 0.46% | 0.00 | 0.07 | 0 | 1 | 0.46% |
| Other services | 0.07 | 0.45 | 0 | 7 | 4.15% | 0.08 | 0.42 | 0 | 4 | 4.64% |
| **A&E** |  |  |  |  |  |  |  |  |  |  |
| A&E Service | 0.08 | 0.35 | 0 | 4 | 6.68% | 0.09 | 0.33 | 0 | 3 | 7.42% |
| Use of ambulance | 0.01 | 0.20 | 0 | 4 | 0.46% | 0.01 | 0.08 | 0 | 1 | 0.70% |
| **GP & Practice nurse** |  |  |  |  |  |  |  |  |  |  |
| GP visits | 0.50 | 2.29 | 0 | 44 | 24.42% | 0.41 | 0.88 | 0 | 6 | 24.59% |
| Practice nurse visits | 0.06 | 0.29 | 0 | 3 | 5.53% | 0.06 | 0.31 | 0 | 3 | 5.10% |
| **Community service** |  |  |  |  |  |  |  |  |  |  |
| Social worker | 0.14 | 1.52 | 0 | 30 | 3.46% | 0.04 | 0.32 | 0 | 4 | 1.86% |
| Clinical psychologist | 0.00 | 0.05 | 0 | 1 | 0.23% | 0.01 | 0.12 | 0 | 2 | 0.70% |
| CAMHS | 0.04 | 0.42 | 0 | 6 | 1.38% | 0.06 | 0.48 | 0 | 6 | 2.09% |
| Community paediatrician | 0.06 | 0.35 | 0 | 4 | 4.38% | 0.06 | 0.29 | 0 | 3 | 5.34% |
| Community children's nurse or school nurse | 0.05 | 0.26 | 0 | 2 | 3.46% | 0.07 | 0.62 | 0 | 12 | 3.48% |
| Educational psychologist | 0.00 | 0.07 | 0 | 1 | 0.46% | 0.05 | 0.57 | 0 | 10 | 1.16% |
| Special educational needs coordinator | 0.12 | 0.69 | 0 | 10 | 7.14% | 0.13 | 0.65 | 0 | 10 | 7.89% |
| Family liaison officer - school | 0.05 | 0.42 | 0 | 6 | 1.84% | 0.03 | 0.32 | 0 | 6 | 1.39% |
| Teacher | 0.16 | 0.84 | 0 | 10 | 7.14% | 0.24 | 2.07 | 0 | 40 | 7.19% |
| Primary mental health worker | 0.02 | 0.24 | 0 | 4 | 0.46% | 0.00 | 0.05 | 0 | 1 | 0.23% |
| CAMHS nurse | 0.03 | 0.48 | 0 | 10 | 0.46% | 0.00 | 0.00 | 0 | 0 | 0.00% |
| Art therapist | 0.00 | 0.00 | 0 | 0 | 0.00% | 0.01 | 0.19 | 0 | 4 | 0.23% |
| Play therapist | 0.11 | 1.30 | 0 | 24 | 1.38% | 0.08 | 0.80 | 0 | 10 | 1.62% |
| Occupational therapist | 0.01 | 0.14 | 0 | 2 | 1.15% | 0.03 | 0.30 | 0 | 5 | 1.39% |
| Speech and language therapist | 0.28 | 1.68 | 0 | 24 | 6.91% | 0.17 | 1.17 | 0 | 12 | 4.87% |
| Physiotherapist | 0.01 | 0.08 | 0 | 1 | 0.69% | 0.01 | 0.10 | 0 | 1 | 0.93% |
| Family support worker | 0.06 | 0.59 | 0 | 10 | 2.30% | 0.02 | 0.31 | 0 | 6 | 0.93% |
| Dietician | 0.02 | 0.16 | 0 | 2 | 1.84% | 0.02 | 0.14 | 0 | 2 | 1.39% |
| Self-help support group | 0.00 | 0.00 | 0 | 0 | 0.00% | 0.00 | 0.10 | 0 | 2 | 0.23% |
| Complementary therapist | 0.01 | 0.21 | 0 | 4 | 0.46% | 0.00 | 0.00 | 0 | 0 | 0.00% |
| Helpline or advice service | 0.01 | 0.24 | 0 | 5 | 0.23% | 0.00 | 0.00 | 0 | 0 | 0.00% |
| Home start | 0.00 | 0.05 | 0 | 1 | 0.23% | 0.01 | 0.11 | 0 | 2 | 0.46% |
| Family centre | 0.00 | 0.00 | 0 | 0 | 0.00% | 0.00 | 0.07 | 0 | 1 | 0.46% |
| Counsellor | 0.01 | 0.21 | 0 | 4 | 0.46% | 0.00 | 0.00 | 0 | 0 | 0.00% |
| Other service | 0.01 | 0.11 | 0 | 1 | 1.15% | 0.09 | 0.84 | 0 | 12 | 2.09% |
| **School absence due to child anxiety problems** |  |  |  |  |  |  |  |  |  |  |
| School days | 0.35 | 2.43 | 0 | 45 | 10.14% | 0.19 | 0.96 | 0 | 14 | 8.58% |

**Table S5.2: Children’s Service Use at 12 Weeks – Complete Case Analysis**

|  | **Group** | | | | | | | | | |
| --- | --- | --- | --- | --- | --- | --- | --- | --- | --- | --- |
|  | **OSI+TS (N=346)** | | | | | **USP (N=359)** | | | | |
|  | **Mean** | **SD** | **Min** | **Max** | **% of Use** | **Mean** | **SD** | **Min** | **Max** | **% of Use** |
| **Inpatient service** |  |  |  |  |  |  |  |  |  |  |
| Short stay | 0.01 | 0.11 | 0 | 1 | 1.16% | 0.01 | 0.07 | 0 | 1 | 0.56% |
| Long stay | 0.01 | 0.11 | 0 | 2 | 0.29% | 0.00 | 0.00 | 0 | 0 | 0.00% |
| **Outpatient service** |  |  |  |  |  |  |  |  |  |  |
| Paediatric-general | 0.04 | 0.26 | 0 | 3 | 2.89% | 0.03 | 0.21 | 0 | 2 | 1.95% |
| Paediatric-ophthalmology | 0.03 | 0.23 | 0 | 3 | 1.45% | 0.01 | 0.07 | 0 | 1 | 0.56% |
| Paediatric-audiology | 0.01 | 0.13 | 0 | 2 | 0.87% | 0.01 | 0.13 | 0 | 2 | 0.84% |
| Orthoptics | 0.00 | 0.00 | 0 | 0 | 0.00% | 0.00 | 0.05 | 0 | 1 | 0.28% |
| Paediatric-orthopaedics | 0.01 | 0.08 | 0 | 1 | 0.58% | 0.01 | 0.11 | 0 | 2 | 0.28% |
| Paediatric-dentistry | 0.00 | 0.00 | 0 | 0 | 0.00% | 0.02 | 0.20 | 0 | 3 | 0.84% |
| Paediatric-urology | 0.00 | 0.00 | 0 | 0 | 0.00% | 0.00 | 0.05 | 0 | 1 | 0.28% |
| Paediatric-gastroenterology | 0.00 | 0.00 | 0 | 0 | 0.00% | 0.02 | 0.26 | 0 | 4 | 0.84% |
| Phlebotomy | 0.00 | 0.00 | 0 | 0 | 0.00% | 0.00 | 0.05 | 0 | 1 | 0.28% |
| Other services | 0.06 | 0.78 | 0 | 14 | 1.73% | 0.06 | 0.36 | 0 | 4 | 3.62% |
| **A&E** |  |  |  |  |  |  |  |  |  |  |
| A&E Service | 0.08 | 0.38 | 0 | 4 | 6.07% | 0.08 | 0.32 | 0 | 3 | 6.69% |
| Use of ambulance | 0.02 | 0.23 | 0 | 4 | 0.87% | 0.00 | 0.00 | 0 | 0 | 0.00% |
| **GP & Practice nurse** |  |  |  |  |  |  |  |  |  |  |
| GP visits | 0.29 | 0.84 | 0 | 8 | 17.92% | 0.30 | 0.77 | 0 | 6 | 19.78% |
| Practice nurse visits | 0.08 | 0.37 | 0 | 4 | 5.20% | 0.04 | 0.33 | 0 | 4 | 2.23% |
| **Community service** |  |  |  |  |  |  |  |  |  |  |
| Social worker | 0.05 | 0.38 | 0 | 6 | 2.89% | 0.02 | 0.23 | 0 | 4 | 1.11% |
| Clinical psychologist | 0.03 | 0.34 | 0 | 6 | 1.16% | 0.00 | 0.00 | 0 | 0 | 0.00% |
| CAMHS | 0.03 | 0.23 | 0 | 3 | 2.31% | 0.09 | 0.84 | 0 | 13 | 2.51% |
| Community paediatrician | 0.07 | 0.68 | 0 | 12 | 2.60% | 0.04 | 0.22 | 0 | 2 | 3.34% |
| Community children's nurse or school nurse | 0.03 | 0.18 | 0 | 1 | 3.18% | 0.06 | 0.38 | 0 | 5 | 3.90% |
| Educational psychologist | 0.01 | 0.14 | 0 | 2 | 1.16% | 0.02 | 0.16 | 0 | 2 | 1.67% |
| Special educational needs coordinator | 0.11 | 0.41 | 0 | 3 | 7.80% | 0.15 | 0.73 | 0 | 10 | 8.08% |
| Family liaison officer - school | 0.01 | 0.11 | 0 | 2 | 0.29% | 0.11 | 0.84 | 0 | 12 | 2.79% |
| Teacher | 0.12 | 0.60 | 0 | 6 | 6.07% | 0.09 | 0.35 | 0 | 3 | 6.69% |
| Primary mental health worker | 0.00 | 0.00 | 0 | 0 | 0.00% | 0.01 | 0.26 | 0 | 5 | 0.28% |
| CAMHS nurse | 0.00 | 0.00 | 0 | 0 | 0.00% | 0.01 | 0.22 | 0 | 4 | 0.56% |
| Family therapist | 0.01 | 0.16 | 0 | 3 | 0.29% | 0.00 | 0.05 | 0 | 1 | 0.28% |
| Play therapist | 0.09 | 0.77 | 0 | 8 | 2.02% | 0.12 | 0.93 | 0 | 12 | 3.06% |
| Occupational therapist | 0.03 | 0.35 | 0 | 6 | 1.16% | 0.03 | 0.43 | 0 | 8 | 1.39% |
| Speech and language therapist | 0.11 | 0.70 | 0 | 8 | 4.34% | 0.14 | 0.99 | 0 | 14 | 5.29% |
| Physiotherapist | 0.01 | 0.12 | 0 | 1 | 1.45% | 0.01 | 0.07 | 0 | 1 | 0.56% |
| Family support worker | 0.09 | 0.94 | 0 | 15 | 2.02% | 0.02 | 0.18 | 0 | 2 | 1.67% |
| Dietician | 0.01 | 0.22 | 0 | 4 | 0.29% | 0.01 | 0.09 | 0 | 1 | 0.84% |
| Complementary therapist | 0.02 | 0.25 | 0 | 3 | 0.87% | 0.01 | 0.26 | 0 | 5 | 0.28% |
| Home start | 0.00 | 0.05 | 0 | 1 | 0.29% | 0.00 | 0.05 | 0 | 1 | 0.28% |
| Family centre | 0.00 | 0.00 | 0 | 0 | 0.00% | 0.00 | 0.05 | 0 | 1 | 0.28% |
| Counsellor | 0.01 | 0.22 | 0 | 4 | 0.29% | 0.03 | 0.38 | 0 | 7 | 0.56% |
| Other service | 0.05 | 0.54 | 0 | 8 | 1.16% | 0.03 | 0.27 | 0 | 3 | 1.67% |
| **School absence due to child anxiety problems** |  |  |  |  |  |  |  |  |  |  |
| School days | 0.16 | 0.80 | 0 | 8 | 7.80% | 0.30 | 1.45 | 0 | 20 | 11.42% |

**Table S5.3: Children’s Service Use at 12 Months – Complete Case Analysis**

|  | **Group** | | | | | | | | | |
| --- | --- | --- | --- | --- | --- | --- | --- | --- | --- | --- |
|  | **OSI+TS (N=307)** | | | | | **USP (N=313)** | | | | |
|  | **Mean** | **SD** | **Min** | **Max** | **% of Use** | **Mean** | **SD** | **Min** | **Max** | **% of Use** |
| **Inpatient service** |  |  |  |  |  |  |  |  |  |  |
| Short stay | 0.01 | 0.08 | 0 | 1 | 0.65% | 0.00 | 0.06 | 0 | 1 | 0.32% |
| Long stay | 0.06 | 0.67 | 0 | 11 | 1.63% | 0.00 | 0.00 | 0 | 0 | 0.00% |
| **Outpatient service** |  |  |  |  |  |  |  |  |  |  |
| Paediatric-general | 0.12 | 0.68 | 0 | 6 | 4.56% | 0.15 | 0.60 | 0 | 4 | 7.35% |
| Paediatric-ophthalmology | 0.07 | 0.68 | 0 | 11 | 1.63% | 0.03 | 0.29 | 0 | 4 | 0.96% |
| Paediatric-audiology | 0.03 | 0.25 | 0 | 3 | 1.63% | 0.02 | 0.18 | 0 | 2 | 1.28% |
| Orthoptics | 0.01 | 0.17 | 0 | 3 | 0.33% | 0.01 | 0.10 | 0 | 1 | 0.96% |
| Paediatric-orthopaedics | 0.01 | 0.13 | 0 | 2 | 0.65% | 0.07 | 0.59 | 0 | 8 | 2.24% |
| Paediatric-dentistry | 0.02 | 0.29 | 0 | 5 | 0.33% | 0.01 | 0.11 | 0 | 2 | 0.32% |
| Paediatric-urology | 0.01 | 0.08 | 0 | 1 | 0.65% | 0.03 | 0.27 | 0 | 3 | 1.28% |
| Paediatric-gastroenterology | 0.02 | 0.24 | 0 | 4 | 0.65% | 0.02 | 0.30 | 0 | 5 | 0.64% |
| Phlebotomy | 0.01 | 0.13 | 0 | 2 | 0.65% | 0.01 | 0.08 | 0 | 1 | 0.64% |
| Other services | 0.23 | 1.29 | 0 | 17 | 7.82% | 0.14 | 0.63 | 0 | 6 | 7.03% |
| **A&E** |  |  |  |  |  |  |  |  |  |  |
| A&E Service | 0.13 | 0.45 | 0 | 4 | 10.10% | 0.14 | 0.38 | 0 | 2 | 12.46% |
| Use of ambulance | 0.01 | 0.14 | 0 | 2 | 0.98% | 0.00 | 0.00 | 0 | 0 | 0.00% |
| **GP & Practice nurse** |  |  |  |  |  |  |  |  |  |  |
| GP visits | 0.70 | 1.34 | 0 | 11 | 34.53% | 0.66 | 1.29 | 0 | 10 | 34.19% |
| Practice nurse visits | 0.10 | 0.42 | 0 | 3 | 6.19% | 0.20 | 1.28 | 0 | 20 | 8.63% |
| **Community service** |  |  |  |  |  |  |  |  |  |  |
| Social worker | 0.07 | 0.48 | 0 | 5 | 3.26% | 0.04 | 0.32 | 0 | 3 | 1.60% |
| Clinical psychologist | 0.03 | 0.31 | 0 | 4 | 0.98% | 0.03 | 0.30 | 0 | 5 | 1.28% |
| CAMHS | 0.08 | 0.62 | 0 | 7 | 3.26% | 0.21 | 1.59 | 0 | 20 | 2.56% |
| Community paediatrician | 0.06 | 0.59 | 0 | 10 | 2.61% | 0.07 | 0.34 | 0 | 3 | 5.11% |
| Community children's nurse or school nurse | 0.05 | 0.35 | 0 | 4 | 2.61% | 0.11 | 1.43 | 0 | 25 | 2.88% |
| Educational psychologist | 0.04 | 0.25 | 0 | 3 | 2.61% | 0.11 | 0.75 | 0 | 10 | 3.83% |
| Special educational needs coordinator | 0.24 | 0.99 | 0 | 10 | 9.77% | 0.35 | 1.45 | 0 | 15 | 12.14% |
| Family liaison officer - school | 0.01 | 0.18 | 0 | 3 | 0.65% | 0.09 | 0.72 | 0 | 10 | 2.88% |
| Teacher | 0.40 | 1.99 | 0 | 20 | 11.07% | 0.25 | 1.04 | 0 | 10 | 10.86% |
| Primary mental health worker | 0.00 | 0.06 | 0 | 1 | 0.33% | 0.01 | 0.10 | 0 | 1 | 0.96% |
| CAMHS nurse | 0.00 | 0.06 | 0 | 1 | 0.33% | 0.00 | 0.00 | 0 | 0 | 0.00% |
| Family therapist | 0.02 | 0.34 | 0 | 6 | 0.33% | 0.02 | 0.28 | 0 | 5 | 0.32% |
| Art therapist | 0.00 | 0.06 | 0 | 1 | 0.33% | 0.03 | 0.57 | 0 | 10 | 0.32% |
| Play therapist | 0.12 | 1.07 | 0 | 12 | 1.63% | 0.29 | 2.25 | 0 | 30 | 2.56% |
| Occupational therapist | 0.03 | 0.28 | 0 | 3 | 1.63% | 0.15 | 1.41 | 0 | 20 | 2.56% |
| Speech and language therapist | 0.20 | 1.26 | 0 | 15 | 4.89% | 0.05 | 0.37 | 0 | 5 | 2.56% |
| Physiotherapist | 0.05 | 0.39 | 0 | 5 | 1.95% | 0.06 | 0.62 | 0 | 10 | 1.28% |
| Family support worker | 0.16 | 1.41 | 0 | 22 | 3.58% | 0.09 | 0.67 | 0 | 7 | 2.56% |
| Dietician | 0.02 | 0.19 | 0 | 3 | 0.98% | 0.02 | 0.15 | 0 | 2 | 1.28% |
| Complementary therapist | 0.02 | 0.34 | 0 | 6 | 0.33% | 0.05 | 0.70 | 0 | 12 | 0.96% |
| Home start | 0.02 | 0.29 | 0 | 5 | 0.33% | 0.00 | 0.00 | 0 | 0 | 0.00% |
| Counsellor | 0.06 | 0.57 | 0 | 6 | 1.30% | 0.32 | 1.77 | 0 | 20 | 5.11% |
| IAPT | 0.00 | 0.06 | 0 | 1 | 0.33% | 0.00 | 0.00 | 0 | 0 | 0.00% |
| Other service | 0.06 | 0.54 | 0 | 7 | 1.95% | 0.00 | 0.00 | 0 | 0 | 0.00% |
| **School absence due to child anxiety problems** |  |  |  |  |  |  |  |  |  |  |
| School days | 0.60 | 3.57 | 0 | 41 | 11.73% | 0.52 | 2.02 | 0 | 20 | 14.38% |

**Table S5.4: Parents’ Service Use at Baseline – Complete Case Analysis**

|  | **Group** | | | | | | | | | |
| --- | --- | --- | --- | --- | --- | --- | --- | --- | --- | --- |
|  | **OSI+TS (N=434)** | | | | | **USP (N=431)** | | | | |
|  | **Mean** | **SD** | **Min** | **Max** | **% of Use** | **Mean** | **SD** | **Min** | **Max** | **% of Use** |
| **Inpatient service** |  |  |  |  |  |  |  |  |  |  |
| Short stay | 0.01 | 0.11 | 0 | 1 | 1.15% | 0.01 | 0.12 | 0 | 2 | 0.70% |
| Long stay | 0.02 | 0.40 | 0 | 8 | 0.46% | 0.17 | 2.05 | 0 | 40 | 2.09% |
| **Outpatient service** |  |  |  |  |  |  |  |  |  |  |
| General | 0.03 | 0.27 | 0 | 3 | 1.61% | 0.10 | 0.90 | 0 | 16 | 2.32% |
| Imaging | 0.02 | 0.17 | 0 | 2 | 1.38% | 0.03 | 0.26 | 0 | 3 | 2.32% |
| Gastroenterology | 0.03 | 0.25 | 0 | 3 | 2.30% | 0.01 | 0.13 | 0 | 2 | 0.93% |
| Gynaecology | 0.05 | 0.39 | 0 | 4 | 1.61% | 0.04 | 0.35 | 0 | 4 | 1.62% |
| Orthopaedics | 0.06 | 0.66 | 0 | 12 | 1.38% | 0.02 | 0.22 | 0 | 4 | 0.70% |
| Dermatology | 0.03 | 0.25 | 0 | 4 | 1.38% | 0.02 | 0.18 | 0 | 3 | 1.39% |
| Physiotherapy | 0.04 | 0.49 | 0 | 8 | 1.15% | 0.01 | 0.20 | 0 | 4 | 0.70% |
| Neurology | 0.03 | 0.25 | 0 | 4 | 1.38% | 0.01 | 0.11 | 0 | 2 | 0.46% |
| Obstetrics | 0.04 | 0.60 | 0 | 12 | 0.92% | 0.07 | 1.09 | 0 | 22 | 0.93% |
| Other services | 0.13 | 0.68 | 0 | 8 | 5.76% | 0.19 | 0.87 | 0 | 7 | 7.66% |
| **A&E** |  |  |  |  |  |  |  |  |  |  |
| A&E Service | 0.07 | 0.31 | 0 | 3 | 5.53% | 0.06 | 0.49 | 0 | 9 | 3.94% |
| Use of ambulance | 0.01 | 0.08 | 0 | 1 | 0.69% | 0.00 | 0.00 | 0 | 0 | 0.00% |
| **GP & Practice nurse** |  |  |  |  |  |  |  |  |  |  |
| GP | 0.81 | 1.43 | 0 | 10 | 39.17% | 0.85 | 1.41 | 0 | 8 | 39.21% |
| Practice nurse | 0.39 | 3.60 | 0 | 74 | 15.21% | 0.19 | 0.59 | 0 | 6 | 13.69% |
| **Community service** |  |  |  |  |  |  |  |  |  |  |
| Social worker | 0.09 | 0.65 | 0 | 10 | 3.23% | 0.04 | 0.35 | 0 | 6 | 2.55% |
| Clinical psychologist | 0.07 | 0.60 | 0 | 10 | 2.07% | 0.05 | 0.43 | 0 | 6 | 2.09% |
| CAMHS | 0.05 | 0.26 | 0 | 3 | 3.69% | 0.03 | 0.42 | 0 | 8 | 1.16% |
| Community psychiatric nurse | 0.01 | 0.16 | 0 | 3 | 0.69% | 0.05 | 0.46 | 0 | 6 | 1.62% |
| Community paediatrician | 0.01 | 0.10 | 0 | 1 | 0.92% | 0.00 | 0.07 | 0 | 1 | 0.46% |
| Community children's nurse or school nurse | 0.02 | 0.17 | 0 | 3 | 1.15% | 0.00 | 0.07 | 0 | 1 | 0.46% |
| Educational psychologist | 0.01 | 0.13 | 0 | 2 | 0.92% | 0.01 | 0.14 | 0 | 2 | 0.70% |
| Special educational needs coordinator | 0.11 | 1.24 | 0 | 25 | 3.92% | 0.09 | 0.41 | 0 | 5 | 6.50% |
| Family liaison officer - school | 0.03 | 0.29 | 0 | 5 | 1.84% | 0.03 | 0.27 | 0 | 4 | 2.09% |
| Teacher | 0.18 | 1.08 | 0 | 20 | 9.45% | 0.13 | 0.51 | 0 | 6 | 8.35% |
| Primary mental health worker | 0.04 | 0.41 | 0 | 6 | 1.15% | 0.08 | 0.82 | 0 | 12 | 1.62% |
| CAMHS nurse | 0.00 | 0.07 | 0 | 1 | 0.46% | 0.00 | 0.00 | 0 | 0 | 0.00% |
| Family therapist | 0.03 | 0.37 | 0 | 6 | 0.69% | 0.12 | 1.05 | 0 | 12 | 1.39% |
| Art therapist | 0.00 | 0.00 | 0 | 0 | 0.00% | 0.03 | 0.58 | 0 | 12 | 0.23% |
| Play therapist | 0.01 | 0.29 | 0 | 6 | 0.23% | 0.00 | 0.05 | 0 | 1 | 0.23% |
| Occupational therapist | 0.02 | 0.17 | 0 | 2 | 1.61% | 0.01 | 0.11 | 0 | 1 | 1.16% |
| Speech and language therapist | 0.01 | 0.14 | 0 | 2 | 1.15% | 0.01 | 0.11 | 0 | 1 | 1.16% |
| Physiotherapist | 0.06 | 0.37 | 0 | 5 | 3.69% | 0.05 | 0.32 | 0 | 3 | 3.02% |
| Family support worker | 0.07 | 0.46 | 0 | 6 | 3.46% | 0.07 | 0.56 | 0 | 8 | 2.55% |
| Family planning | 0.01 | 0.18 | 0 | 3 | 0.69% | 0.01 | 0.11 | 0 | 1 | 1.16% |
| Dietician | 0.00 | 0.00 | 0 | 0 | 0.00% | 0.01 | 0.15 | 0 | 2 | 0.93% |
| Alcohol and drug support worker | 0.00 | 0.00 | 0 | 0 | 0.00% | 0.03 | 0.58 | 0 | 12 | 0.23% |
| Relationship/marriage guidance | 0.03 | 0.42 | 0 | 8 | 1.38% | 0.03 | 0.48 | 0 | 10 | 0.46% |
| Self-help support group | 0.07 | 0.62 | 0 | 9 | 1.84% | 0.09 | 0.89 | 0 | 12 | 1.62% |
| Complementary therapist | 0.06 | 0.45 | 0 | 6 | 2.53% | 0.07 | 0.61 | 0 | 10 | 2.09% |
| Helpline or advice service | 0.03 | 0.27 | 0 | 4 | 1.61% | 0.06 | 0.78 | 0 | 12 | 1.16% |
| Home start | 0.02 | 0.39 | 0 | 8 | 0.46% | 0.02 | 0.39 | 0 | 8 | 0.46% |
| Family centre | 0.00 | 0.07 | 0 | 1 | 0.46% | 0.09 | 0.95 | 0 | 12 | 1.39% |
| Housing department | 0.00 | 0.07 | 0 | 1 | 0.46% | 0.04 | 0.55 | 0 | 8 | 1.16% |
| Counsellor | 0.10 | 1.09 | 0 | 20 | 1.61% | 0.10 | 0.83 | 0 | 12 | 2.09% |
| IAPT | 0.01 | 0.14 | 0 | 3 | 0.23% | 0.01 | 0.11 | 0 | 2 | 0.46% |
| Other services | 0.15 | 1.07 | 0 | 12 | 2.76% | 0.17 | 1.15 | 0 | 12 | 3.02% |
| **Absenteeism due to child anxiety problems** |  |  |  |  |  |  |  |  |  |  |
| Days off work | 0.32 | 3.20 | 0 | 64 | 6.91% | 0.12 | 0.75 | 0 | 10 | 4.41% |

**Table S5.5: Parents’ Service Use at 12 Months – Complete Case Analysis**

|  | **Group** | | | | | | | | | |
| --- | --- | --- | --- | --- | --- | --- | --- | --- | --- | --- |
|  | **OSI+TS (N=346)** | | | | | **USP (N=359)** | | | | |
|  | **Mean** | **SD** | **Min** | **Max** | **% of Use** | **Mean** | **SD** | **Min** | **Max** | **% of Use** |
| **Inpatient service** |  |  |  |  |  |  |  |  |  |  |
| Short stay | 0.01 | 0.11 | 0 | 2 | 0.29% | 0.01 | 0.07 | 0 | 1 | 0.56% |
| Long stay | 0.02 | 0.22 | 0 | 3 | 0.87% | 0.04 | 0.56 | 0 | 10 | 0.84% |
| **Outpatient service** |  |  |  |  |  |  |  |  |  |  |
| General | 0.03 | 0.36 | 0 | 5 | 1.16% | 0.03 | 0.31 | 0 | 5 | 1.11% |
| Imaging | 0.01 | 0.08 | 0 | 1 | 0.58% | 0.03 | 0.32 | 0 | 5 | 1.39% |
| Gastroenterology | 0.00 | 0.05 | 0 | 1 | 0.29% | 0.01 | 0.17 | 0 | 3 | 0.56% |
| Gynaecology | 0.04 | 0.44 | 0 | 7 | 1.45% | 0.01 | 0.13 | 0 | 2 | 0.84% |
| Orthopaedics | 0.04 | 0.44 | 0 | 7 | 1.45% | 0.01 | 0.19 | 0 | 3 | 0.56% |
| Dermatology | 0.00 | 0.05 | 0 | 1 | 0.29% | 0.01 | 0.15 | 0 | 2 | 0.56% |
| Physiotherapy | 0.03 | 0.31 | 0 | 5 | 1.16% | 0.01 | 0.12 | 0 | 2 | 0.56% |
| Neurology | 0.01 | 0.12 | 0 | 2 | 0.58% | 0.01 | 0.13 | 0 | 2 | 0.84% |
| Obstetrics | 0.05 | 0.62 | 0 | 10 | 0.87% | 0.02 | 0.24 | 0 | 4 | 0.56% |
| Other services | 0.13 | 0.79 | 0 | 12 | 5.78% | 0.14 | 1.22 | 0 | 20 | 3.90% |
| **A&E** |  |  |  |  |  |  |  |  |  |  |
| A&E Service | 0.05 | 0.27 | 0 | 2 | 4.05% | 0.06 | 0.38 | 0 | 4 | 3.62% |
| Use of ambulance | 0.00 | 0.05 | 0 | 1 | 0.29% | 0.00 | 0.05 | 0 | 1 | 0.28% |
| **GP & Practice nurse** |  |  |  |  |  |  |  |  |  |  |
| GP | 0.61 | 1.13 | 0 | 8 | 32.95% | 0.70 | 1.38 | 0 | 11 | 33.15% |
| Practice nurse | 0.19 | 0.75 | 0 | 10 | 12.43% | 0.22 | 0.86 | 0 | 12 | 13.65% |
| **Community service** |  |  |  |  |  |  |  |  |  |  |
| Social worker | 0.06 | 0.59 | 0 | 10 | 2.31% | 0.04 | 0.28 | 0 | 4 | 2.79% |
| Clinical psychologist | 0.02 | 0.21 | 0 | 3 | 1.16% | 0.06 | 0.58 | 0 | 10 | 2.51% |
| CAMHS | 0.03 | 0.33 | 0 | 5 | 1.16% | 0.05 | 0.37 | 0 | 5 | 2.79% |
| Community psychiatric nurse | 0.00 | 0.00 | 0 | 0 | 0.00% | 0.05 | 0.40 | 0 | 6 | 2.23% |
| Community paediatrician | 0.00 | 0.00 | 0 | 0 | 0.00% | 0.01 | 0.12 | 0 | 1 | 1.39% |
| Community children's nurse or school nurse | 0.00 | 0.05 | 0 | 1 | 0.29% | 0.02 | 0.14 | 0 | 1 | 1.95% |
| Educational psychologist | 0.00 | 0.05 | 0 | 1 | 0.29% | 0.01 | 0.12 | 0 | 1 | 1.39% |
| Special educational needs coordinator | 0.04 | 0.22 | 0 | 2 | 3.18% | 0.09 | 0.37 | 0 | 3 | 7.24% |
| Family liaison officer - school | 0.01 | 0.08 | 0 | 1 | 0.58% | 0.05 | 0.35 | 0 | 4 | 2.79% |
| Teacher | 0.08 | 0.38 | 0 | 5 | 5.49% | 0.09 | 0.38 | 0 | 3 | 6.13% |
| Primary mental health worker | 0.04 | 0.36 | 0 | 5 | 1.73% | 0.04 | 0.33 | 0 | 4 | 2.23% |
| CAMHS nurse | 0.00 | 0.00 | 0 | 0 | 0.00% | 0.02 | 0.18 | 0 | 3 | 1.11% |
| Family therapist | 0.02 | 0.38 | 0 | 7 | 0.29% | 0.10 | 0.87 | 0 | 12 | 2.51% |
| Art therapist | 0.00 | 0.00 | 0 | 0 | 0.00% | 0.02 | 0.23 | 0 | 4 | 1.11% |
| Play therapist | 0.02 | 0.38 | 0 | 7 | 0.29% | 0.02 | 0.15 | 0 | 2 | 1.39% |
| Music therapist | 0.00 | 0.05 | 0 | 1 | 0.29% | 0.01 | 0.07 | 0 | 1 | 0.56% |
| Occupational therapist | 0.01 | 0.18 | 0 | 3 | 0.87% | 0.02 | 0.13 | 0 | 1 | 1.67% |
| Speech and language therapist | 0.03 | 0.34 | 0 | 5 | 1.45% | 0.02 | 0.17 | 0 | 2 | 1.95% |
| Physiotherapist | 0.09 | 0.55 | 0 | 5 | 3.76% | 0.09 | 0.64 | 0 | 10 | 4.18% |
| Family support worker | 0.05 | 0.40 | 0 | 4 | 2.02% | 0.03 | 0.22 | 0 | 3 | 1.95% |
| Family planning | 0.01 | 0.09 | 0 | 1 | 0.87% | 0.03 | 0.17 | 0 | 2 | 2.23% |
| Dietician | 0.01 | 0.12 | 0 | 2 | 0.58% | 0.02 | 0.13 | 0 | 1 | 1.67% |
| Alcohol and drug support worker | 0.00 | 0.00 | 0 | 0 | 0.00% | 0.01 | 0.09 | 0 | 1 | 0.84% |
| Relationship/marriage guidance | 0.03 | 0.45 | 0 | 8 | 0.87% | 0.02 | 0.19 | 0 | 3 | 1.39% |
| Self-help support group | 0.08 | 0.94 | 0 | 14 | 1.16% | 0.06 | 0.45 | 0 | 6 | 2.51% |
| Complementary therapist | 0.06 | 0.41 | 0 | 4 | 2.60% | 0.06 | 0.37 | 0 | 4 | 2.79% |
| Helpline or advice service | 0.04 | 0.47 | 0 | 7 | 1.16% | 0.02 | 0.13 | 0 | 1 | 1.67% |
| Home start | 0.01 | 0.08 | 0 | 1 | 0.58% | 0.01 | 0.11 | 0 | 1 | 1.11% |
| Family centre | 0.00 | 0.05 | 0 | 1 | 0.29% | 0.01 | 0.09 | 0 | 1 | 0.84% |
| Housing department | 0.01 | 0.09 | 0 | 1 | 0.87% | 0.01 | 0.09 | 0 | 1 | 0.84% |
| Counsellor | 0.05 | 0.54 | 0 | 9 | 1.45% | 0.09 | 0.90 | 0 | 12 | 1.39% |
| IAPT | 0.00 | 0.05 | 0 | 1 | 0.29% | 0.03 | 0.53 | 0 | 10 | 0.28% |
| Other services | 0.05 | 0.45 | 0 | 7 | 1.73% | 0.02 | 0.20 | 0 | 3 | 1.11% |
| **Absenteeism due to child anxiety problems** |  |  |  |  |  |  |  |  |  |  |
| Days off work | 0.23 | 1.47 | 0 | 16 | 5.49% | 0.11 | 0.51 | 0 | 5 | 6.96% |

**Table S5.6: Parents’ Service Use at 12 Months – Complete Case Analysis**

|  | **Group** | | | | | | | | | |
| --- | --- | --- | --- | --- | --- | --- | --- | --- | --- | --- |
|  | **OSI+TS (N=307)** | | | | | **USP (N=313)** | | | | |
|  | **Mean** | **SD** | **Min** | **Max** | **% of Use** | **Mean** | **SD** | **Min** | **Max** | **% of Use** |
| **Inpatient service** |  |  |  |  |  |  |  |  |  |  |
| Short stay | 0.02 | 0.17 | 0 | 2 | 1.95% | 0.02 | 0.16 | 0 | 2 | 1.60% |
| Long stay | 0.26 | 1.89 | 0 | 29 | 4.23% | 0.14 | 0.91 | 0 | 11 | 2.88% |
| **Outpatient service** |  |  |  |  |  |  |  |  |  |  |
| General | 0.04 | 0.33 | 0 | 4 | 1.63% | 0.03 | 0.33 | 0 | 5 | 0.64% |
| Imaging | 0.01 | 0.14 | 0 | 2 | 0.98% | 0.01 | 0.11 | 0 | 1 | 1.28% |
| Gastroenterology | 0.04 | 0.34 | 0 | 5 | 1.63% | 0.03 | 0.24 | 0 | 3 | 1.28% |
| Gynaecology | 0.03 | 0.22 | 0 | 2 | 1.95% | 0.09 | 0.74 | 0 | 9 | 1.60% |
| Orthopaedics | 0.09 | 0.85 | 0 | 13 | 2.28% | 0.06 | 0.82 | 0 | 14 | 0.64% |
| Dermatology | 0.06 | 0.46 | 0 | 5 | 1.95% | 0.02 | 0.17 | 0 | 2 | 0.96% |
| Physiotherapy | 0.06 | 0.54 | 0 | 6 | 1.63% | 0.02 | 0.34 | 0 | 6 | 0.32% |
| Neurology | 0.04 | 0.39 | 0 | 5 | 1.30% | 0.01 | 0.16 | 0 | 2 | 0.64% |
| Obstetrics | 0.01 | 0.23 | 0 | 4 | 0.33% | 0.05 | 0.71 | 0 | 11 | 0.64% |
| Other services | 0.39 | 1.77 | 0 | 16 | 9.12% | 0.34 | 1.50 | 0 | 15 | 8.63% |
| **A&E** |  |  |  |  |  |  |  |  |  |  |
| A&E Service | 0.11 | 0.39 | 0 | 3 | 8.47% | 0.11 | 0.51 | 0 | 5 | 5.75% |
| Use of ambulance | 0.00 | 0.00 | 0 | 0 | 0.00% | 0.01 | 0.14 | 0 | 2 | 0.96% |
| **GP & Practice nurse** |  |  |  |  |  |  |  |  |  |  |
| GP | 1.27 | 2.27 | 0 | 18 | 44.95% | 1.18 | 2.26 | 0 | 21 | 40.26% |
| practice nurse | 0.31 | 0.84 | 0 | 7 | 18.24% | 0.30 | 1.19 | 0 | 13 | 13.74% |
| **Community service** |  |  |  |  |  |  |  |  |  |  |
| Social worker | 0.11 | 0.82 | 0 | 10 | 2.93% | 0.06 | 0.46 | 0 | 6 | 2.88% |
| Clinical psychologist | 0.15 | 1.45 | 0 | 20 | 2.28% | 0.24 | 2.41 | 0 | 40 | 3.83% |
| CAMHS | 0.04 | 0.32 | 0 | 4 | 2.61% | 0.06 | 0.36 | 0 | 4 | 3.19% |
| Community psychiatric nurse | 0.00 | 0.00 | 0 | 0 | 0.00% | 0.07 | 0.66 | 0 | 10 | 2.24% |
| Community paediatrician | 0.01 | 0.08 | 0 | 1 | 0.65% | 0.04 | 0.25 | 0 | 3 | 2.88% |
| Community children's nurse or school nurse | 0.01 | 0.18 | 0 | 3 | 0.65% | 0.21 | 3.39 | 0 | 60 | 2.24% |
| Educational psychologist | 0.02 | 0.17 | 0 | 2 | 0.98% | 0.04 | 0.23 | 0 | 2 | 3.51% |
| Special educational needs coordinator | 0.40 | 2.14 | 0 | 30 | 12.38% | 0.33 | 1.46 | 0 | 18 | 15.34% |
| Family liaison officer - school | 0.04 | 0.52 | 0 | 9 | 1.30% | 0.06 | 0.36 | 0 | 3 | 3.83% |
| Teacher | 0.52 | 1.50 | 0 | 10 | 20.85% | 0.44 | 1.30 | 0 | 10 | 17.89% |
| Primary mental health worker | 0.04 | 0.57 | 0 | 10 | 0.65% | 0.06 | 0.64 | 0 | 10 | 1.92% |
| CAMHS nurse | 0.01 | 0.13 | 0 | 2 | 0.65% | 0.02 | 0.15 | 0 | 2 | 1.28% |
| Family therapist | 0.03 | 0.36 | 0 | 6 | 0.65% | 0.06 | 0.70 | 0 | 10 | 1.60% |
| Art therapist | 0.00 | 0.00 | 0 | 0 | 0.00% | 0.01 | 0.10 | 0 | 1 | 0.96% |
| Play therapist | 0.00 | 0.00 | 0 | 0 | 0.00% | 0.04 | 0.58 | 0 | 10 | 1.60% |
| Music therapist | 0.00 | 0.00 | 0 | 0 | 0.00% | 0.01 | 0.10 | 0 | 1 | 0.96% |
| Occupational therapist | 0.03 | 0.25 | 0 | 3 | 1.30% | 0.12 | 1.32 | 0 | 23 | 3.51% |
| Speech and language therapist | 0.02 | 0.24 | 0 | 4 | 0.98% | 0.01 | 0.10 | 0 | 1 | 0.96% |
| Physiotherapist | 0.22 | 2.36 | 0 | 40 | 3.26% | 0.12 | 0.94 | 0 | 12 | 4.15% |
| Family support worker | 0.06 | 0.40 | 0 | 5 | 3.26% | 0.10 | 0.46 | 0 | 4 | 5.43% |
| Family planning | 0.00 | 0.06 | 0 | 1 | 0.33% | 0.03 | 0.22 | 0 | 3 | 2.24% |
| Dietician | 0.01 | 0.13 | 0 | 2 | 0.65% | 0.01 | 0.11 | 0 | 1 | 1.28% |
| Alcohol and drug support worker | 0.00 | 0.00 | 0 | 0 | 0.00% | 0.01 | 0.10 | 0 | 1 | 0.96% |
| Relationship/marriage guidance | 0.06 | 0.72 | 0 | 12 | 1.30% | 0.03 | 0.41 | 0 | 7 | 1.28% |
| Self-help support group | 0.18 | 1.96 | 0 | 30 | 1.63% | 0.07 | 0.68 | 0 | 11 | 2.56% |
| Complementary therapist | 0.15 | 1.10 | 0 | 10 | 2.28% | 0.20 | 1.49 | 0 | 20 | 4.15% |
| Helpline or advice service | 0.03 | 0.36 | 0 | 6 | 0.65% | 0.03 | 0.20 | 0 | 2 | 2.24% |
| Home start | 0.01 | 0.08 | 0 | 1 | 0.65% | 0.01 | 0.10 | 0 | 1 | 0.96% |
| Family centre | 0.00 | 0.00 | 0 | 0 | 0.00% | 0.02 | 0.20 | 0 | 3 | 1.60% |
| Housing department | 0.01 | 0.10 | 0 | 1 | 0.98% | 0.05 | 0.40 | 0 | 5 | 2.24% |
| Counsellor | 0.60 | 3.56 | 0 | 42 | 6.51% | 0.60 | 3.51 | 0 | 41 | 7.35% |
| IAPT | 0.16 | 1.11 | 0 | 12 | 2.93% | 0.01 | 0.10 | 0 | 1 | 0.96% |
| Other services | 0.06 | 0.64 | 0 | 10 | 1.30% | 0.13 | 1.89 | 0 | 33 | 1.28% |
| **Absenteeism due to child anxiety problems** |  |  |  |  |  |  |  |  |  |  |
| Days off work | 0.58 | 4.34 | 0 | 60 | 7.17% | 0.70 | 5.58 | 0 | 75 | 10.22% |

**Table S6.1: Unit Costs for Outpatient Services (2021/2022 P**rices)

| **Items** | **Unit Cost (Face to Face attendance)** | **Unit Cost (Non-Face to Face Attendance)** | **Source** | **Notes** |
| --- | --- | --- | --- | --- |
| ***Child services*** | | | | |
| Paediatric-General | £301.31 | £261.85 | 2021/22 National Cost Collection Data.  https://www.england.nhs.uk/publication/2021-22-national-cost-collection-data-publication/ (Accessed 9 Jan 2024). | Weighted mean of Consultant Led Non-Admitted Face-to-Face Attendance (for the face-to-face price) or Non-Face-to-Face (for the non-face-to-face price) paediatric services, First |
| Paediatric - Ophthalmology | £161.06 | £135.78 | 2021/22 National Cost Collection Data.  https://www.england.nhs.uk/publication/2021-22-national-cost-collection-data-publication/ (Accessed 9 Jan 2024). | Paediatric Ophthalmology service, Consultant Led, Non-Admitted, First |
| Paediatric-ENT | £175.21 | £166.85 | 2021/22 National Cost Collection Data.  https://www.england.nhs.uk/publication/2021-22-national-cost-collection-data-publication/ (Accessed 9 Jan 2024). | Paediatric Ear Nose and Throat service, Consultant Led, Non-Admitted, First |
| Paediatric-audiology | £142.66 | £327.37 | 2021/22 National Cost Collection Data.  https://www.england.nhs.uk/publication/2021-22-national-cost-collection-data-publication/ (Accessed 9 Jan 2024). | Paediatric Audio Vestibular Medicine service, Consultant Led, Non-Admitted, First |
| Orthoptics | £145.15 | £54.15 | 2021/22 National Cost Collection Data.  https://www.england.nhs.uk/publication/2021-22-national-cost-collection-data-publication/ (Accessed 9 Jan 2024). | Orthoptic service, Consultant Led, Non-Admitted, First |
| Paediatric- Orthopaedics | £190.10 | £84.95 | 2021/22 National Cost Collection Data.  https://www.england.nhs.uk/publication/2021-22-national-cost-collection-data-publication/ (Accessed 9 Jan 2024). | Paediatric Trauma and Orthopaedic service, Consultant Led, Non-Admitted, First |
| Paediatric-Dentistry | £256.87 | £191.31 | 2021/22 National Cost Collection Data.  https://www.england.nhs.uk/publication/2021-22-national-cost-collection-data-publication/ (Accessed 9 Jan 2024). | Paediatric Dentistry service, Consultant Led, Non-Admitted, First |
| Paediatric-Urology | £174.43 | £147.83 | 2021/22 National Cost Collection Data.  https://www.england.nhs.uk/publication/2021-22-national-cost-collection-data-publication/ (Accessed 9 Jan 2024). | Paediatric Urology service, Consultant Led, Non-Admitted, First |
| Paediatric- Gastroenterology | £343.82 | £209.70 | 2021/22 National Cost Collection Data.  https://www.england.nhs.uk/publication/2021-22-national-cost-collection-data-publication/ (Accessed 9 Jan 2024). | Paediatric Gastroenterology service, Consultant Led, Non-Admitted, First |
| Paediatric - Immunology | £383.79 | £226.13 | 2021/22 National Cost Collection Data.  https://www.england.nhs.uk/publication/2021-22-national-cost-collection-data-publication/ (Accessed 9 Jan 2024). | Paediatric Immunology service, Consultant Led, Non-Admitted, First |
| Phlebotomy | £5.00 | N/A | 2021/22 National Cost Collection Data.  https://www.england.nhs.uk/publication/2021-22-national-cost-collection-data-publication/ (Accessed 9 Jan 2024). | Phlebotomy, Other Currencies tab |
| ***Adult services*** | | | | |
| Adult-General | £202.94 | £175.87 | 2021/22 National Cost Collection Data.  https://www.england.nhs.uk/publication/2021-22-national-cost-collection-data-publication/ (Accessed 9 Jan 2024). | Weighted mean of Consultant Led Non-Admitted Face-to-Face Attendance (for the face-to-face price) or Non-Face-to-Face (for the non-face-to-face price) non-paediatric services, First |
| Adult-Imaging | £52.45 | £80.92 | 2021/22 National Cost Collection Data.  https://www.england.nhs.uk/publication/2021-22-national-cost-collection-data-publication/ (Accessed 9 Jan 2024). | Diagnostic Imaging service, Consultant, Non-Admitted, First |
| Gynaecology | £215.19 | £169.85 | 2021/22 National Cost Collection Data.  https://www.england.nhs.uk/publication/2021-22-national-cost-collection-data-publication/ (Accessed 9 Jan 2024). | Gynaecology service, Consultant Led, Non-Admitted, First |
| Gastroenterology | £215.57 | £155.95 | 2021/22 National Cost Collection Data.  https://www.england.nhs.uk/publication/2021-22-national-cost-collection-data-publication/ (Accessed 9 Jan 2024). | Gastroenterology service, Consultant Led, Non-Admitted, First |
| Orthopaedics | £186.01 | £126.51 | 2021/22 National Cost Collection Data.  https://www.england.nhs.uk/publication/2021-22-national-cost-collection-data-publication/ (Accessed 9 Jan 2024). | Trauma and Orthopaedic service, Consultant Led, Non-Admitted, First |
| Dermatology | £163.39 | £114.52 | 2021/22 National Cost Collection Data.  https://www.england.nhs.uk/publication/2021-22-national-cost-collection-data-publication/ (Accessed 9 Jan 2024). | Dermatology service, Consultant Led, Non-Admitted, First |
| Physiotherapy | £115.97 | £86.53 | 2021/22 National Cost Collection Data.  https://www.england.nhs.uk/publication/2021-22-national-cost-collection-data-publication/ (Accessed 9 Jan 2024). | Physiotherapy service, Consultant Led, Non-Admitted, First |
| ENT | £175.21 | £136.03 | 2021/22 National Cost Collection Data.  https://www.england.nhs.uk/publication/2021-22-national-cost-collection-data-publication/ (Accessed 9 Jan 2024). | Ear Nose and Throat service, Consultant Led, Non-Admitted, First |
| Neurology | £296.81 | £200.18 | 2021/22 National Cost Collection Data.  https://www.england.nhs.uk/publication/2021-22-national-cost-collection-data-publication/ (Accessed 9 Jan 2024). | Neurology service, Consultant Led, Non-Admitted, Non-Admitted, First |
| Obstetrics | £254.07 | £189.41 | 2021/22 National Cost Collection Data.  https://www.england.nhs.uk/publication/2021-22-national-cost-collection-data-publication/ (Accessed 9 Jan 2024). | Obstetrics service, Consultant Led, Non-Admitted, First |
| *Note:* The table reports the unit costs of the 10 most frequently used outpatient services by children and adult participants. Although the specific unit cost of services that were less frequently used are not reported to keep the table to a manageable size, all unit costs (reported and not reported) were included in the cost calculations.  These are the services whose unit costs were not reported in the table above: 1) child outpatient services: Imaging, Child and Mental Health Services (CAMHS), MRI, Optometry, Burns, Cardiology, Dermatology, Diabetes, Endocrinology, Epilepsy, Nephrology, Neuro-disability, Neurology, Oral, Plastic, Respiratory, Physiotherapy, Podiatry, Speech and Language and Urgent Treatment Centres (UTCs).  2) adult outpatient services: Breast, Cardiology, Day case, Diabetes, Diet, Endocrinology, Genetics, Haematology, Hepatology, IAPT, Immunology, MRI, Mental, Midwife, Oncology, Ophthalmology, Oral, Paediatrics, Pain, Perinatal, Phlebotomy, Plastic surgery, Podiatry, Procedure, Respiratory, Urgent Treatment Centres (UTCs) and Urology services.  The sources of unit costs for all services (both reported and not reported) is: 2021/22 National Cost Collection Data. https://www.england.nhs.uk/publication/2021-22-national-cost-collection-data-publication/ (Accessed 9 Jan 2024). | | | | |

**Table S6.2: Unit Costs – Other Resource Use (2021/2022 P**rices)

| **Items** | **Unit cost** | **Source** | **Notes** |
| --- | --- | --- | --- |
| ***Inpatient services*** |  |  |  |
| Adult inpatient, long stay | £4,710.03 | 2021/22 National Cost Collection Data.  https://www.england.nhs.uk/publication/2021-22-national-cost-collection-data-publication/ (Accessed 9 Jan 2024). | Weighted mean of non-paediatric elective inpatients and non-elective long stay |
| Adult inpatient, short stay | £1,651.01 | 2021/22 National Cost Collection Data.  https://www.england.nhs.uk/publication/2021-22-national-cost-collection-data-publication/ (Accessed 9 Jan 2024). | Weighted mean of non-paediatric elective inpatients and non-elective short stay |
| Child inpatient, long stay | £4,363.29 | 2021/22 National Cost Collection Data.  https://www.england.nhs.uk/publication/2021-22-national-cost-collection-data-publication/ (Accessed 9 Jan 2024). | Weighted mean of paediatric elective inpatients and non-elective long stay |
| Child inpatient, short stay | £1,034.36 | 2021/22 National Cost Collection Data.  https://www.england.nhs.uk/publication/2021-22-national-cost-collection-data-publication/ (Accessed 9 Jan 2024). | Weighted mean of paediatric elective inpatients and non-elective short stay |
| ***Emergency Services*** |  |  |  |
| A&E | £241.99 | 2021/22 National Cost Collection Data.  https://www.england.nhs.uk/publication/2021-22-national-cost-collection-data-publication/ (Accessed 9 Jan 2024). | Weighted mean of all A&E attendances, except for "Emergency Medicine, Patient Dead On Arrival" |
| Ambulance | £236.81 | 2021/22 National Cost Collection Data.  https://www.england.nhs.uk/publication/2021-22-national-cost-collection-data-publication/ (Accessed 9 Jan 2024). | Weighted mean of all ambulance activities |
| ***GP & Practice Nurse*** | | | |
| GP consultation, in surgery | £37.00 | Unit Costs of Health and Social Care 2022, Personal Social Services Research Unit (University of Kent) & Centre for Health Economics (University of York), Kent, UK. https://kar.kent.ac.uk/100519/ (Accessed 9 Jan 2024) | Section 9.4.2 Unit costs for a GP With qualification costs, excluding direct care staff costs |
| GP consultation, at home | £37.00 | Unit Costs of Health and Social Care 2022, Personal Social Services Research Unit (University of Kent) & Centre for Health Economics (University of York), Kent, UK. https://kar.kent.ac.uk/100519/ (Accessed 9 Jan 2024) | Cost of home consultation not available, using in surgery consultation as proxy |
| GP consultation, telephone/video | £21.95 | Unit Costs of Health and Social Care 2022, Personal Social Services Research Unit (University of Kent) & Centre for Health Economics (University of York), Kent, UK. https://kar.kent.ac.uk/100519/ (Accessed 9 Jan 2024) | Table 9.5.1 Cost of GP telephone calls (£8.67) and GP-led triage (£12.96) |
| Practice nurse consultation,  in surgery | £7.07 | Unit Costs of Health and Social Care 2022, Personal Social Services Research Unit (University of Kent) & Centre for Health Economics (University of York), Kent, UK. https://kar.kent.ac.uk/100519/ (Accessed 9 Jan 2024) | Table 9.3.1 Hourly cost of a band 5 GP practice nurse (£46) scaled by the duration of contact of 9.72 minutes as per Hobbs et al. (2016) |
| Practice nurse consultation,  at home | £7.07 | Unit Costs of Health and Social Care 2022, Personal Social Services Research Unit (University of Kent) & Centre for Health Economics (University of York), Kent, UK. https://kar.kent.ac.uk/100519/ (Accessed 9 Jan 2024) | Cost of home consultation not available, using in surgery consultation as proxy |
| Practice nurse consultation, telephone/video | £8.69 | Unit Costs of Health and Social Care 2022, Personal Social Services Research Unit (University of Kent) & Centre for Health Economics (University of York), Kent, UK. https://kar.kent.ac.uk/100519/ (Accessed 9 Jan 2024) | Table 9.5.1 Cost of nurse-led triage |
| ***Social & Community services*** | | | |
| Social worker, adult services | £50.00 | Unit Costs of Health and Social Care 2022, Personal Social Services Research Unit (University of Kent) & Centre for Health Economics (University of York), Kent, UK. https://kar.kent.ac.uk/100519/ (Accessed 9 Jan 2024) | Section 10.1 Cost per hour, including qualifications. |
| Social worker, children services | £53.41 | Unit Costs of Health and Social Care 2022, Personal Social Services Research Unit (University of Kent) & Centre for Health Economics (University of York), Kent, UK. https://kar.kent.ac.uk/100519/ (Accessed 9 Jan 2024) | Section 11.2 Cost per hour, including qualifications. |
| Clinical psychologist | £66.00 | Unit Costs of Health and Social Care 2022, Personal Social Services Research Unit (University of Kent) & Centre for Health Economics (University of York), Kent, UK. https://kar.kent.ac.uk/100519/ (Accessed 9 Jan 2024) | Table 8.2.1. A clinical psychologist is shown as a band 7 profession in Table 8.1. |
| Children and Adolescent Mental Health Services (CAMHS) | £236.83 | Unit Costs of Health and Social Care 2022, Personal Social Services Research Unit (University of Kent) & Centre for Health Economics (University of York), Kent, UK. https://kar.kent.ac.uk/100519/ (Accessed 9 Jan 2024) | Section 6.1 CAMHS, average cost per patient contact - community contact.  2019/20 prices (£225.00) inflated to 2021/22 prices using the NHS cost inflation index (NHSCII). |
| Community Paediatrician (Children's doctor) | £341.98 | 2021/22 National Cost Collection Data.  https://www.england.nhs.uk/publication/2021-22-national-cost-collection-data-publication/ (Accessed 9 Jan 2024). | Item "Paediatric Service, Non-Admitted Face-to-Face Attendance, First" in "Outpatient Care". Community paediatricians are thought to have the same level as paediatricians in hospital and provide similar services. |
| Community children's nurse or school nurse | £140.18 | 2021/22 National Cost Collection Data.  https://www.england.nhs.uk/publication/2021-22-national-cost-collection-data-publication/ (Accessed 9 Jan 2024). | The average of school nurse (Community Health Services. Nursing, School-Based Children's Health Core Services, One to One, £138.15) and community children's nurse (Community Health Services. Nursing Services for Children, £142.21) |
| Special Education Needs Co-ordinator | £42.26 | The NASUWT, the Teachers’ Union.  https://www.nasuwt.org.uk/static/c4b7b523-eeb7-4f92-bd1fa0d8d97bb250/Teachers-Pay-Scales-2022-England-exc-London-and-the-fringe.pdf (Accessed 9 Jan 2024). | Teachers' salary with an average Special Educational Needs (SEN) Allowances and a Teaching and Learning Responsibility (TLR) payment |
| Family liaison officer - school | £57.54 | 2016/17 National Cost Collection Data.  https://www.pssru.ac.uk/project-pages/unit-costs/unit-costs-2016/ (Accessed 9 Jan 2024). | Family liaison offices are assumed to have the same salary as family support workers. The salary of the later profession is from Table 11.8. Cost per hour of client-related work. 2016/17 prices (£52.00) inflated to 2021/22 prices using the NHS cost inflation index (NHSCII). |
| Teacher (other than usual contact with your child's teacher) | £28.61 | The NASUWT, the Teachers’ Union.  https://www.nasuwt.org.uk/static/c4b7b523-eeb7-4f92-bd1fa0d8d97bb250/Teachers-Pay-Scales-2022-England-exc-London-and-the-fringe.pdf (Accessed 9 Jan 2024). | Mean annual salary of qualified teachers in England (excluding London) and Wales and accounting for information on the employer's contribution to pension schemes and National Insurance. |
| Primary mental health worker | £55.00 | Unit Costs of Health and Social Care 2022, Personal Social Services Research Unit (University of Kent) & Centre for Health Economics (University of York), Kent, UK. https://kar.kent.ac.uk/100519/ (Accessed 9 Jan 2024) | Table 8.2.1. A primary mental health worker is shown as a band 6 profession in typical job posts (example: https://www.jobs.nhs.uk/candidate/jobadvert/C9351-23-1499?sort=closingDate&page=584&language=#:~:text=The%20primary%20role%20of%20the,child%20and%20adolescent%20mental%20health) |
| Family therapist | £57.54 | 2016/17 National Cost Collection Data.  https://www.pssru.ac.uk/project-pages/unit-costs/unit-costs-2016/ (Accessed 9 Jan 2024). | Family therapists are assumed to have the same salary as family support workers. The salary of the later profession is from Table 11.8. Cost per hour of client-related work. 2016/17 prices (£52.00) inflated to 2021/22 prices using the NHS cost inflation index (NHSCII). |
| Play therapist | £67.50 | https://www.prospects.ac.uk/job-profiles/play-therapist (Accessed 9 Jan 2024). | Average session rate per hour |
| Occupational therapist, Adult services | £99.11 | 2021/22 National Cost Collection Data.  https://www.england.nhs.uk/publication/2021-22-national-cost-collection-data-publication/ (Accessed 9 Jan 2024). | Item "Occupational Therapist, Adult, One to One" in "Community Health Services" |
| Occupational therapist, Children's services | £167.91 | 2021/22 National Cost Collection Data.  https://www.england.nhs.uk/publication/2021-22-national-cost-collection-data-publication/ (Accessed 9 Jan 2024). | Item "Occupational Therapist, Child, One to One" in "Community Health Services" |
| Speech and Language therapist, Adult services | £128.16 | 2021/22 National Cost Collection Data.  https://www.england.nhs.uk/publication/2021-22-national-cost-collection-data-publication/ (Accessed 9 Jan 2024). | Item "Speech and Language Therapist, Adult, One to One" in "Community Health Services" |
| Speech and Language therapist, Children's services | £143.21 | 2021/22 National Cost Collection Data.  https://www.england.nhs.uk/publication/2021-22-national-cost-collection-data-publication/ (Accessed 9 Jan 2024). | Item "Speech and Language Therapist, Child, One to One" in "Community Health Services" |
| Physiotherapist, Adult services | £73.14 | 2021/22 National Cost Collection Data.  https://www.england.nhs.uk/publication/2021-22-national-cost-collection-data-publication/ (Accessed 9 Jan 2024). | Item "Physiotherapist, Adult, One to One" in "Community Health Services" |
| Physiotherapist, Children's services | £132.15 | 2021/22 National Cost Collection Data.  https://www.england.nhs.uk/publication/2021-22-national-cost-collection-data-publication/ (Accessed 9 Jan 2024). | Item "Physiotherapist, Child, One to One" in "Community Health Services" |
| Family support worker | £57.54 | 2016/17 National Cost Collection Data.  https://www.pssru.ac.uk/project-pages/unit-costs/unit-costs-2016/ (Accessed 9 Jan 2024). | Table 11.8. Cost per hour of client-related work. 2016/17 prices (£52.00) inflated to 2021/22 prices using the NHS cost inflation index (NHSCII). |
| Self-help support group | £0.00 | Self Help UK. 2023. Self Help Groups & Contacts. https://www.selfhelp.org.uk/directory (Accessed 14 Feb 2024). | There are a variety of free to use self-help charity groups, providing support in a variety of areas. |
| Complementary therapist | £77.50 | NHS. Homeopathy. https://www.nhs.uk/conditions/homeopathy/ (Accessed 9 Jan 2024). | The price for a consultation with a homeopath (a typical complementary therapy) can vary from around £30 to £125. Mean price is considered here. |
| Counsellor | £103.75 | Unit Costs of Health and Social Care 2021, Personal Social Services Research Unit (University of Kent), Kent, UK.  https://www.pssru.ac.uk/project-pages/unit-costs/unit-costs-of-health-and-social-care-2021/ (Accessed 9 Jan 2024). | Section 6.10 average cost per client-related hour. 2020/21 prices (£101) inflated to 2021/22 prices using the NHS cost inflation index (NHSCII). |
| ***Medications*** | | | |
| Out-of-pocket prescription charges per prescribed item | £9.35 | 2021/22 prescription charges for England https://faq.nhsbsa.nhs.uk/knowledgebase/article/KA-01375/en-us#:~:text=Historical%20prescription%20charges%3A,for%202019%2F20%3A%20%C2%A39.00 (Accessed 9 Jan 2024) | Prescription charges only apply to adults. Children under 16 are eligible for a free prescription. https://northeastlondon.icb.nhs.uk/your-health/support-for-nhs-costs/free-nhs-prescriptions/ |
| NHS prescription costs | BNF01: £5.71 BNF02: £4.84 BNF03: £14.47 BNF04: £6.93 BNF05: £5.26 BNF06: £13.04 BNF07: £7.82 BNF08: £37.16 BNF09: £11.42 BNF10: £5.03 BNF11: £9.98 BNF12: £6.81 BNF13: £9.7 BNF14: 10.93 | Prescription Cost Analysis – England – 2021/22 (National Summary Tables - Financial year) https://www.nhsbsa.nhs.uk/statistical-collections/prescription-cost-analysis-england/prescription-cost-analysis-england-202122 (Accessed 9 Jan 2024) |  |
| Over-the-counter (OTC) medicine | £3.46 | The economic impact of over-the-counter medicines https://www.pagb.co.uk/content/uploads/2023/07/20230712-Frontier-PAGB-OTC-Impact-Report-v1-0.pdf (Accessed 9 Jan 2024) | The report documents that each user, on average, spent £4.5 on 1.3 packs of OTC per month in 2022. This means the average price per pack is £3.46 (£4.5/1.3). |
| ***Opportunity cost of travelling and absenteeism*** | | | |
| Gross salary | Hourly rate:  £16 for men £13.59 for women Daily rate: £124.64 for men £89.6 for women | 2022 Earnings and hours worked, all employees: ASHE Table 1 https://www.ons.gov.uk/employmentandlabourmarket/peopleinwork/earningsandworkinghours/datasets/allemployeesashetable1 (accessed 23 Feb 2024) | The median is used for both hourly and daily rates. Daily rates were converted from the weekly rate under the assumption of 5 working days per week. |
| Daily school absence opportunity cost | £34.26 | School funding statistics https://explore-education-statistics.service.gov.uk/find-statistics/school-funding-statistics (accessed 23 Feb 2024) | Per pupil funding in 2021/22 (£6,510) divided by 190 school days |
| Daily loss of human capital due to school absence | £274.76 | Psacharopoulos et al (2021) | Based on a theoretical model proposed by Psacharopoulos et al (2021). We calibrated the model with the UK annual gross salary in 2022 and the discount rate (3.5%). |

**Table S7. Incremental Cost, Incremental QALYs and Cost-effectiveness of OSI+TS Compared to USP - Sensitivity Analyses**

|  | **Description** | | **Incremental Cost (£)** | | **Incremental QALY** | | **ICER** | **Prob. Cost-effective** | |
| --- | --- | --- | --- | --- | --- | --- | --- | --- | --- |
|  | **Cost** | **QALY** | **Mean** | **95% CI** | **Mean** | **95% CI** |  | **£20,000 WTP** | **£30,000 WTP** |
| SA1 (MCMC) | Child NHS | Child UK value set | 290.48 | (122.21,469.88) | 0.022 | (0.009,0.035) | 13,215.09 | 0.821 | 0.948 |
| SA2 (MCMC) | Child NHS | Child AUS value set | 290.48 | (122.21,469.88) | 0.043 | (0.018,0.066) | 6,704.27 | 0.979 | 0.993 |
| SA3 | Child Societal (w/o. human capital cost) | Child UK value set | 222.67 | (52.84,390.88) | 0.021 | (0.009,0.034) | 10,453.27 | 0.905 | 0.976 |
| SA4 | Child Societal (w/o. human capital cost) | Child AUS value set | 222.67 | (52.84,390.88) | 0.043 | (0.020,0.063) | 5,199.04 | 0.995 | 0.999 |
| SA5 | Child Societal (w. human capital cost) | Child UK value set | 127.72 | (-68.68,409.85) | 0.021 | (0.009,0.034) | 5,995.90 | 0.923 | 0.978 |
| SA6 | Child Societal (w. human capital cost) | Child AUS value set | 127.72 | (-68.68,409.85) | 0.043 | (0.020,0.063) | 2,982.12 | 0.992 | 0.998 |
| SA7 | Parent-child NHS | Parent-child QALY (UK value set) | 320.42 | (-20.27,638.06) | 0.021 | (-0.008,0.045) | 14,957.24 | 0.599 | 0.720 |
| SA8 | Parent-child NHS | Parent-child QALY (AUS value set) | 320.42 | (-20.27,638.06) | 0.043 | (0.006,0.073) | 7,367.29 | 0.880 | 0.942 |
| SA9 | Parent-child societal (w/o. human capital cost) | Parent-child QALY (UK value set) | 313.88 | (-132.89,709.40) | 0.021 | (-0.008,0.045) | 14,652.10 | 0.592 | 0.716 |
| SA10 | Parent-child societal (w/o. human capital cost) | Parent-child QALY (AUS value set) | 313.88 | (-132.89,709.40) | 0.043 | (0.006,0.073) | 7,217.00 | 0.873 | 0.938 |
| SA11 | Parent-child societal (w/o. human capital cost) + screening cost | Parent-child QALY (UK value set) | 313.87 | (-132.93,709.36) | 0.021 | (-0.008,0.045) | 14,651.81 | 0.592 | 0.716 |
| SA12 | Parent-child societal (w/o. human capital cost) + screening cost | Parent-child QALY (AUS value set) | 313.87 | (-132.93,709.36) | 0.043 | (0.006,0.073) | 7,216.85 | 0.873 | 0.938 |
| SA13 | Parent-child societal (w. human capital cost) | Parent-child QALY (UK value set) | 234.69 | (-236.21,695.71) | 0.021 | (-0.008,0.045) | 10,955.40 | 0.651 | 0.750 |
| SA14 | Parent-child societal (w. human capital cost) | Parent-child QALY (AUS value set) | 234.69 | (-236.21,695.71) | 0.043 | (0.006,0.073) | 5,396.16 | 0.893 | 0.945 |
| SA15 (per protocol) | Child NHS | Child UK value set | 275.64 | (115.10,491.61) | 0.030 | (0.015,0.041) | 9,315.57 | 0.954 | 0.996 |
| SA16 (per protocol) | Child NHS | Child AUS value set | 275.64 | (115.10,491.61) | 0.056 | (0.028,0.076) | 4,919.24 | 0.999 | 1.000 |
| SA17 (complete case) | Child NHS | Child UK value set | 274.52 | (66.20,482.18) | 0.028 | (0.015,0.047) | 9,798.62 | 0.960 | 0.991 |
| SA18 (complete case) | Child NHS | Child AUS value set | 274.52 | (66.20,482.18) | 0.050 | (0.024,0.078) | 5,450.83 | 0.994 | 0.998 |
| SA19 (complete case controlling for screening assessment scores) | Child NHS | Child UK value set | 276.28 | (62.73,482.16) | 0.028 | (0.016,0.047) | 9,957.14 | 0.964 | 0.994 |
| SA20 (complete case controlling for screening assessment scores) | Child NHS | Child AUS value set | 276.28 | (62.73,482.16) | 0.05 | (0.025,0.078) | 5,566.37 | 0.995 | 0.999 |
| *Notes*: SA1-SA16 are is based on 200 bootstraps of 50 multiply imputed datasets. SA17-SA20 are based on complete cases with 5,000 bootstraps. 95% CIs are constructed from the distribution of bootstrapped estimates using a percentile approach. QALY=quality-adjusted life year. ICER = incremental cost-effectiveness ratio. WTP = willingness-to-pay. SA=sensitivity analysis. OSI+TS= Online Support and Intervention for Child Anxiety with therapist support. USP=usual school practice. | | | | | | | | | |

**Table S7 - Remarks:**

SA1 & SA2 report the results obtained when multiple imputations were conducted with the Markov chain Monte Carlo (MCMC) algorithm. While multiple imputation with clusters is known to be challenging, an R program, *PanImpute*, can facilitate this process using the MCMC algorithm. However, this algorithm inevitably generated negative values for cost data by design, which contradicted the nature of this variable. In contrast, the problem of negative costs can be avoided by using the MICE algorithm costs with the predictive mean matching (PMM) method. Hence, the latter approach was adopted in most analyses (base case analyses & SA3-SA18) in this paper.

Nevertheless, SA1 & SA2 replicate the base case analyses using the datasets multiply imputed by with MCMC algorithm. Their results remain qualitatively unchanged compared to those reported in the base case analyses in Table 3.

SA11 and SA12 take the cost of the pre-intervention screening programme in school into account. The screening activities incurred some costs from the societal perspective as they were not funded by the NHS. Ideally, the screening cost would not contribute much to the cost differences between arms as the screening was implemented in all participating schools. To verify this, we re-estimated the analyses in SA 9-10 from a societal perspective with additional screening costs for each child. The results (SA 11-12) show minimal impacts on the estimates compared to those in SA 9-10, which confirms our expectations.

SA13 & SA14 present the situation where the cost of human capital loss due to school absence is taken into account. Special attention is paid to this cost because of the high risk of school absence that is associated with child anxiety problems. The school absence is costly due to 1) the unused pre-determined governmental educational spending and 2) the unrealised future labour market return in education. Supplementary Material S4 describes how to calculate the unit cost of these two sources. Table 2 summarises the cost of school absence between the two trial arms and their cost difference.

SA15 & 16 report the base-case per protocol analyses, which restricts the treated group to the patients who completed at least the first five OSI+TS modules. This excludes 102 out of 434 patients in the arm of OSI (n=332 for the OSI arm in the per protocol analysis).

SA17 & 18 report the complete case analysis based on 598 individuals (294 in OSI+TS and 304 in USP).

SA19 & 20 repeated the complete case analysis with three additional controls, the baseline clinical screening scores: the baseline clinical screening scores for child anxiety symptoms (Preschool Anxiety Scale; PAS), behavioural inhibition (Approach subscale of the Short Temperament Scale for Children; STSC-A), and parent/carer anxiety symptoms (Generalised Anxiety Disorder-7 Scale; GAD-7). These analyses examine whether the imbalance in the proportion screening positive for child anxiety, and in the size of the three-risk-factor group, biases the incremental analysis. The highly similar results, compared with SA17 & 18, argue against such a bias.

**Table S8. Net Benefits of OSI+TS Compared to USP**

|  | **Net Monetary Benefit** | | | | **Net Health Benefit** | | | |
| --- | --- | --- | --- | --- | --- | --- | --- | --- |
|  | **£20,000 WTP** | | **£30,000 WTP** | | **£20,000 WTP** | | **£30,000 WTP** | |
|  | **Mean** | **95% CI** | **Mean** | **95% CI** | **Mean** | **95% CI** | **Mean** | **95% CI** |
| Base Case 1 | 181.86 | (-126.72,470.86) | 394.87 | (-17.50,792.95) | 0.009 | (-0.006,0.024) | 0.013 | (-0.001,0.026) |
| Base Case 2 | 612.41 | (104.35,1043.72) | 1,040.69 | (307.87,1663.45) | 0.031 | (0.005,0.052) | 0.035 | (0.010,0.055) |
| SA1 | 149.14 | (-190.93,460.64) | 368.95 | (-91.54,797.87) | 0.007 | (-0.010,0.023) | 0.012 | (-0.003,0.027) |
| SA2 | 576.08 | (16.09,1055.35) | 1,009.36 | (201.56,1708.39) | 0.029 | (0.001,0.053) | 0.034 | (0.007,0.057) |
| SA3 | 203.36 | (-106.65,500.69) | 416.37 | (2.78,825.63) | 0.010 | (-0.005,0.025) | 0.014 | (0.000,0.028) |
| SA4 | 633.90 | (125.43,1075.96) | 1,062.19 | (334.65,1694.27) | 0.032 | (0.006,0.054) | 0.035 | (0.011,0.056) |
| SA5 | 298.30 | (-102.78,637.59) | 511.32 | (12.76,949.16) | 0.015 | (-0.005,0.032) | 0.017 | (0.000,0.032) |
| SA6 | 728.85 | (131.10,1201.05) | 1,157.14 | (345.87,1819.09) | 0.036 | (0.007,0.060) | 0.039 | (0.012,0.061) |
| SA7 | 108.03 | (-588.68,713.53) | 322.25 | (-652.21,1143.53) | 0.005 | (-0.029,0.036) | 0.011 | (-0.022,0.038) |
| SA8 | 549.42 | (-295.28,1247.61) | 984.34 | (-213.45,1965.04) | 0.027 | (-0.015,0.062) | 0.033 | (-0.007,0.066) |
| SA9 | 114.56 | (-621.64,801.20) | 328.79 | (-666.12,1224.98) | 0.006 | (-0.031,0.040) | 0.011 | (-0.022,0.041) |
| SA10 | 555.96 | (-333.90,1319.29) | 990.87 | (-232.69,2022.22) | 0.028 | (-0.017,0.066) | 0.033 | (-0.008,0.067) |
| SA11 | 114.57 | (-621.60,801.25) | 328.79 | (-666.12,1225.03) | 0.006 | (-0.031,0.040) | 0.011 | (-0.022,0.041) |
| SA12 | 555.96 | (-333.92,1320.83) | 990.88 | (-232.64,2022.24) | 0.028 | (-0.017,0.066) | 0.033 | (-0.008,0.067) |
| SA13 | 193.76 | (-591.13,910.85) | 407.98 | (-621.89,1319.87) | 0.010 | (-0.030,0.046) | 0.014 | (-0.021,0.044) |
| SA14 | 635.15 | (-317.29,1438.94) | 1,070.07 | (-215.68,2139.24) | 0.032 | (-0.016,0.072) | 0.036 | (-0.007,0.071) |
| SA15 | 316.14 | (-39.16,621.13) | 612.03 | (130.14,1024.90) | 0.016 | (-0.002,0.031) | 0.020 | (0.004,0.034) |
| SA16 | 845.02 | (256.74,1290.14) | 1,405.35 | (549.68,2033.27) | 0.042 | (0.013,0.065) | 0.047 | (0.018,0.068) |
| SA17 | 285.80 | (-39.05,718.44) | 565.96 | (123.51,1163.04) | 0.014 | (-0.002,0.036) | 0.019 | (0.004,0.039) |
| SA18 | 732.73 | (151.46,1306.59) | 1,236.35 | (397.43,2077.15) | 0.037 | (0.008,0.065) | 0.041 | (0.013,0.069) |
| SA19 | 278.66 | (-32.50,730.30) | 556.12 | (140.83,1174.19) | 0.014 | (-0.002,0.037) | 0.019 | (0.005,0.039) |
| SA20 | 716.39 | (184.40,1330.87) | 1,212.72 | (447.70,2091.08) | 0.036 | (0.009,0.067) | 0.040 | (0.015,0.070) |
| *Notes*: Net benefits are calculated based on 200 bootstraps of 50 multiply imputed datasets for SA1-SA16 and 5,000 bootstraps for SA17-SA20 (complete case). 95% CIs are constructed from the distribution of bootstrapped estimates using a percentile approach. WTP = willingness-to-pay. OSI+TS= Online Support and Intervention for Child Anxiety with therapist support. USP=usual school practice. | | | | | | | | |

**Table S8 - Remarks:**

According to the NICE guideline (https://www.nice.org.uk/process/pmg36/chapter/introduction-to-health-technology-evaluation), expected net health benefits and expected net monetary benefits should be presented to inform policy.

The net health benefit can be represented as

;

The net monetary benefit can be represented as

where denotes incremental health outcomes (i.e. QALYs); denotes incremental cost and denotes a certain willingness-to-pay (WTP) threshold. In our context, a positive net benefit is in favour of OSI, suggesting OSI is cost-effective, compared to USP, against a certain willingness-to-pay (WTP) threshold. Here, we report the net monetary benefit and net health benefit under the WTP thresholds of £20,000 and £30,000, which are recommended by NICE.

**Table S9. Incremental Cost, Incremental Effectiveness and Cost-effectiveness of OSI+TS Compared to USP - Sensitivity Analyses**

|  | **Description** | | **Incremental Cost (£)** | | **Incremental Effectiveness** | | **ICER** |
| --- | --- | --- | --- | --- | --- | --- | --- |
|  | **Cost** | **Effectiveness** | **Mean** | **95% CI** | **Mean** | **95% CI** |  |
| CEA SA1 | Child Societal (w/o. human capital cost) | ADIS-P | 313.90 | (-132.89,709.40) | -0.032 | (-0.065,0.021) | -9,945.72 |
| CEA SA2 | Child Societal (w. human capital cost) | ADIS-P | 234.69 | (-236.21,695.71) | -0.032 | (-0.065,0.021) | -7,435.89 |
| CEA SA3 (Per Protocol) | Child NHS | ADIS-P | 275.64 | (115.10,491.61) | -0.037 | (-0.071,0.018) | -7,442.60 |
| *Notes*: Each incremental analysis is based on 200 bootstraps of 50 multiply imputed datasets. 95% CIs are constructed from the distribution of bootstrapped estimates. ADIS-P= Anxiety Disorder Interview Schedule for Children - Parent Interview. ICER = incremental cost-effectiveness ratio. SA=sensitivity analysis. OSI+TS= Online Support and Intervention for Child Anxiety with therapist support. USP=usual school practice. | | | | | | | |

**Figure S1. Cost-effectiveness of OSI+TS at a Potential IT Cost**

|  |  |
| --- | --- |
| *Notes:* This figure reports the impact of a potential IT cost ranging from £0-100 per user on the cost-effectiveness of OSI+TS compared to the school usual practice. The two grey lines indicate the probability of OSI+TS being cost-effective at a certain IT cost. The darker line represents the probability at a WTP threshold of £20,000/QALY, and the lighter line represents the one at a WTP threshold of £30,000/QALY. | |

**Figure S1 - Remarks**:

An IT platform is an essential component to facilitate OSI delivery, which incurs an IT cost, such as licence fees. The economics analyses presented in the main text do not consider any fee for IT due to the lack of knowledge of a definite price for IT services. To evaluate the potential effect of including an IT cost on the cost-effectiveness of OSI, we re-estimated the base-case analysis by imposing a fee for IT ranging from £0-100 on each child in the OSI group.

Supplementary Figure S1 depicts the probability of OSI being cost-effective at different IT costs. While the probability decreased with increasing IT costs, OSI+TS still remained cost-effective. With the maximum imposed price of £100 per child, the probability decreased to 71% at a WTP threshold of £20,000, and 91.72% at a WTP threshold of £30,000 for the UK adult value set. For the Australia adolescent value set, the probabilities were 97.61% at a £20,000 threshold and 99.58% at a £30,000 threshold. Based on preliminary informal discussions with potential IT companies that can support the OSI IT platform, our “educated guess” is that the fee may be well below our maximum assumed price.

**Figure S2. Cost-effectiveness Acceptability Curves for the CEA Base Case Analyses**

*Note:* This figure reports the cost-effectiveness acceptability curves of the base case analysis of the cost-effectiveness analysis. The clinical outcome is ADIS-P (Anxiety Disorder Interview Schedule for Children - Parent Interview). The curve is based on 200 bootstrap iterations of 50 multiply imputed datasets.
